# Cascade of even-denominator fractional quantum Hall states in mixed-stacked multilayer graphene

**DOI:** 10.1038/s41467-026-73155-4

**Published:** 2026-05-13

**Authors:** Yating Sha, Kai Liu, Chenxin Jiang, Dan Ye, Shuhan Liu, Zhongxun Guo, Jingjing Gao, Ming Tian, Neng Wan, Kenji Watanabe, Takashi Taniguchi, Bingbing Tong, Guangtong Liu, Li Lu, Yuanbo Zhang, Zhiwen Shi, Zixiang Hu, Guorui Chen

**Affiliations:** 1 State Key Laboratory of Micro-nano Engineering Science, Key Laboratory of Artificial Structures and Quantum Control (Ministry of Education), Tsung-Dao Lee Institute and School of Physics and Astronomy, Shanghai Jiao Tong University, Shanghai, China; 2 School of Physics, Chongqing University, Chongqing, China; 3 Chongqing Key Laboratory for Strongly Coupled Physics, Chongqing University, Chongqing, China; 4 Division of Physics and Applied Physics, Nanyang Technological University, Singapore, Singapore; 5 School of Teacher Development, Chongqing University of Education, Chongqing, China; 6 State Key Laboratory of Surface Physics and Department of Physics, Fudan University, Shanghai, China; 7 Key Laboratory of MEMS of the Ministry of Education, School of Integrated Circuits, Southeast University, Nanjing, China; 8 Research Center for Electronic and Optical Materials, National Institute for Materials Science, Tsukuba, Japan; 9 Research Center for Materials Nanoarchitectonics, National Institute for Materials Science, Tsukuba, Japan; 10 Beijing National Laboratory for Condensed Matter Physics, and Institute of Physics, Chinese Academy of Sciences, Beijing, China; 11 Hefei National Laboratory, Hefei, China

**Keywords:** Quantum Hall, Electronic properties and materials, Graphene

## Abstract

The fractional quantum Hall effect at half-filled Landau levels provides a promising route to unusual topological phases that may host non-Abelian excitations, but these states are often fragile and difficult to control experimentally. Here, we report the observation of a cascade of even-denominator fractional quantum Hall states at fillings *ν* = −5/2, −7/2, −9/2, −11/2 and −13/2, alongside numerous odd-denominator states in mixed-stacked pentalayer graphene—a system characterized by intertwined quadratic and cubic band dispersions. These even-denominator states emerge from two distinct intra-zeroth Landau levels and exhibit displacement-field tunability. At half fillings, continuous quantum phase transitions between even-denominator states, magnetic Bloch states, and composite Fermi liquids are clearly identified upon tuning external fields. Numerical calculations support possible Moore–Read type pairing for even-denominator states, although direct probes of their exchange statistics remain important for future experiments. These results establish mixed-stacked graphene as a versatile platform for tunable correlated topological phases.

## Abstract (web summary)

Multilayer graphene can host complex Landau level spectra with tunable degeneracies. Here, the authors report a cascade of even-denominator fractional quantum Hall states at filling factors ν = −5/2, −7/2, −9/2, −11/2 and −13/2 in mixed-stacked pentalayer graphene.

## Introduction

Fractional quantum Hall (FQH) states are collective two-dimensional electron phases arising under strong magnetic fields when Landau levels (LLs) are partially filled^1^. Most known FQH states occur at fillings with odd denominators (e.g., 1/3, 2/5) and can be captured by the composite fermion (CF) theory as integer quantum Hall states of bound electron–flux quasiparticles^2^. A long-standing exception is the even-denominator FQH state at filling *ν* = 5/2 in the second LL (orbital quantum number *N* = 1) of GaAs^3,4^. Several candidate states have been proposed to explain the 5/2 state^5–7^, most notably the Moore–Read Pfaffian^8^, its particle–hole conjugate (anti-Pfaffian)^9,10^, and a particle–hole symmetric (PH-Pfaffian) state^11^, all featuring *p*-wave paired CFs^12^ and hosting non-Abelian quasiparticles that are central for topological quantum computation^7,13^. Despite decades of study, unambiguous experimental proof of the non-Abelian nature of the 5/2 state remains elusive^14,15^. One major challenge is the fragility and limited tunability of even-denominator states in conventional GaAs heterostructures^16^. Recently, strong even-denominator FQH states have been observed in wide GaAs quantum wells^17–20^, and direct experimental probes of their quasiparticle statistics are becoming increasingly feasible.

Recent advances in van der Waals materials, particularly graphene-based systems, have opened new avenues for exploring FQH states. In particular, multilayer graphene can host complex Landau level spectra with tunable degeneracies^21,22^. Bilayer graphene was predicted to support a Pfaffian-like half-filled state in its *N* = 1 LL^23^, and experimental evidence of the 5/2 state has been observed in ultra-clean bilayer graphene devices^24–30^. More recently, even-denominator states have been reported in trilayer graphene^31^ and other multicomponent quantum Hall systems^32,33^. Multilayer graphene offers unique advantages: the large degeneracy of the zeroth Landau levels (ZLLs) in multilayer graphene promotes a variety of spontaneously broken symmetries, producing conditions for exotic particles. Recently, non-Abelian FQH states have been predicted at both odd and even filling fractions of the highly degenerate LLs in rhombohedral multilayer graphene^34,35^. Moreover, the orbital composition of LL wavefunctions can be controlled by an external perpendicular electric field (displacement field *D*)^36,37^. By tuning *D*, one can mix LL orbitals and adjust Coulomb interactions^25,37,38^, potentially stabilizing Moore–Read states. This tunability has already enabled observations of sequences of even- and odd-denominator states and related phase transitions in Bernal-stacked multilayer graphene^25,31^.

**Fig. 1:**
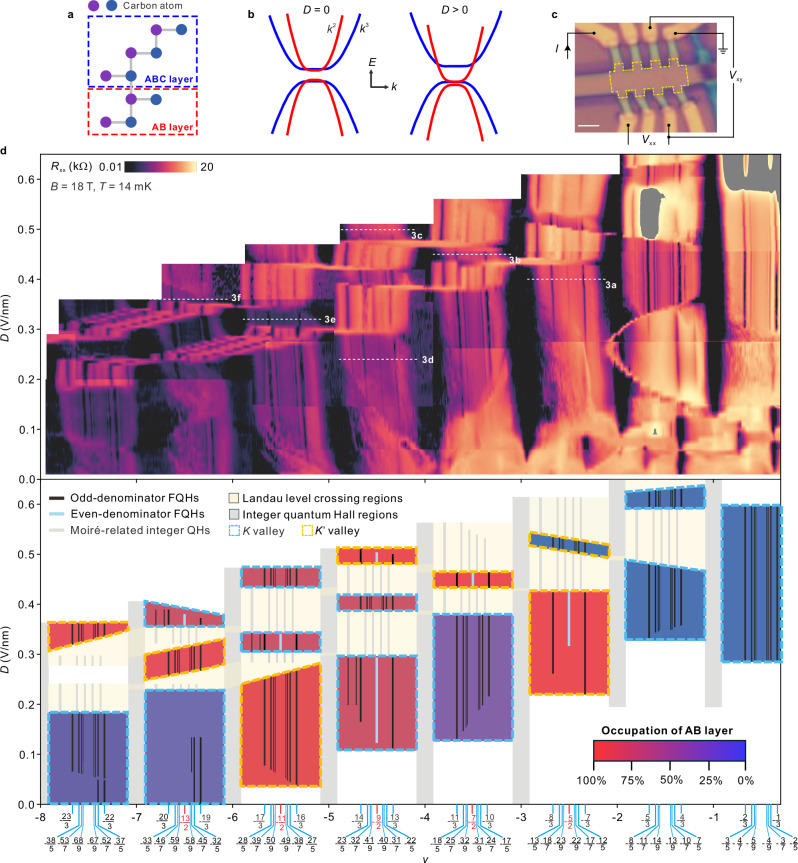
Cascade of FQH states in ABCBC-5LG under large + *D.* **a** Stacking configuration of ABCBC-5LG, which can be conceptually understood as a composition of an AB-2LG (outlined in the red box) and an ABC-3LG graphene (outlined in the blue box). **b** Schematic band structure of ABCBC-5LG at *D* = 0 and *D* > 0. The parabolic bands (red) and cubic bands (blue) originate from the bilayer and trilayer parts, respectively, as shown in (**a**). The hybridization between the two bands is neglected for simplicity. **c** Optical image of the graphite/hBN/ABCBC-5LG/hBN/graphite device (sample S1), including a schematic of the transport measurement configuration. The Hall bar-shaped graphene is outlined by the yellow dashed line. Scale bar: 2 μm. **d** *R*_xx_ as a function of filling factor *v* and displacement field *D* at *T* = 14 mK and *B* = 18 T. Well-resolved *R*_xx_ minima are observed at fractional fillings with both even- and odd-denominators. Landau level crossings are characterized by increased *R*_xx_ at integer fillings, followed by phase transitions between odd- and even-denominator sequences of FQH states. Bottom panel: sketch map of the top panel. Gray (yellow) shaded regions correspond to the integer quantum Hall states (Landau level crossing region). Gray lines in the schematic denote moiré-induced integer quantum Hall states (Hofstadter minibands), corresponding to the green lines labeled with Chern numbers in Supplementary Fig. 4d. The light blue- and dark yellow dashed lines enclosed areas are occupied by broken-symmetry states with valley index *K* and *K*’, respectively, whereas the color-filled regions denote the occupation of the AB layer. FQHs fractional quantum Hall states, QHs quantum Hall states.

ABCBC-stacked pentalayer graphene (ABCBC-5LG), one of the mixed-stacking sequences with non-centrosymmetric lattice^39–42^ (Supplementary Fig. 1), can be regarded as a stack of an ABC-trilayer and an AB-bilayer graphene sheets (Fig. 1a). The ABCBC stacking sequence inherently lacks inversion and mirror symmetry due to the inequivalent chemical environments of the carbon atoms, creating built-in displacement fields. As a result, its band structure combines cubic and parabolic band dispersions near the Fermi level with a small intrinsic gap (Fig. 1b, left), and undergoes a relative energy shift between the cubic and parabolic bands under external perpendicular displacement field *D* (Fig. 1b, right). The basic transport behavior of the ABCBC multiband has been identified in our recent work^43^. This hybrid double-layer structure creates a hierarchy of ABC- and AB-graphene’s degenerate LLs with distinct orbital components, enabling tunable LL crossings, hybridizations, and interactions between different bands and layers under electric or magnetic fields^22,31,44^. Unlike simpler graphene systems, the multiband nature of ABCBC-5LG amplifies LL mixing effects, which govern the stability and character of fractional quantum Hall states. Moreover, dual electrostatic gating (see Methods) enables independent tuning of carrier density (*n*) and displacement field (*D*, corresponding to interlayer polarization), offering powerful experimental knobs to control phase transitions between competing orders.

In this work, we leverage these properties to uncover a rich spectrum of FQH states in ABCBC-5LG. By applying high *B-*field and *D*-field, we reveal a cascade of even-denominator FQH states at half-fillings of certain two intra-ZLLs, interwoven with conventional odd-denominator sequences. We demonstrate that the electric displacement field *D* can drive transitions between odd-denominator and even-denominator states by inducing Landau level crossings. Among the even-denominator states, two record-high filling factors, −11/2 and −13/2, in ZLLs are reported. Those states coexist with odd-denominator sequences that bifurcate into several hierarchical branches, including one associated with 2-flux CFs, reflecting the interplay between multi-orbital LLs and interactions. Our device also showcases continuous quantum phase transitions, including candidate non-Abelian and Abelian FQH states, as well as a magnetic Bloch state. These results establish mixed-stacked graphene as a promising platform for exploring exotic correlated and topological states, which can host flat band features similar to rhombohedral graphene^45–49^, while the hybridization between subbands may lead to even richer states.

## Results

### Phase diagram in a high magnetic field

Our devices consist of ABCBC-5LG encapsulated in hexagonal boron nitride (hBN) and graphite top and bottom gates, and contacted via one-dimensional edge metal electrodes^50^. All data in the main text are from sample S1 (Fig. 1c, Supplementary Figs. 2 and 3). Under application of a high magnetic field (*B* = 18 T), the degeneracy of Landau levels is fully lifted, yielding robust integer quantum Hall (IQH) gaps from *ν* = −1 to −8 and a rich spectrum of LLs that can cross and hybridize as a function of *D*. Figure 1d shows the longitudinal resistance *R*_xx_ measured as a function of filling factor *ν* = *nh/*(*eB*) (*n* is the carrier density, *h* is Planck’s constant, *e* is the electron charge) and displacement field *D* at base temperature (*T* = 14 mK) and *B* = 18 T. Black regions indicate *R*_xx_ minima, corresponding to robust quantum Hall states. In particular, we observe pronounced minima at numerous fractional fillings, including both odd- and even-denominator fractions up to eight intra-ZLLs (limited by gate leakage). We label the observed integer and fractional quantum Hall states in the lower panel of Fig. 1d, where the IQH states are labeled by gray regions. Notably, even-denominator FQH states, including *ν* = −5/2, −7/2, −9/2, −11/2, and −13/2, are highlighted by light blue lines. They are flanked by nearby odd-denominator states (black lines) that organize into Jain sequences with two flux quanta (CF_2_), together with further fractional features at nearby fillings beyond the simple CF_2_ hierarchy.

The emergence of these half-filled states is strongly tied to Landau level crossings. As *D* is varied, the orbital character of LLs shifts, and we observe enhanced *R*_xx_ at certain integer *ν* where LL crossings occur. These crossings trigger phase transitions between different FQH sequences: on one side of a crossing, odd-denominator Jain-series states dominate, whereas on the other side, the even-denominator half-filled state emerges as a robust gap. In Fig. 1d, such transitions manifest as a sequence of black lines (odd-denominator states) evolving to a central light blue line (an incompressible state at half-filling) with adjacent fractions suppressed. This behavior indicates that tuning *D* drives the system into regimes of strong LL mixing where half-filled states consistent with Moore–Read type order are energetically favored, as supported by our numerical analysis (Supplementary Notes).

**Fig. 2:**
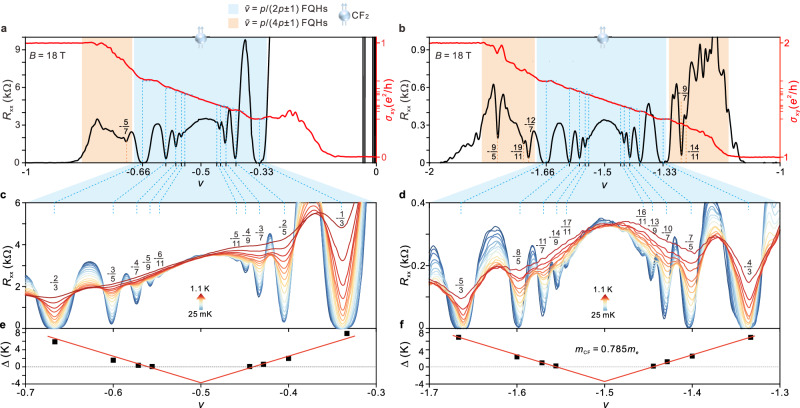
Energy gaps of odd-denominator FQH states and the effective mass of the CF_2_ quasiparticles. **a**, **b** Linecuts of *R*_xx_ and *σ*_xy_ for the FQH states within the filling ranges of −1 ⩽ *ν* ⩽ 0 and −2 ⩽ *ν* ⩽ −1, respectively, measured at *B* = 18 T and *D* = +0.4 V/nm. The Jain sequence of two-flux CFs (CF_2_) is evident in the blue region, while several states (possible four-flux CFs) lying beyond the primary CF_2_ hierarchy are also observed in the orange shading region. **c**, **d** Temperature dependence of *R*_xx_ for the sequence of CF_2_ states shown in (**a**, **b**), respectively. **e**, **f** Corresponding energy gaps for various FQH states, determined from Arrhenius fitting (Supplementary Fig. 7). The gaps follow a linear fit (red straight line in the lower panels): *Δ* = ℏ*eB*_eff_/*m*_CF_, where *B*_eff_ is the effective magnetic field for CFs and *m*_CF_ is the composite fermion mass. *m*_CF_ is found to be 0.785 *m*_*e*_.

Away from the special half-filling conditions, the Jain CF hierarchy is largely intact, demonstrating the high quality of our device. Figure 2a, b shows linecuts of *R*_xx_ (and corresponding Hall conductivity *σ*_xy_) at fixed *D* = +0.4 V/nm for filling ranges −1 ≤ *ν* ≤ 0 and −2 ≤ *ν* ≤ −1. In these regimes, we resolve a standard sequence of CF_2_ states with −1/3, −2/3, −2/5, −3/5, −3/7, −4/7, −4/9, −5/9, −5/11, −6/11, and −4/3, −5/3, −7/5, −8/5, −10/7, −11/7, −13/9, −14/9, −16/11, −17/11 (blue regions in Fig. 2a, b, following the standard fillings *ṽ* = *v*−[*v*] = *p*/(2*p* ± 1) where [*v*] being the largest integer number less than *v* and *p* = 1,2,3…). We also observe several weaker states at higher fillings near −5/7, −9/5, −9/7, −12/7, −14/11, and −19/11 (orange regions, corresponding to *ṽ* = *p*/(4*p* ± 1)). All these CF_2_ states show quantized Hall plateaus (in *σ*_xy_) and thermally activated behavior in *R*_xx_. Figure 2c, d presents the temperature dependence of *R*_xx_ for representative states. Activation gaps extracted from Arrhenius fitting (Fig. 2e, f) follow the expected linear trend, consistent with CF theory and suggesting a CF_2_ liquid at the half-filling. From a linear fit *Δ* = ℏ*eB*_eff_/*m*_CF_ (where *B*_eff_ is the effective magnetic field seen by CFs), we estimate an effective CF mass *m*_CF_ = 0.785 *m*_e_ (where *m*_e_ is the free electron mass), comparable to the value previously reported in monolayer graphene^51^. The extrapolated gaps approaching half-filling (e.g., *ν* = −1/2 and −3/2) tend to be negative, attributed to disorder-induced broadening of the CF LLs^52–54^. The intercept of the linear fit yields a broadening parameter *Γ* ~ 4 K, even smaller than that previously reported in Corbino^55^ and graphite gate defined graphene^56^.

### Two groups of even-denominator FQHs

**Fig. 3:**
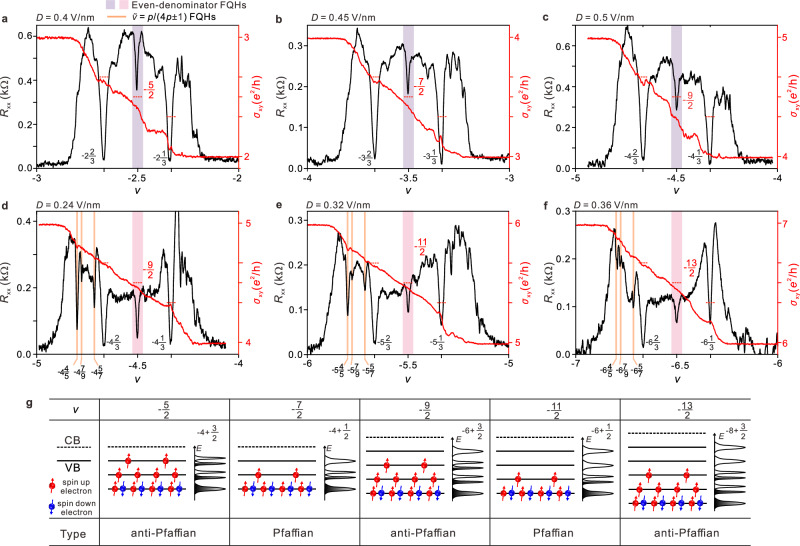
Two sets of even-denominator FQH states. Linecuts of *R*_xx_ and *σ*_xy_ at *B* = 18 T within the filling ranges of −3 ⩽ *ν* ⩽ −2 (**a**), −4 ⩽ *ν* ⩽ −3 (**b**), −5 ⩽ *ν* ⩽ −4 (**c**, **d**), −6 ⩽ *ν* ⩽ −5 (**e**), and −7 ⩽ *ν* ⩽ −6 (**f**), respectively. The displacement field *D* for each panel is indicated by white dashed lines in Fig. 1d. Even-denominator FQH states at *ν* = −5/2, −7/2, −9/2, −11/2, and −13/2 are clearly resolved, accompanied by a strong suppression of odd-denominator Jain states. We categorize the observed half-filling states into two groups, highlighted by purple shading in (**a**–**c**) and pink shading in (**d**, **e**). Phenomenologically, a hallmark of the second group is the appearance of several states beyond the CF_2_ sequence (orange shading) accompanying each half-filled state on the left, in contrast to the first group, where such states are absent. Similar features were reproducibly observed at opposite field direction, namely at −18 T (Supplementary Fig. 6). **g** Identification of the predicted Moore–Read type for all observed half-filled states based on the calculations (Supplementary Notes), along with schematic illustrations of the relevant fillings. Solid and dashed lines represent LLs in the valence and conduction bands, respectively. Red and blue spheres denote electrons, with arrows indicating their spin.

Focusing on the half-filled states, Fig. 3 provides detailed linecuts of *R*_xx_ and *σ*_xy_ in the vicinity of each even-denominator fraction corresponding to white dashed lines in Fig. 1d. We identify two groups of half-filled states, each associated with a different intra-ZLL: one group includes *ν* = −5/2, −7/2, and −9/2 (Fig. 3a–c, purple shading), and the other includes *ν* = −9/2, −11/2, and −13/2 (Fig. 3d–f, pink shading). In each case, the even-denominator state appears as a pronounced *R*_xx_ minimum, while nearby Jain odd-denominator states are suppressed^3^. This indicates that the system favors the possible paired CF state over the sequence of single CF states in that filling range. By tracing the evolution of the LLs under *D*-tuning, we confirmed that the purple group (−5/2, −7/2, and −9/2) and pink group (−9/2, −11/2, and −13/2) correspond to two distinct intra-ZLLs crossing the Fermi level. Starting from the states at −5/2 (*D* = 0.4 V/nm) and −9/2 (*D* = 0.24 V/nm), each time a LL crossing occurs as *D* increases, the corresponding even-denominator state moves to a higher-index LL, following an upward-left trend (pink/purple shading in Supplementary Fig. 5a, lower panel). At fixed half-filling of a certain LL, such as the fifth LL, by tuning *D*, the ground state can be continuously switched from FQH gapped state (−9/2) to composite Fermi liquid (CFL)^57^ to FQH gapped state again. Each switch corresponds to an LL crossing. This behavior underscores a key result: the half-filled FQH state can repeatedly emerge in the same or different LLs in a single device, given appropriate tuning, forming a cascade of even-denominator FQH states. Notably, the cascade persists up to *ν* = −11/2 and −13/2 half-filled states—remarkably high in the ZLLs—highlighting the extended stability of such states across multiple intra-ZLLs in mixed-stacked multilayer graphene.

To elucidate the nature of the observed even-denominator FQH states, we performed numerical calculations that incorporate three key physical effects: Coulomb interactions, LL mixing, and *D*. The results reveal two distinct valence-band LLs with particularly large occupation by the same wavefunction with *N* = 1 Landau level in a conventional two-dimensional electron gas, which is well established to stabilize Moore–Read state^3,8^ (see Supplementary Notes), naturally accounting for the two groups of half-filled FQH states observed in our experiment. In our analysis, we consider the mixing of two adjacent relativistic LLs, which leads to two types of fillings: 1 + 1/2 and 1/2. Accordingly, the experimentally observed filling factors can be expressed as: *ν* = −4 + 3/2, −4 + 1/2, −6 + 3/2, −6 + 1/2, and -8 + 3/2 as shown in Fig. 3g. All these cases exhibit six-fold quasi-degenerate ground states in our numerical calculations—a hallmark of the Moore–Read type state. To gain deeper insight into their particle–hole symmetry, we compute the chiral graviton spectral functions. By comparing the spectral responses, we find that the states at −5/2, −9/2, and −13/2 tend toward the anti-Pfaffian phase. In contrast, the states at −7/2 and −11/2 exhibit characteristics more consistent with the Pfaffian phase.

Theoretical analyses^6^ have proposed a hierarchy of daughter states arising from *e*/4 excitations of the anti-Pfaffian phase, with the simplest ones predicted at *ν* = 6/13 and 9/17. As shown in Supplementary Fig. 8, weak *R*_xx_ dips at *ν* = −2 − 9/17, −4 − 6/13, −4 − 9/17 are observed in the vicinity of −5/2 and −9/2, suggesting the possible existence of an anti-Pfaffian ground state. We note that the weak signals may be limited by the sample quality and contact issues. However, we also caution that the identification of paired states based on their daughter states is indirect and has not been independently confirmed by direct measurements, such as thermal conductance^15^ or upstream noise at interfaces^58^.

Together, these results reveal a distinctive cascade of even-denominator FQH states, shaped by band structure and LL hybridization in ABCBC-5LG.

### Phase transitions between distinct quasiparticles

**Fig. 4:**
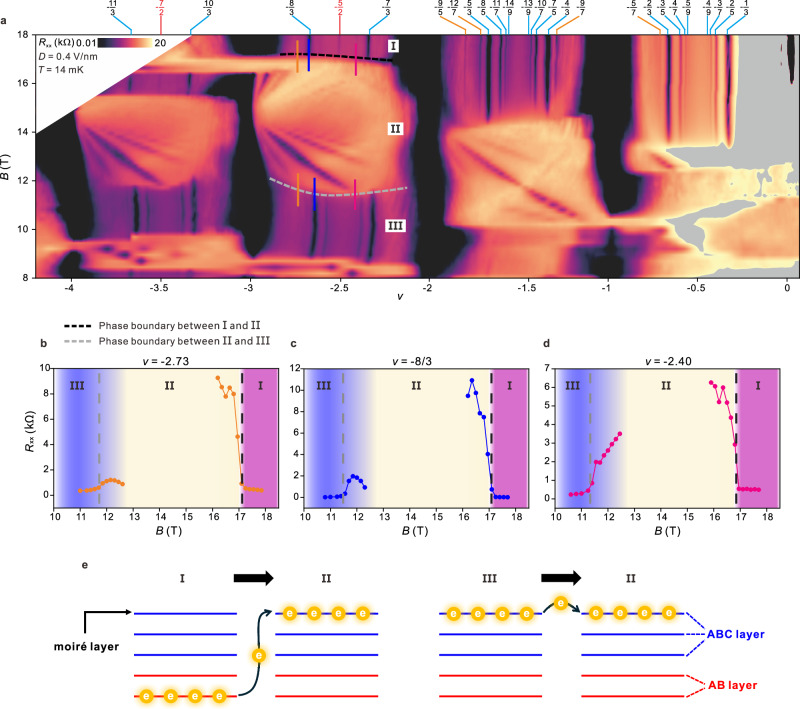
Multiple phase transitions. **a** *R*_xx_ as a function of filling *v* and magnetic field *B* at *T* = 14 mK and *D* = 0.4 V/nm. The Hofstadter butterfly pattern emerges within the region of Landau level crossings along the tuning *B*-field. **b**–**d** Linecuts of *R*_xx_ along the orange, blue, and pink lines in (**a**). The blue, yellow, and pink-filled regions represent the LL exhibiting odd-denominator FQH (III), Hofstadter minibands (II), and LL exhibiting even-denominator FQH (I), respectively. The black (gray) dashed lines are used to mark the phase boundary from region I (III) to region II. **e** Illustration of charge distributions for different quantum phases and their transitions.

To map out the LL structure more comprehensively, we also performed measurements as a function of magnetic field *B* at a fixed displacement field. Figure 4a presents *R*_xx_ vs *ν* and *B* at *D* = 0.4 V/nm, focusing on the region where LL crossings occur. As *B* is varied, we observe some dark-colored diagonal traces (representing *R*_xx_ dips) occur, accompanied by some bright-colored square-shaped regions (representing large *R*_xx_). These features relate to a Hofstadter butterfly pattern—oscillations in *R*_xx_ reflecting the fractal LL spectrum formed in a periodic moiré potential between graphene and hBN^59^. We find that these patterns are observed solely near LL crossings. This can be attributed to the relatively weak moiré potential in this device (moiré period ≈ 12.8 nm; see Supplementary Fig. 4), leading to small Hofstadter gaps that are only visible when comparable to the Landau level gap, that is, near LL crossings. Moreover, our second ABCBC-5LG device without moiré also reveals a consistent phase diagram in high-field, confirming that the observed states are intrinsic to the special stacking configuration (Supplementary Fig. 5).

Within this region, the appearance and disappearance of even-denominator states can be tracked in Fig. 4a: at certain *B* values near 16–18 T, the *ν* = −5/2 or −7/2 FQH states exist. As *B* is decreased, these FQH states disappear, and the LLs first evolve into Hofstadter miniband states, followed by the re-emergence of odd-denominator FQH and CFL states. Notably, the tunable transitions at half-filling—from even-denominator FQH states, through Hofstadter miniband regimes, to CFL phases—suggest a sequence of continuous quantum phase transitions, shaped by the interplay between interactions, magnetic flux, and lattice effects in the multilayer graphene system. We emphasize that these identifications are based on transport signatures. Direct probes of the underlying quasiparticles are left for future work.

Interestingly, we observe a qualitative difference in the transport behaviors between these phase transitions. Figure 4b–d shows linecuts of Fig. 4a at *ν* = −2.73, −8/3, −2.4 along the magnetic field, where the transition from region III (LL exhibiting odd-denominator FQH, blue shaded) to region II (Hofstadter minibands, yellow shaded) appears relatively gradual, with a slow variation in *R*_xx_ across the transition. In contrast, the transition from region I (LL exhibiting even-denominator FQH, pink shaded) to region II is significantly sharp, as indicated by a steep change in *R*_xx_. Transitions around *ν* = −7/2 also show similar behavior (Supplementary Fig. 9). The comparison suggests that the two transitions may be captured by distinct quantum phase transitions. The single-particle calculated results show that orbitals in I and III share the same valley, while I is occupied by carriers in the AB layer but III in the ABC layer (Supplementary Fig. 10c, f). Region II is dominated by Hofstadter minibands, suggesting carriers are in the topmost layer since the moiré lattice is formed between top hBN and graphene (Supplementary Fig. 3b). As illustrated in Fig. 4e, the different transition sharpness may reflect the distinct charge transfer process across the transitions. Yet the underlying mechanism deserves further investigation.

## Methods

### Sample fabrications

Graphene, graphite, and hBN are mechanically exfoliated on SiO_2_(285 nm)/Si substrates, and the layer numbers are identified using optical contrast and atomic force microscopy.

The heterostructures were assembled using a dry-transfer technique with polypropylene carbonate (PPC) or polycarbonate (PC) as the polymer, resulting in hBN/top graphite/5LG/hBN/bottom graphite stacks. The thickness of the top and bottom hBN is 47 and 42 nm, respectively, for the device in the main text. The stacks were annealed to remove interfacial contamination and trapped bubbles. SNOM imaging was then performed again to verify whether the metastable state had degraded.

Subsequently, devices were patterned into Hall bar geometries using standard electron-beam lithography, reactive ion etching (using CHF₃/O₂ plasma, O₂ plasma, or SF_6_ plasma as needed, depending on the target material), and one-dimensional Cr/Au (about 5/50 nm) edge-contact^50^ deposition.

### Stacking order identification

The stacking order of the device was unambiguously determined as ABCBC—consistent with our previous study^43^—based on two independent criteria: (i) the characteristic asymmetric displacement-field dependence of the transport gap and (ii) NanoARPES band dispersions that uniquely agree with the calculated electronic structure of the ABCBC stacking. The device investigated here is identical to that characterized previously^43^. Further details can be found therein.

### Transport measurements

Transport measurements were mainly carried out in a top-loading dilution refrigerator (with a base temperature~14 mK), equipped with superconducting magnets up to 18 T. Also, we used a fast-cooling He^4^-cryogenic system (2 K base temperature, Electronics Transport Measurement System, Model EM7, East Changing Technologies, China) to screen high-quality samples. Standard low-frequency lock-in techniques were used with an AC excitation current of 1–10 nA at ~17.777 Hz. Home-built low-pass RC filters were installed in the dilution refrigerator to suppress high-frequency noise.

The data of Hall conductivity *σ*_xy_ are obtained from the measured resistances by *σ*_xy_ = *ρ*_xy_/(*ρ*^2^_xx_ + *ρ*^2^_xy_). Gate voltages were applied using two Keithley 2400 source meters. Independent top and bottom graphite gates enabled tuning of the carrier density *n* and the out-of-plane displacement field *D* in the graphene layer. Based on a parallel-plate capacitor model: $D=({D}_{b}+{D}_{t})/2,n=({D}_{b}-{D}_{t})/e$, where ${D}_{b}=+ {\varepsilon }_{b}({V}_{b}-{V}_{b}^{0})/{d}_{b},{D}_{t}=-{\varepsilon }_{t}({V}_{t}-{V}_{t}^{0})/{d}_{t},\varepsilon$ and *d* are the dielectric constant and thickness of the dielectric layers, respectively, ${V}_{b}^{0}$ and ${V}_{t}^{0}$ are effective offset voltages caused by environment-induced carrier doping. Notably, in such devices, part of the graphene leads unavoidably lies outside the dual-gated region. To independently control the carrier density in these ungated regions, we employed the silicon substrate as a global back gate^25^ to lower contact resistance and improve the quality of magnetotransport measurements.

### Calculation of the CF effective mass

A very fundamental parameter characterizing CFs is their effective mass (*m*_CF_), which arises primarily from electron-electron interactions. The magnitude of this mass determines the energy separation between the CF LLs (sometimes referred to as Λ “levels”), and in turn determines the size of the energy gaps for the FQHSs at *ṽ* = *p*/(2*p* ± 1). To extract *m*_CF_, we measured the temperature dependence of the longitudinal resistance *R*_xx_ for a series of well-developed odd-denominator FQHSs. Activation gaps *Δ* were obtained by fitting the data to the Arrhenius form *R*_xx_ ∝ exp (−*Δ*/2*K*_B_*T*), using linear fits of ln (*R*_xx_) versus 1/*T* in the thermally activated regime. Within the CF framework, these gaps correspond to the CF cyclotron energy ℏ*ω*_CF_ = ℏ*eB*_eff_/*m*_CF_, where *B*_eff_ = *B* − *B*_half_filling_ is the effective magnetic field seen by CFs.

## Supplementary information

Supplementary Information

Transparent Peer Review file

## Data Availability

The data shown in the main figures are available from the Harvard Dataverse Repository at 10.7910/DVN/VU693N. Other data that support the plots in this paper and other findings of this study are available from the corresponding author on request.

## Code availability

The code that supports the findings of this study is available from the corresponding authors upon request. The code is not currently publicly released because it is part of an actively developing research framework and is not yet in a sufficiently documented, modularized, and user-ready form for public distribution.

## References

[1] Tsui, D. C., Stormer, H. L. & Gossard, A. C. Two-dimensional magnetotransport in the extreme quantum limit. *Phys. Rev. Lett.* **48**, 1559–1562 (1982).

[2] Jain, J. K. Composite-fermion approach for the fractional quantum Hall effect. *Phys. Rev. Lett.* **63**, 199–202 (1989). 10040805 10.1103/PhysRevLett.63.199

[3] Willett, R. et al. Observation of an even-denominator quantum number in the fractional quantum Hall effect. *Phys. Rev. Lett.* **59**, 1776–1779 (1987). 10035326 10.1103/PhysRevLett.59.1776

[4] Pan, W. et al. Exact quantization of the even-denominator fractional quantum Hall state at *v* = 5/2 Landau level filling factor. *Phys. Rev. Lett.* **83**, 3530–3533 (1999).

[5] Haldane, F. D. M. & Rezayi, E. H. Spin-singlet wave function for the half-integral quantum Hall effect. *Phys. Rev. Lett.* **60**, 956–959 (1988). 10037900 10.1103/PhysRevLett.60.956

[6] Levin, M. & Halperin, B. I. Collective states of non-Abelian quasiparticles in a magnetic field. *Phys. Rev. B* **79**, 205301 (2009).

[7] Wen, X. G. Non-Abelian statistics in the fractional quantum Hall states. *Phys. Rev. Lett.* **66**, 802–805 (1991). 10043904 10.1103/PhysRevLett.66.802

[8] Moore, G. & Read, N. Nonabelions in the fractional quantum Hall effect. *Nucl. Phys. B* **360**, 362–396 (1991).

[9] Levin, M., Halperin, B. I. & Rosenow, B. Particle-hole symmetry and the Pfaffian state. *Phys. Rev. Lett.* **99**, 236806 (2007). 18233396 10.1103/PhysRevLett.99.236806

[10] Lee, S. S., Ryu, S., Nayak, C. & Fisher, M. P. A. Particle-hole symmetry and the *v* = 5/2 quantum Hall state. *Phys. Rev. Lett.* **99**, 236807 (2007). 18233397 10.1103/PhysRevLett.99.236807

[11] Zucker, P. T. & Feldman, D. E. Stabilization of the particle-hole Pfaffian order by Landau level mixing and impurities that break particle-hole symmetry. *Phys. Rev. Lett*. **117**, 096802 (2016). 10.1103/PhysRevLett.117.096802 27610872

[12] Greiter, M., Wen, X. G. & Wilczek, F. Paired Hall state at half filling. *Phys. Rev. Lett.* **66**, 3205–3208 (1991). 10043726 10.1103/PhysRevLett.66.3205

[13] Nayak, C., Simon, S. H., Stern, A., Freedman, M. & Das Sarma, S. Non-Abelian anyons and topological quantum computation. *Rev. Mod. Phys.* **80**, 1083–1159 (2008).

[14] Willett, R. L., Pfeiffer, L. N. & West, K. W. Measurement of filling factor 5/2 quasiparticle interference with observation of charge e/4 and e/2 period oscillations. *Proc. Natl. Acad. Sci. USA* **106**, 8853–8858 (2009). 19433804 10.1073/pnas.0812599106 PMC2690045

[15] Banerjee, M. et al. Observation of half-integer thermal Hall conductance. *Nature* **559**, 205–210 (2018). 29867160 10.1038/s41586-018-0184-1

[16] Halperin, B. I., Stern, A., Neder, I. & Rosenow, B. Theory of the Fabry-Pérot quantum Hall interferometer. *Phys. Rev. B* **83**, 155440 (2011).

[17] Suen, Y. W., Manoharan, H. C., Ying, X., Santos, M. B. & Shayegan, M. Origin of the *v* =1/2 fractional quantum Hall state in wide single quantum wells. *Phys. Rev. Lett.* **72**, 3405–3408 (1994). 10056190 10.1103/PhysRevLett.72.3405

[18] Singh, S. K. et al. Topological phase transition between Jain states and daughter states of the *v* = 1/2 fractional quantum Hall state. *Nat. Phys.* **20**, 1247–1252 (2024).

[19] Wang, C. et al. Even-denominator fractional quantum Hall state at filling factor *v* = 3/4. *Phys. Rev. Lett.* **129**, 156801 (2022). 36269975 10.1103/PhysRevLett.129.156801

[20] Wang, C. et al. Next-generation even-denominator fractional quantum Hall states of interacting composite fermions. *Proc. Natl. Acad. Sci. USA* **120**, e2314212120 (2023). 38113254 10.1073/pnas.2314212120 PMC10756197

[21] Koshino, M. & McCann, E. Landau level spectra and the quantum Hall effect of multilayer graphene. *Phys. Rev. B* **83**, 165443 (2011).

[22] Taychatanapat, T., Watanabe, K., Taniguchi, T. & Jarillo-Herrero, P. Quantum Hall effect and Landau-level crossing of Dirac fermions in trilayer graphene. *Nat. Phys.* **7**, 621–625 (2011).

[23] Apalkov, V. M. & Chakraborty, T. Stable Pfaffian state in bilayer graphene. *Phys. Rev. Lett.* **107**, 186803 (2011). 22107662 10.1103/PhysRevLett.107.186803

[24] Ki, D. K., Fal’ko, V. I., Abanin, D. A. & Morpurgo, A. F. Observation of even denominator fractional quantum Hall effect in suspended bilayer graphene. *Nano Lett.* **14**, 2135–2139 (2014). 24611523 10.1021/nl5003922

[25] Maher, P. et al. Tunable fractional quantum Hall phases in bilayer graphene. *Science* **345**, 61–64 (2014). 24994646 10.1126/science.1252875

[26] Li, J. I. A. et al. Even-denominator fractional quantum Hall states in bilayer graphene. *Science* **358**, 648–652 (2017). 28982799 10.1126/science.aao2521

[27] Zibrov, A. A. et al. Tunable interacting composite fermion phases in a half-filled bilayer-graphene Landau level. *Nature* **549**, 360–364 (2017). 28933427 10.1038/nature23893

[28] Huang, K. et al. Valley isospin controlled fractional quantum hall states in bilayer graphene. *Phys. Rev. X* **12**, 031019 (2022).

[29] Assouline, A. et al. Energy gap of the even-denominator fractional quantum Hall state in bilayer graphene. *Phys. Rev. Lett.* **132**, 046603 (2024). 38335366 10.1103/PhysRevLett.132.046603

[30] Kumar, R. et al. Quarter- and half-filled quantum Hall states and their topological orders revealed by daughter states in bilayer graphene. *Nat. Commun.* **16**, 7255 (2025). 40769967 10.1038/s41467-025-62650-9 PMC12328565

[31] Chen, Y. et al. Tunable even- and odd-denominator fractional quantum Hall states in trilayer graphene. *Nat. Commun.* **15**, 6236 (2024). 39043699 10.1038/s41467-024-50589-2 PMC11266615

[32] Falson, J. et al. Even-denominator fractional quantum Hall physics in ZnO. *Nat. Phys.* **11**, 347–351 (2015).

[33] Shi, Q. et al. Odd- and even-denominator fractional quantum Hall states in monolayer WSe_2_. *Nat. Nanotechnol.* **15**, 569–573 (2020). 32632320 10.1038/s41565-020-0685-6

[34] Wu, Y. H., Shi, T. & Jain, J. K. Non-Abelian parton fractional quantum Hall effect in multilayer graphene. *Nano Lett* **17**, 4643–4647 (2017). 28649831 10.1021/acs.nanolett.7b01080

[35] Timmel, A. & Wen, X. G. Non-Abelian Fibonacci quantum Hall states in 4-layer rhombohedral stacked graphene. Preprint at https://arxiv.org/abs/2308.09702 (2023).

[36] McCann, E. & Fal’ko, V. I. Landau-level degeneracy and quantum Hall effect in a graphite bilayer. *Phys. Rev. Lett.* **96**, 086805 (2006). 16606214 10.1103/PhysRevLett.96.086805

[37] Hunt, B. M. et al. Direct measurement of discrete valley and orbital quantum numbers in bilayer graphene. *Nat. Commun.* **8**, 948 (2017). 29038518 10.1038/s41467-017-00824-w PMC5715057

[38] Weitz, R. T., Allen, M. T., Feldman, B. E., Martin, J. & Yacoby, A. Broken-symmetry states in doubly gated suspended bilayer graphene. *Science* **330**, 812–816 (2010). 20947726 10.1126/science.1194988

[39] Min, H. & MacDonald, A. H. Chiral decomposition in the electronic structure of graphene multilayers. *Phys. Rev. B* **77**, 155416 (2008).

[40] Koshino, M. & McCann, E. Multilayer graphenes with mixed stacking structure: interplay of Bernal and rhombohedral stacking. *Phys. Rev. B* **87**, 045420 (2013).

[41] Wirth, K. G. et al. Experimental observation of ABCB stacked tetralayer graphene. *ACS Nano* **16**, 16617–16623 (2022). 36205460 10.1021/acsnano.2c06053

[42] Sarsfield, P. J., Garcia-Ruiz, A. & Fal’ko, V. I. Substrate, temperature, and magnetic field dependence of electric polarization in mixed-stacking tetralayer graphenes. *Phys. Rev. Res.* **6**, 043324 (2024).

[43] Liu, K. et al. Intrinsic layer polarization and multi-flatband transport in non-centrosymmetric mixed-stacked multilayer graphene. Preprint at https://arxiv.org/abs/2505.12478 (2025). 10.1021/acs.nanolett.6c01016 42190073

[44] Datta, B. et al. Strong electronic interaction and multiple quantum Hall ferromagnetic phases in trilayer graphene. *Nat Commun.* **8**, 14518 (2017). 28216666 10.1038/ncomms14518 PMC5321728

[45] Liu, K. et al. Spontaneous broken-symmetry insulator and metals in tetralayer rhombohedral graphene. *Nat. Nanotechnol.* **19**, 188–195 (2024). 37996516 10.1038/s41565-023-01558-1

[46] Sha, Y. et al. Observation of a Chern insulator in crystalline ABCA-tetralayer graphene with spin-orbit coupling. *Science* **384**, 414–419 (2024). 38662836 10.1126/science.adj8272

[47] Han, T. et al. Large quantum anomalous Hall effect in spin-orbit proximitized rhombohedral graphene. *Science* **384**, 647–651 (2024). 38723084 10.1126/science.adk9749

[48] Lu, Z. et al. Fractional quantum anomalous Hall effect in multilayer graphene. *Nature* **626**, 759–764 (2024). 38383622 10.1038/s41586-023-07010-7

[49] Xie, J. et al. Tunable fractional Chern insulators in rhombohedral graphene superlattices. *Nat. Mater.* **24**, 1042–1048 (2025). 40263582 10.1038/s41563-025-02225-7

[50] Wang, L. et al. One-dimensional electrical contact to a two-dimensional material. *Science* **342**, 614–617 (2013). 24179223 10.1126/science.1244358

[51] Polshyn, H. et al. Quantitative transport measurements of fractional quantum Hall energy gaps in edgeless graphene devices. *Phys. Rev. Lett.* **121**, 226801 (2018). 30547606 10.1103/PhysRevLett.121.226801

[52] Du, R. R., Stormer, H. L., Tsui, D. C., Pfeiffer, L. N. & West, K. W. Experimental evidence for new particles in the fractional quantum Hall effect. *Phys. Rev. Lett.* **70**, 2944–2947 (1993). 10053693 10.1103/PhysRevLett.70.2944

[53] Manoharan, H. C., Shayegan, M. & Klepper, S. J. Signatures of a novel fermi liquid in a two-dimensional composite particle metal. *Phys. Rev. Lett.* **73**, 3270–3273 (1994). 10057334 10.1103/PhysRevLett.73.3270

[54] Du, R. R. et al. Drastic enhancement of composite fermion mass near Landau level filling *v* = 1/2. *Phys. Rev. Lett.* **73**, 3274–3277 (1994). 10057335 10.1103/PhysRevLett.73.3274

[55] Zeng, Y. et al. High-quality magnetotransport in graphene using the edge-free corbino geometry. *Phys. Rev. Lett*. **122**, 137701 (2019). 10.1103/PhysRevLett.122.137701 31012609

[56] Ribeiro-Palau, R. et al. High-quality electrostatically defined Hall bars in monolayer graphene. *Nano Lett.* **19**, 2583–2587 (2019). 30839210 10.1021/acs.nanolett.9b00351

[57] Halperin, B. I., Lee, P. A. & Read, N. Theory of the half-filled Landau level. *Phys. Rev. B* **47**, 7312–7343 (1993). 10.1103/physrevb.47.7312 10004728

[58] Dutta, B. et al. Distinguishing between non-abelian topological orders in a quantum Hall system. *Science* **375**, 193–197 (2022). 34941364 10.1126/science.abg6116

[59] Dean, C. R. et al. Hofstadter’s butterfly and the fractal quantum Hall effect in moiré superlattices. *Nature* **497**, 598–602 (2013). 23676673 10.1038/nature12186

