# Supplementary material for: Cascade of even-denominator fractional quantum Hall states in mixed-stacked multilayer graphene
Source: Nat Commun. 2026 May 13;17:6380. doi: 10.1038/s41467-026-73155-4 (PMC13376915; doi:10.1038/s41467-026-73155-4)
Supplement: Supplementary file 1 — Supplementary Information [file 41467_2026_73155_MOESM1_ESM.pdf]

Supplementary Information for

# Cascade of Even-Denominator Fractional Quantum Hall States in Mixed-Stacked Multilayer Graphene

Yating Sha[1†], Kai Liu[1†], Chenxin Jiang[2,3,4†], Dan Ye[2,3,5], Shuhan Liu[1], Zhongxun Guo[6], Jingjing Gao[6], Ming Tian[6,7], Neng Wan[6,7], Kenji Watanabe[8], Takashi Taniguchi[9], Bingbing Tong[10,11], Guangtong Liu[10,11]*, Li Lu[10,11], Yuanbo Zhang[6], Zhiwen Shi[1], Zixiang Hu[2,3]*, Guorui Chen[1]*

[1]State Key Laboratory of Micro-nano Engineering Science, Key Laboratory of Artificial Structures and Quantum Control (Ministry of Education), Tsung-Dao Lee Institute and School of Physics and Astronomy, Shanghai Jiao Tong University, Shanghai, China

[2]School of Physics, Chongqing University, Chongqing 401331, China

[3]Chongqing Key Laboratory for Strongly Coupled Physics, Chongqing University, Chongqing 401331, China

[4]Division of Physics and Applied Physics, Nanyang Technological University, Singapore 637371

[5]School of Teacher Development, Chongqing University of Education, Chongqing 400065, China

[6]State Key Laboratory of Surface Physics and Department of Physics, Fudan University, Shanghai 200433, China

[7]Key Laboratory of MEMS of Ministry of Education, School of Integrated Circuits, Southeast University, Jiangsu, Nanjing 210096, China

[8]Research Center for Electronic and Optical Materials, National Institute for Materials Science, 1-1 Namiki, Tsukuba, Japan

[9]Research Center for Materials Nanoarchitectonics, National Institute for Materials Science, 1-1 Namiki, Tsukuba, Japan

[10]Beijing National Laboratory for Condensed Matter Physics, and Institute of Physics, Chinese Academy of Sciences, Beijing 100190, China

[11]Hefei National Laboratory, Hefei, Anhui 230088, China

[†]These authors contributed equally to this work.

*Correspondence to: chenguorui@sjtu.edu.cn, zxhu@cqu.edu.cn, gtliu@iphy.ac.cn

# Supplementary Notes

## Model

In pentalayer graphene, we denote the layer index as $1,2,3,4,5$ and label the sublattice of each layer A and B. The atomic distance is $d_0 = 0.142$ nm, and the lattice constant is $a = \sqrt{3}d_0 = 0.246$nm. Choosing the basis $(A_1, B_1, A_2, B_2, A_3, B_3, A_4, B_4, A_5, B_5)$, the tight-binding Hamiltonian for ABCBC-stacked pentalayer graphene can be written as

$$\hat{H}_{ABCBC} = \begin{pmatrix} -2U+\delta_1 & -\gamma_0 f(\vec{k}) & -\gamma_4 f(\vec{k}) & -\gamma_3 f^*(\vec{k}) & \frac{\gamma_2}{2} & 0 & 0 & 0 & 0 & 0 \\ -\gamma_0 f^*(\vec{k}) & -2U+\delta_2 & \gamma_1 & -\gamma_4 f(\vec{k}) & 0 & \frac{\gamma_5}{2} & 0 & 0 & 0 & 0 \\ -\gamma_4 f^*(\vec{k}) & \gamma_1 & -U+\delta_3 & -\gamma_0 f(\vec{k}) & -\gamma_4 f^*(\vec{k}) & \gamma_1 & \frac{\gamma_5}{2} & 0 & 0 & 0 \\ -\gamma_3 f(\vec{k}) & -\gamma_4 f^*(\vec{k}) & -\gamma_0 f^*(\vec{k}) & -U+\delta_4 & -\gamma_3 f(\vec{k}) & -\gamma_4 f^*(\vec{k}) & 0 & \frac{\gamma_2}{2} & 0 & 0 \\ \frac{\gamma_2}{2} & 0 & -\gamma_4 f(\vec{k}) & -\gamma_3 f^*(\vec{k}) & \delta_5 & -\gamma_0 f(\vec{k}) & -\gamma_4 f(\vec{k}) & -\gamma_3 f^*(\vec{k}) & 0 & \frac{\gamma_2}{2} \\ 0 & \frac{\gamma_5}{2} & \gamma_1 & -\gamma_4 f(\vec{k}) & -\gamma_0 f^*(\vec{k}) & \delta_6 & \gamma_1 & -\gamma_4 f(\vec{k}) & 0 & 0 \\ 0 & 0 & \frac{\gamma_5}{2} & 0 & -\gamma_4 f^*(\vec{k}) & \gamma_1 & U+\delta_7 & -\gamma_0 f(\vec{k}) & -\gamma_4 f(\vec{k}) & -\gamma_3 f^*(\vec{k}) \\ 0 & 0 & 0 & \frac{\gamma_2}{2} & -\gamma_3 f(\vec{k}) & -\gamma_4 f^*(\vec{k}) & -\gamma_0 f(\vec{k}) & U+\delta_8 & \gamma_1 & -\gamma_4 f(\vec{k}) \\ 0 & 0 & 0 & 0 & 0 & 0 & -\gamma_4 f^*(\vec{k}) & \gamma_1 & 2U+\delta_9 & -\gamma_0 f(\vec{k}) \\ 0 & 0 & 0 & 0 & \frac{\gamma_2}{2} & 0 & -\gamma_3 f(\vec{k}) & -\gamma_4 f^*(\vec{k}) & -\gamma_0 f^*(\vec{k}) & 2U+\delta_{10} \end{pmatrix}, \quad (1)$$

where $\gamma_i$ is the hopping constant considered in the Slonczewski-Weiss-McClure (SWMC) parametrization, and $\delta_1, \dots, \delta_{10}$ are the onsite potentials. We introduce a tunable perpendicular electric field to control the interlayer potential difference $U$. For convenience, we assume equal-magnitude potential drops across the layers, proportional to $U$. We adopt the parameters $\gamma_0 = -3$ eV, $\gamma_1 = 0.39$ eV, $\gamma_2 = -0.017$ eV, $\gamma_3 = 0.3$ eV, $\gamma_4 = 0.066$ eV, $\gamma_5 = 0.038$ eV, $\delta_1 = \delta_{10} = 0.018$ eV, $\delta_2 = \delta_9 = 0.043$ eV, $\delta_3 = 0.05$ eV, $\delta_4 = \delta_5 = 0$, $\delta_6 = 0.05$ eV and $\delta_7 = \delta_8 = 0.025$ eV.

The tight-binding model for ABCBC-stacked pentalayer graphene in a perpendicular magnetic field $B$ generated by the vector $\vec{A}$ can be expanded around the Dirac point $\vec{K}_\pm = (\pm 4\pi/3a, 0)$ as follows

$$\hat{H}^{K_+}_{ABCBC} = \begin{pmatrix} -2U+\delta_1 & -v_0\Pi & -v_4\Pi & -v_3\Pi^\dagger & \frac{\gamma_2}{2} & 0 & 0 & 0 & 0 & 0 \\ -v_0\Pi^\dagger & -2U+\delta_2 & \gamma_1 & -v_4\Pi & 0 & \frac{\gamma_5}{2} & 0 & 0 & 0 & 0 \\ -v_4\Pi^\dagger & \gamma_1 & -U+\delta_3 & -v_0\Pi & -v_4\Pi^\dagger & \gamma_1 & \frac{\gamma_5}{2} & 0 & 0 & 0 \\ -v_3\Pi & -v_4\Pi^\dagger & -v_0\Pi^\dagger & -U+\delta_4 & -v_3\Pi & -v_4\Pi^\dagger & 0 & \frac{\gamma_2}{2} & 0 & 0 \\ \frac{\gamma_2}{2} & 0 & -v_4\Pi & -v_3\Pi^\dagger & \delta_5 & -v_0\Pi & -v_4\Pi & -v_3\Pi^\dagger & 0 & \frac{\gamma_2}{2} \\ 0 & \frac{\gamma_5}{2} & \gamma_1 & -v_4\Pi & -v_0\Pi^\dagger & \delta_6 & \gamma_1 & -v_4\Pi & 0 & 0 \\ 0 & 0 & \frac{\gamma_5}{2} & 0 & -v_4\Pi^\dagger & \gamma_1 & U+\delta_7 & -v_0\Pi & -v_4\Pi & -v_3\Pi^\dagger \\ 0 & 0 & 0 & \frac{\gamma_2}{2} & -v_3\Pi & -v_4\Pi^\dagger & -v_0\Pi & U+\delta_8 & \gamma_1 & -v_4\Pi \\ 0 & 0 & 0 & 0 & 0 & 0 & -v_4\Pi^\dagger & \gamma_1 & 2U+\delta_9 & -v_0\Pi \\ 0 & 0 & 0 & 0 & \frac{\gamma_2}{2} & 0 & -v_3\Pi & -v_4\Pi^\dagger & -v_0\Pi^\dagger & 2U+\delta_{10} \end{pmatrix}, \quad (2)$$

$$
\hat{H}_{ABCBC}^{K-} = \begin{pmatrix}
-2U+\delta_1 & v_0\Pi^\dagger & v_4\Pi^\dagger & v_3\Pi & \frac{\gamma_2}{2} & 0 & 0 & 0 & 0 & 0 \\
v_0\Pi & -2U+\delta_2 & \gamma_1 & v_4\Pi^\dagger & 0 & \frac{\gamma_5}{2} & 0 & 0 & 0 & 0 \\
v_4\Pi & \gamma_1 & -U+\delta_3 & v_0\Pi^\dagger & v_4\Pi & \gamma_1 & \frac{\gamma_5}{2} & 0 & 0 & 0 \\
v_3\Pi^\dagger & v_4\Pi & v_0\Pi & -U+\delta_4 & v_3\Pi^\dagger & v_4\Pi & 0 & \frac{\gamma_2}{2} & 0 & 0 \\
\frac{\gamma_2}{2} & 0 & v_4\Pi^\dagger & v_3\Pi & \delta_5 & v_0\Pi^\dagger & v_4\Pi^\dagger & v_3\Pi & 0 & \frac{\gamma_2}{2} \\
0 & \frac{\gamma_5}{2} & \gamma_1 & v_4\Pi^\dagger & v_0\Pi & \delta_6 & \gamma_1 & v_4\Pi^\dagger & 0 & 0 \\
0 & 0 & \frac{\gamma_5}{2} & 0 & v_4\Pi & \gamma_1 & U+\delta_7 & v_0\Pi^\dagger & v_4\Pi^\dagger & v_3\Pi \\
0 & 0 & 0 & \frac{\gamma_2}{2} & v_3\Pi^\dagger & v_4\Pi & v_0\Pi^\dagger & U+\delta_8 & \gamma_1 & v_4\Pi^\dagger \\
0 & 0 & 0 & 0 & 0 & 0 & v_4\Pi & \gamma_1 & 2U+\delta_9 & v_0\Pi^\dagger \\
0 & 0 & 0 & 0 & \frac{\gamma_2}{2} & 0 & v_3\Pi^\dagger & v_4\Pi & v_0\Pi & 2U+\delta_{10}
\end{pmatrix}, \tag{3}
$$

where $\Pi = (p_x - eA_x) - \mathrm{i}(p_y - eA_y)$, $v_i = \sqrt{3}a\gamma_i/2\hbar$. The momentum operator can be written by the ladder operator of non-relativistic Landau level (NRLL) [1]

$$
v_i\Pi = \frac{\mathrm{i}\sqrt{3}a\gamma_i}{\sqrt{2}l_B}\hat{\mathbb{a}}, \qquad v_i\Pi^\dagger = -\frac{\mathrm{i}\sqrt{3}a\gamma_i}{\sqrt{2}l_B}\hat{\mathbb{a}}^\dagger \tag{4}
$$

where $l_B = \sqrt{\hbar/eB}$ is the magnetic length, $\hat{\mathbb{a}}|n\rangle = \sqrt{n}|n-1\rangle$ and $\hat{\mathbb{a}}^\dagger|n\rangle = \sqrt{n+1}|n+1\rangle$. $|n\rangle$ is the eigenstates of the number operator $\hat{\mathbb{a}}^\dagger\hat{\mathbb{a}}$, and $n$ is the index of the NRLL. Hereafter, the term "Landau level" (LL) refers exclusively to those in graphene, unless otherwise specified.

For the Hamiltonian Eq. (2) or Eq. (3), it is not easy to write down a simple form of the eigenstate $\Psi_{ABABC}$. Typically, each component can be expanded as a linear combination of the non-relativistic Landau levels (NRLLs), $\Psi_{ABCBC,k} = \sum_{j=0}^{\infty} \alpha_{k,j}\,|j\rangle$. To achieve a reasonably accurate approximation of the few energy bands near zero energy, a truncation of NRLLs at $j = \Lambda$ is necessary. Although this truncation introduces some unphysical eigenvalues, these states are primarily dominated by higher NRLL orbitals (close to $\Lambda$) [2]. Accordingly, we truncate the Landau level expansion at $\Lambda = 30$, allowing the eigenstate to be expressed as

$$
\Psi_{ABCBC} = \begin{pmatrix}
\alpha_{1,0}|0\rangle + \alpha_{1,1}|1\rangle + \alpha_{1,2}|2\rangle \cdots \alpha_{1,\Lambda}|\Lambda\rangle \\
\alpha_{2,0}|0\rangle + \alpha_{2,1}|1\rangle + \alpha_{2,2}|2\rangle \cdots \alpha_{2,\Lambda}|\Lambda\rangle \\
\alpha_{3,0}|0\rangle + \alpha_{3,1}|1\rangle + \alpha_{3,2}|2\rangle \cdots \alpha_{3,\Lambda}|\Lambda\rangle \\
\alpha_{4,0}|0\rangle + \alpha_{4,1}|1\rangle + \alpha_{4,2}|2\rangle \cdots \alpha_{4,\Lambda}|\Lambda\rangle \\
\alpha_{5,0}|0\rangle + \alpha_{5,1}|1\rangle + \alpha_{5,2}|2\rangle \cdots \alpha_{5,\Lambda}|\Lambda\rangle \\
\alpha_{6,0}|0\rangle + \alpha_{6,1}|1\rangle + \alpha_{6,2}|2\rangle \cdots \alpha_{6,\Lambda}|\Lambda\rangle \\
\alpha_{7,0}|0\rangle + \alpha_{7,1}|1\rangle + \alpha_{7,2}|2\rangle \cdots \alpha_{7,\Lambda}|\Lambda\rangle \\
\alpha_{8,0}|0\rangle + \alpha_{8,1}|1\rangle + \alpha_{8,2}|2\rangle \cdots \alpha_{8,\Lambda}|\Lambda\rangle \\
\alpha_{9,0}|0\rangle + \alpha_{9,1}|1\rangle + \alpha_{9,2}|2\rangle \cdots \alpha_{9,\Lambda}|\Lambda\rangle \\
\alpha_{10,0}|0\rangle + \alpha_{10,1}|1\rangle + \alpha_{10,2}|2\rangle \cdots \alpha_{10,\Lambda}|\Lambda\rangle
\end{pmatrix}. \tag{5}
$$

There are $10(\Lambda+1)$ unknown parameters. In the $|n\rangle$ basis, we can express the ladder operator by a $(\Lambda+1)\times(\Lambda+1)$ matrix as follows

$$
\hat{\mathbb{a}} = \begin{pmatrix}
0 & \sqrt{1} & 0 & 0 & \cdots \\
0 & 0 & \sqrt{2} & 0 & \cdots \\
0 & 0 & 0 & \sqrt{3} & \cdots \\
\cdots & \cdots & \cdots & \cdots & \cdots
\end{pmatrix}, \tag{6}
$$

$$\hat{\mathbb{a}}^\dagger = \begin{pmatrix} 0 & 0 & 0 & 0 & \cdots \\ \sqrt{1} & 0 & 0 & 0 & \cdots \\ 0 & \sqrt{2} & 0 & 0 & \cdots \\ \cdots & \cdots & \cdots & \cdots & \cdots \end{pmatrix}. \tag{7}$$

In this case, the Hamiltonian Eq. (2) or Eq. (3) become to be a $10(\Lambda+1)\times 10(\Lambda+1)$ matrix in the basis

$$\begin{pmatrix} |0\rangle_1 \\ |1\rangle_1 \\ \cdots \\ |\Lambda\rangle_1 \\ \cdots \\ |0\rangle_{10} \\ |1\rangle_{10} \\ \cdots \\ |\Lambda\rangle_{10} \end{pmatrix}. \tag{8}$$

In the case of many-electron systems, we take into account the screened Coulomb interaction

$$V(\vec{q}) = \frac{e^2}{4\pi\varepsilon_0\varepsilon_{\mathrm{BN}}^{\parallel}}\frac{2\pi}{q}\tanh(qd), \tag{9}$$

where $q = |\vec{q}|$, $\varepsilon_0$ is the vacuum permittivity, $d$ is the distance between the top and bottom graphite gates and $\varepsilon_{\mathrm{BN}}^{\parallel} = 6.6$ denotes the in-plane relative permittivity of hexagonal boron nitride (hBN) [3]. In the absence of interactions, the inclusion of electron spin leads to Zeeman splitting of the Landau levels (LLs). However, in the presence of Coulomb interactions, electrons often favor spin-polarized states [3,4]. Therefore, in our subsequent analysis, we neglect the spin degrees of freedom.

## Methods

Our calculations are performed on a rectangular torus geometry with dimensions $L_x$ and $L_y$, where $L_xL_y = 2\pi l_B^2 N_\phi$ and $N_\phi$ denotes the total number of magnetic flux quanta piercing the system. The torus geometry offers a unique advantage over other commonly used geometries in FQH studies, such as the disk or sphere, by exhibiting additional topological ground-state degeneracy. Notably, different FQH states exhibit distinct ground-state degeneracies, such as a sixfold ground-state degeneracy corresponding to the Moore-Read Pfaffian type state on the torus [5]. This makes the torus geometry a powerful diagnostic tool for distinguishing between different FQH phases, and serves as a key criterion in our analysis. In a non-relative system, the single-particle wavefunction with the Landau gauge $\vec{A} = (0, Bx)$ is

$$\psi_{n,m} = \frac{1}{\sqrt{2^n n!\sqrt{\pi} l_B L_y}}\sum_{j=-\infty}^{+\infty} e^{\mathrm{i}\frac{2\pi y}{L_y}(m+jN_\phi)}\, e^{-\frac{1}{2}\left[\frac{x}{l_B}-\frac{2\pi l_B}{L_y}(m+jN_\phi)\right]^2}\,\mathbb{H}_n\left[\frac{x}{l_B}-\frac{2\pi l_B}{L_y}\left(m+jN_\phi\right)\right], \tag{10}$$

where $n = 0, 1, 2, \ldots$ is the NRLL index, $m = 0, 1, \ldots, N_\phi - 1$ is the momentum, and $\mathbb{H}_n(x)$ is the Hermite polynomial. The Coulomb interaction Hamiltonian can be written as

$$\hat{H}_C = \frac{1}{2L_xL_y}\sum_{\vec{q}} V(\vec{q}) \sum_{\{n_i\}}\sum_{\{m_i\}} F_{n_1,n_3}(-\vec{q})F_{n_2,n_4}(\vec{q})e^{-\frac{q^2l_B^2}{2}}e^{\mathrm{i}\frac{q_xL_x(m_1-m_4)}{N_\phi}}$$
$$\cdot\,\delta'_{m_1+m_2,m_3+m_4}\delta'_{m_1-m_3,\frac{q_yL_y}{2\pi}}C^\dagger_{n_1,m_1}C^\dagger_{n_2,m_2}C_{n_4,m_4}C_{n_3,m_3}, \quad (11)$$

where $\delta'$ is a generalized Kronecker function and satisfies

$$\delta'_{s,t} = 1 \ \text{ if and only if } \ (s \bmod N_\phi) = (t \bmod N_\phi), \quad (12)$$

$F_{n_1,n_2}(\vec{q})$ is the form factor which can be written as [6]

$$F_{n_1,n_2}(\vec{q}) = \sqrt{\frac{\min(n_1,n_2)!}{\max(n_1,n_2)!}}\left[\frac{\mathrm{sgn}(n_1-n_2)q_y - \mathrm{i}q_x}{\sqrt{2}l_B}\right]^{|n_1-n_2|} L^{|n_1-n_2|}_{\min(n_1,n_2)}\left(\frac{q^2l_B^2}{2}\right), \quad (13)$$

where $\mathrm{sgn}(x)$ is the sign function and $L_n^m(x)$ is the Laguerre polynomial. $C^\dagger_{n,m}$ and $C_{n,m}$ denote the creation and annihilation operators for the $n$th NRLL (nNRLL), respectively. In pentalayer graphene, due to the nature of the single-particle wavefunctions (as given in Eq. (8)), the corresponding form factor $\tilde{F}_{l_1,l_2}$ can be expressed as a linear combination of those in non-relativistic systems

$$\tilde{F}_{l_1,l_2}(\vec{q}) = \sum_{i=1}^{10}\sum_{j=0}^{\Lambda}\sum_{j'=0}^{\Lambda} \alpha^{l_1*}_{i,j}\alpha^{l_2}_{i,j'}F_{j,j'}(\vec{q}) \ , \quad (14)$$

where $l_1$ and $l_2$ denote LLs of the pentalayer graphene. Thus, the Coulomb interaction Hamiltonian for pentalayer graphene is given by

$$\hat{H}_G = \frac{1}{2L_xL_y}\sum_{\vec{q}} V(\vec{q}) \sum_{\{l_i\}}\sum_{\{m_i\}} \tilde{F}_{l_1,l_3}(-\vec{q})\tilde{F}_{l_2,l_4}(\vec{q})e^{-\frac{q^2l_B^2}{2}}e^{\mathrm{i}\frac{q_xL_x(m_1-m_4)}{N_\phi}}$$
$$\cdot\,\delta'_{m_1+m_2,m_3+m_4}\delta'_{m_1-m_3,\frac{q_yL_y}{2\pi}}C^\dagger_{l_1,m_1}C^\dagger_{l_2,m_2}C_{l_4,m_4}C_{l_3,m_3}, \quad (15)$$

and the total Hamiltonian can be written as

$$\hat{H} = \sum_{l,m}\mathbb{E}^l C^\dagger_{l,m}C_{l,m} + \hat{H}_G, \quad (16)$$

where $\mathbb{E}^l$ denotes the $l$th energy of the $\hat{H}^{K\pm}_{ABCBC}$. Moreover, the pseudopotentials in five-layer graphene take into account the hybridization of Landau level orbitals and can be expressed as

$$V_m = \sum_{i_1=1}^{10}\sum_{i_2=1}^{10}\sum_{j_1,j_2,j_3,j_4} \alpha^*_{i_1,j_1}\alpha_{i_1,j_3}\alpha^*_{i_2,j_2}\alpha_{i_2,j_4}\delta_{m'+j_1+j_2,m+j_3+j_4}$$
$$\cdot\int\frac{d^2q}{(2\pi)^2}V(\vec{q})e^{-q^2}F_{j_1,j_3}(\vec{q})F_{j_2,j_4}(-\vec{q})F_{m,m'}(\sqrt{2}\vec{q}). \quad (17)$$

However, the energy spectrum alone is insufficient to unambiguously distinguish between certain FQH states, such as Moore-Read Pfaffian-type states. On genus-zero geometries like the sphere, the Pfaffian, anti-Pfaffian [7], and particle-hole symmetric Pfaffian (PH-Pfaffian) [8] states have distinct topological shifts [9], which allows for clear differentiation. In contrast, the torus geometry does not contain topological shift, making it much more difficult to distinguish between them using spectral properties alone. Therefore, to further investigate the properties of the system, we compute the chiral graviton spectral function [10] to identify the chirality of the collective excitation mode in the bulk which is another key feature to distinguish

these non-Abelian FQH states. The chiral graviton operator can be written as

$$\hat{O}_{\pm} = \frac{1}{2L_x L_y} \sum_{\vec{q}} \left(q_x \pm i q_y\right)^2 V(\vec{q}) \sum_{\{l_i\}} \sum_{\{m_i\}} \tilde{F}_{l_1,l_3}(-\vec{q}) \tilde{F}_{l_2,l_4}(\vec{q}) e^{-\frac{q^2 l_B^2}{2}} e^{i\frac{q_x L_x (m_1 - m_4)}{N_\Phi}}$$
$$\cdot \delta'_{m_1+m_2,m_3+m_4} \delta'_{m_1-m_3,\frac{q_y L_y}{2\pi}} C^{\dagger}_{l_1,m_1} C^{\dagger}_{l_2,m_2} C_{l_4,m_4} C_{l_3,m_3}, \quad (18)$$

Here, $\hat{O}_{\pm}$ respectively generate graviton modes with spin $\pm 2$. By computing the corresponding spectral functions (it can be calculated by Lanczos method [11])

$$I_{\pm}(\omega) = \sum_{n} \frac{\left|\langle \Phi_n | \hat{O}_{\pm} | \Phi_0 \rangle\right|^2}{W_{\pm}} \delta(\omega - \omega_n) , \quad (19)$$

Where $W_{\pm} = \langle \Phi_0 | \hat{O}_{\pm}^{\dagger} \hat{O}_{\pm} | \Phi_0 \rangle$ are the total weights, $|\Phi_n\rangle$ denotes the $n$th excited state of the Hamiltonian in Eq. (16), $|\Phi_0\rangle$ is the ground state and $\omega_n$ represents the gap between the excited state and ground state. By comparing the relative strengths of $I_+$ and $I_-$ under the same total weight, we can identify whether the FQH state is hole- or electron-like, thereby distinguishing the type of the Moore–Read state. Our findings indicate that the half-filled FQH states observed in experiments are likely to belong to two distinct topological phases: the Pfaffian ($\nu = -7/2$ and $-11/2$) and the anti-Pfaffian ($\nu = -5/2$, $-9/2$, and $-13/2$).

.

## Results

It is essential to clearly define the filling factor in pentalayer graphene at the outset of our analysis. Due to the complex LL crossings in pentalayer graphene, we begin by considering the case with zero perpendicular electric field, as shown in Supplementary Fig. 10a. In this case, we observe that as the magnetic field increases, there are no crossings near the charge neutrality point, allowing for a well-defined classification of particle-type and hole-type fillings, corresponding to the LLs with positive and negative filling factors, respectively.

For $U > 0$, as the perpendicular electric field is varied slightly, the energy of each band changes continuously. This continuity enables us to consistently distinguish between the positive filling factor's Landau levels (PFFLLs) and negative filling factor's Landau levels (NFFLLs), as illustrated in Supplementary Fig. 11b. In contrast, the situation for $U < 0$ is markedly different, as shown in Supplementary Fig. 11a. If one attempts to distinguish the PFFLLs and NFFLLs in the same manner, numerous band crossings emerge between them. As a result, it becomes difficult to clearly define the filling for $U < 0$. Therefore, in this work, we primarily focus on the regime with $U > 0$. Experimentally, we observe even-denominator fractional quantum Hall states in the field range of $15 - 18$ T, with particularly clear signatures at 18 T corresponding to the filling factors $-13/2$, $-11/2$, $-9/2$, $-7/2$, and $-5/2$.

To further understand this regime, we plot the LL diagram near the charge neutrality point at $B = 18$ T, highlighting PFFLLs and NFFLLs. At this field, the magnetic length is $l_B = 6.05$ nm, $d = 14.91\, l_B$, and the energy scale of the Coulomb interaction is given by $e^2/(4\pi\varepsilon_0\varepsilon_{\mathrm{BN}}^{\parallel} l_B) = 36.08$ meV. It indicates that the Coulomb interaction in this regime is relatively strong, necessitating the consideration of Landau level mixing (LLM) effects. The study of FQH states at half-integer fillings has long been a topic of intense interest. In non-relativistic systems, the most well-known example is the $\nu = 5/2$ state in the 1NRLL [12], which has been interpreted as a Moore-Read Pfaffian type non-Abelian state [13]. Motivated by this, we identify and mark with red hollow circles those LLs in the pentalayer graphene LL diagram that exhibit a significant weight (greater than 70%) in the 1NRLL component, as shown in Supplementary Fig. 11. Notably, two NFFLLs exhibit particularly high 1NRLL

weights, which explains the experimental observation of two series of half-integer fractional quantum Hall states, as shown in Fig. 1d of the main text. Furthermore, although it is very difficult to analytically decompose ABCBC-stacked graphene into well-defined bilayer AB and trilayer ABC stacking, especially under a finite perpendicular electric field, we can still estimate the contributions from each stacking type by computing the "occupation number". As shown in Supplementary Fig. 11d, these two NFFLLs are primarily composed of bilayer AB stacking components.

Due to the strong LLM effect, we consider the mixing of two adjacent relativistic LLs. This leads to two types of fillings: $1 + 1/2$ and $1/2$, as shown in Supplementary Fig. 12. The experimentally observed filling factors of interest can be expressed as: $\nu = -8 + 3/2, -6 + 1/2, -6 + 3/2, -4 + 1/2, -4 + 3/2$. As shown in Supplementary Fig.13, 15, 17, 19, 21, 23. All these cases exhibit six quasi-degenerate ground states in our numerical calculations, which is a characteristic feature of the Moore-Read Pfaffian type state. In the energy spectra presented, we plot the low-energy eigenstates as a function of the total momentum $Y$ within the range $[0, N_\phi/2)$ and the other half of the spectrum can be obtained by magnetic translation. All energy spectra reveal that LLM induces only minor modifications to the system's spectrum. Therefore, the essential physics at filling factors of $1/2$ type and $1 + 1/2$ type is primarily governed by the half-filled LL. This allows us to map the calculated fillings onto the two experimentally observed series of half-filled fractional quantum Hall states (Fig. 1d of the main text). Specifically, Supplementary Fig. 13–18 ($\nu = -5/2$, $-7/2$ and $-9/2$) correspond to the higher-energy NFFLLs marked in red (①) in Supplementary Fig. 11b, whereas fillings with larger absolute values correspond to the lower-energy red-labeled NFFLLs (②), as shown in Supplementary Fig.19–24 ($\nu = -9/2$, $-11/2$ and $-13/2$).

To determine the type of ground state, we compute the chiral graviton spectral functions at the aforementioned fillings, as shown in Supplementary Fig. 14, 16, 18, 20, 22, 24. To simplify the analysis, we project the chiral graviton operator onto a single LL. For $3/2$-type fillings, the operator is projected onto the higher LL, while for $1/2$-type fillings, it is projected onto the lower LL. In the absence of LLM, all states respect particle-hole symmetry. Once LLM is introduced, this symmetry is explicitly broken. By comparing the spectral responses, we find that all states exhibit only weak PH symmetry breaking. This could be attributed to finite-size effects inherent in the numerical calculation. Notably, however, the inclusion of Landau level mixing (LLM) leads to a shift in the total spectral weight toward a specific chirality. The states at $\nu = -5/2$, $-9/2$, and $-13/2$ tend to favor the anti-Pfaffian phase, which correspond to the $3/2$-type fillings. In contrast, the states at $1/2$-type fillings($\nu = -7/2$ and $-11/2$) exhibit characteristics more consistent with the Pfaffian phase. This suggests that the emergent chirality is filling-type dependent.

Another intriguing feature is that the fractional states −5/7, −9/5, −9/7, −12/7, −14/11, and −19/11 appear alongside the −9/2, −11/2, and −13/2 sequence, and only on one side of the sequence, while they are absent in the −5/2, −7/2 and -9/2 sequence. This behavior is closely related to the LL orbital character, which is directly reflected in the pseudopotential structure of the corresponding LLs. As shown in Supplementary Fig. 25, by comparing the pseudopotentials of the two LL orbitals involved, we find that the LL associated with larger absolute filling factors (labeled band ② in Supplementary Fig. 11b) exhibits a smaller $V_1$ but a larger $V_3$ over a certain range. This naturally favors the emergence of the 1/5 state and its corresponding hierarchy, because 1/5 state, compared to 1/3 state, is more sensitive to stronger $V_3$. Meanwhile, strong LL mixing breaks particle–hole symmetry, leading to the appearance of these states only on one side of the filling sequence. Furthermore, strong LL mixing can drive the formation of Wigner crystal–like phases, which may suppress or destabilize these states [14].

In addition, near $U = -25$ meV, we observe a cluster of three closely spaced LLs, as shown in Supplementary Fig. 10b. These three LLs are characterized by exceptionally large weight contributions from the 0NRLL, 1NRLL, and 2NRLL, respectively. In this regime, where strong LL mixing is present, the emergence of the Jain 221 state becomes possible. The Jain 221 state is a known exact zero-energy ground state of the short-range Trugman-Kivelson (TK)

interaction $\nabla^2\delta^{(2)}(\vec{r}_1 - \vec{r}_2)$ projected onto the combined Hilbert space of the 0NRLL, 1NRLL, and 2NRLL [15]. Furthermore, as shown in Supplementary Fig. 10a and 10b, decreasing the magnetic field leads to an increased number of Landau level crossings and a significant reduction in the energy gaps between different LLs. This results in strong LLM, involving additional Landau levels in which the 1NRLL component is very small or even entirely absent. Such enhanced mixing likely accounts for the absence of observable half-integer filling signals in experiments conducted at lower magnetic fields.

In summary, pentalayer graphene provides a rich platform for realizing diverse FQH states. In particular, a variety of non-Abelian Pfaffian type phases emerge in the hole-type filling regime, and this region may also host other exotic FQH states, including potentially the non-Abelian Jain 221 state. It is worth noting that in our calculations, spin degrees of freedom have been neglected. Incorporating spin could lead to an even richer landscape of FQH states, such as the Halperin 331 state [16]. This will be the subject of detailed investigation in our future work.

# Supplementary Figures

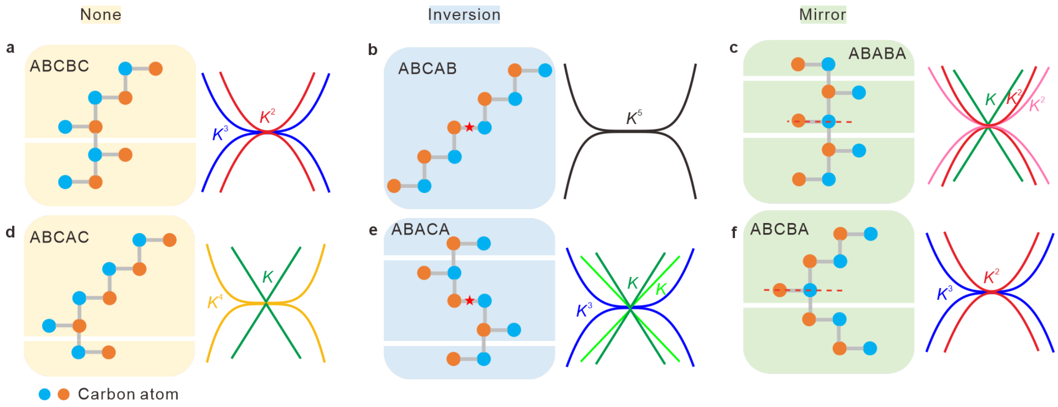


**Supplementary Figure 1: Possible stacking orders in pentalayer graphene.**

**a-f** Lattice and band structures of all six possible stacking orders of pentalayer graphene. Lattice structures with non-centrosymmetry, inversion symmetry and mirror symmetry are shaded in yellow, blue and green colors. Each lattice structure is decomposed into different blocks according to the chirality and the band structure can be simply obtained through the chiral decomposition.

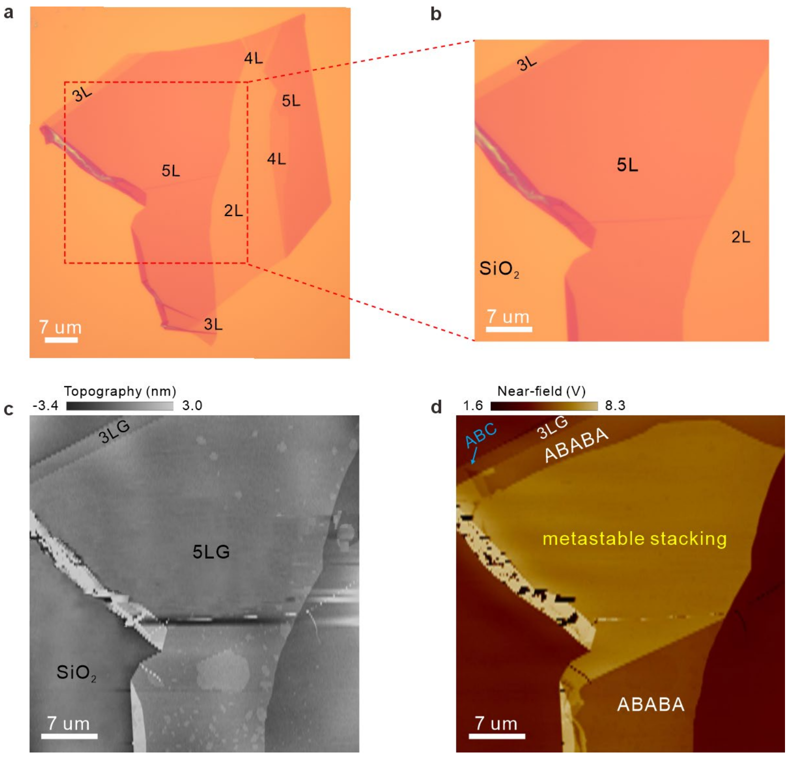


**Supplementary Figure 2: Imaging of ABCBC pentalayer graphene.**

**a** Optical image of the exfoliated graphene fleck on $SiO_2$/Si, where the layer number is identified by the reflection contrast between graphene and substrate using standard white-light microscopy (mainly the green channel). **b** Zoom-in of the pentalayer (5L) region. **c** Atomic force microscope (AFM) topography map of the same sample. **d** Corresponding near-field infrared image. The brightest area is tentatively identified as a metastable stacking (e.g., ABCBC), though the exact structure remains uncertain from this characterization alone. The surrounding regions appear to be Bernal stacking (ABABA). Notably, a dark domain in the trilayer region is clearly visible and can be reliably assigned to ABC stacking based on prior experience. This domain extends into the pentalayer region, exhibiting a third distinct contrast that indicates the presence of an additional metastable stacking order.

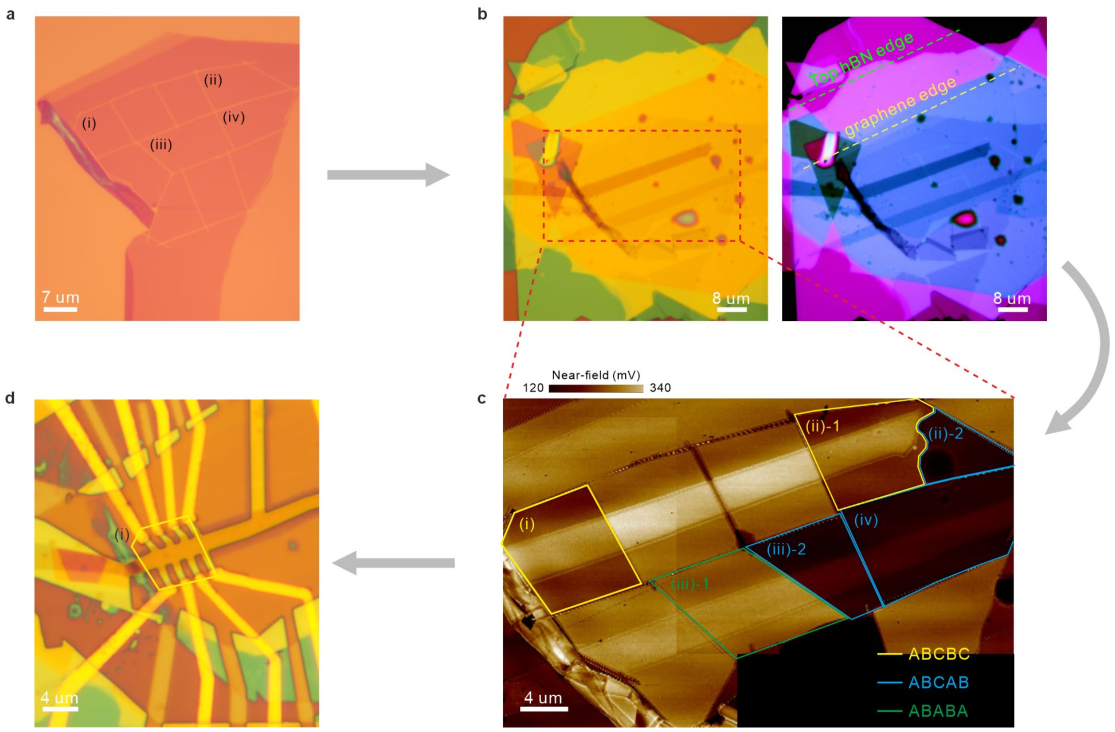


**Supplementary Figure 3: Fabrication of ABCBC pentalayer graphene device.**

**a** Optical image of pentalayer graphene after being cut into pieces using electrode-free anodic oxidation nanolithography. **b** Corresponding optical images after ABCBC is encapsulated with hBN on both sides and bottom graphite gate. The right-side picture is a high contrast version, for a better visibility. Green and yellow dashed lines are the edges of top hBN and graphene, they almost align with each other, suggesting the moiré is formed in the topmost graphene layer. **c** Identification of ABCBC in the hBN encapsulated structure using the phonon-polariton assisted scanning near-field microscopy. As shown in the image, ABCAB shows the darkest contrast (outlined in blue), ABCBC is in the middle contrast (yellow) and ABABA (green) is the brightest. The regions labeled with (i), (ii), (iii), (iv) are the same as those in **a**. Notably, after transferring, parts of (ii) and (iii) changed to another stacking, denoted as (ii-1/2) and (iii-1/2). Two bottom graphite gates also exhibit certain contrast inside the pentalayer graphene flakes. **d** Optical image of the final device (region (ii)) after the nanofabrication process such as EBL, RIE and e-beam evaporation.

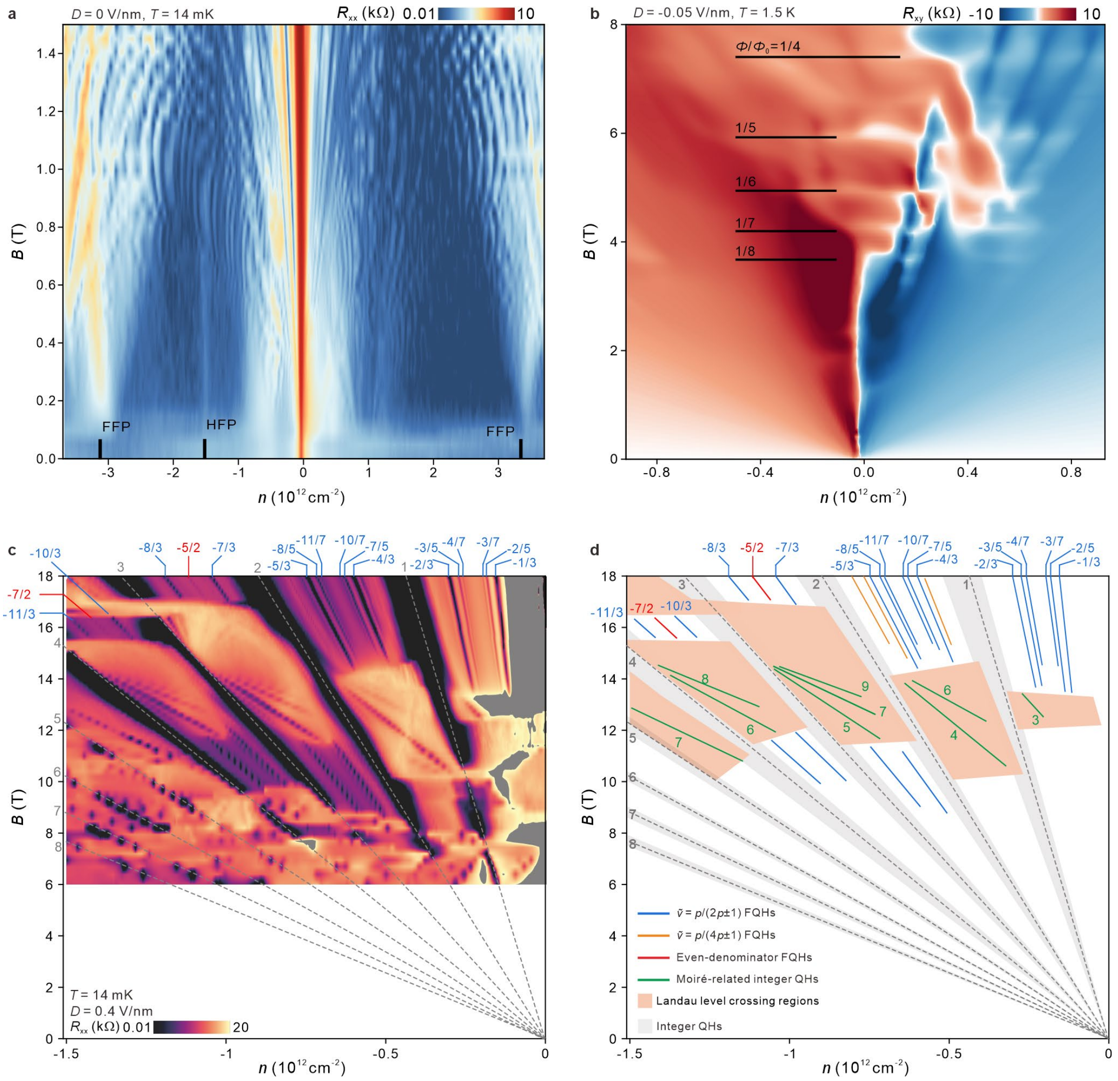


**Supplementary Figure 4: Landau fan diagram.**

**a** Low-field Landau fan diagram of $R_{xx}$ near $D = 0$. The full filling point (FFP) and half filling point (HFP) are marked, indicating the formation of a moiré superlattice between pentalayer graphene and hBN. **b** $R_{xy}$ - $n$ - $B$ color plot when $D$ = -0.05 V/nm, $T$ = 1.5 K. Brown–Zak oscillations are visible and highlighted by the black solid lines. From the periodicity of these oscillations, a moiré wavelength of 12.8 nm is extracted. **c** $R_{xx}$ - $n$ - $B$ color plot of ABCBC at $D$ = 0.4 V/nm when $T$ = 14 mK. **d** Corresponding phase diagram concluded from **c**, the integer, even-denominator fractional and moiré-related quantum Hall states are indicated by grey, red and green lines. The odd-denominator fractional quantum Hall states corresponding to the two-flux $CF_2$ sequence are marked in blue, with other states outside the $CF_2$ sequence shown in orange.

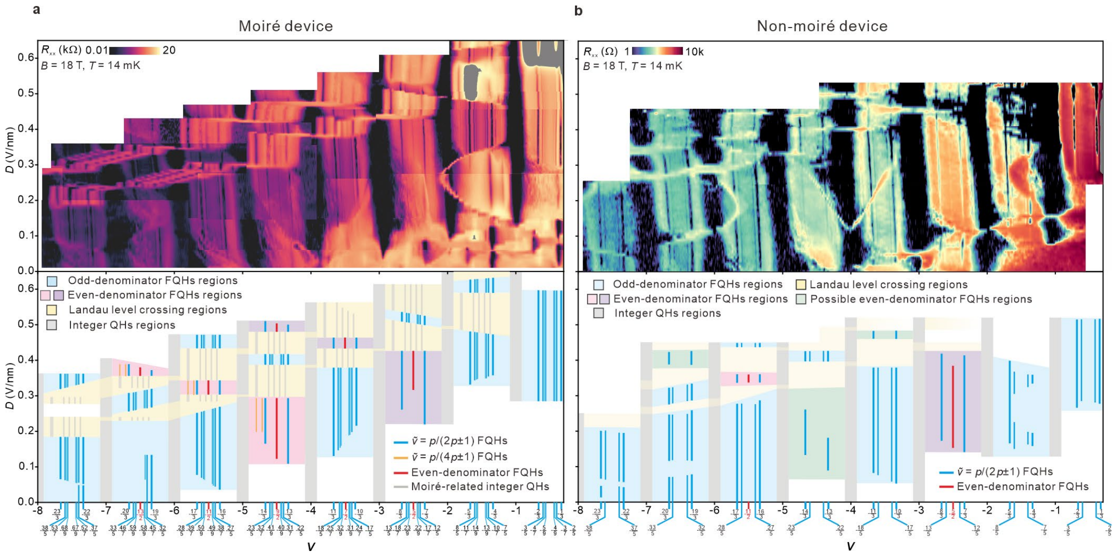


**Supplementary Figure 5: Comparison of phase diagram in ABCBC sample with (w/) and without (w/o) moiré.**

**a** Phase diagram of the sample S1 with moiré, reproduced from Fig. 4. **b** Phase diagram of another ABCBC-5LG sample S2 without moiré, closely resembling that in **a** Despite weaker $R_{xx}$ minima, a cascade of fractional quantum Hall features is still observed. Landau level crossings appear as increased resistance extending from the upper right to lower left, while no Bloch states are observed. We note that the suppression of adjacent Jain sequences suggests the emergence of half-filled states. These observations suggest that the effect of the moiré potential becomes prominent only when the Hofstadter gap is comparable to the Landau level gap near LL crossings.

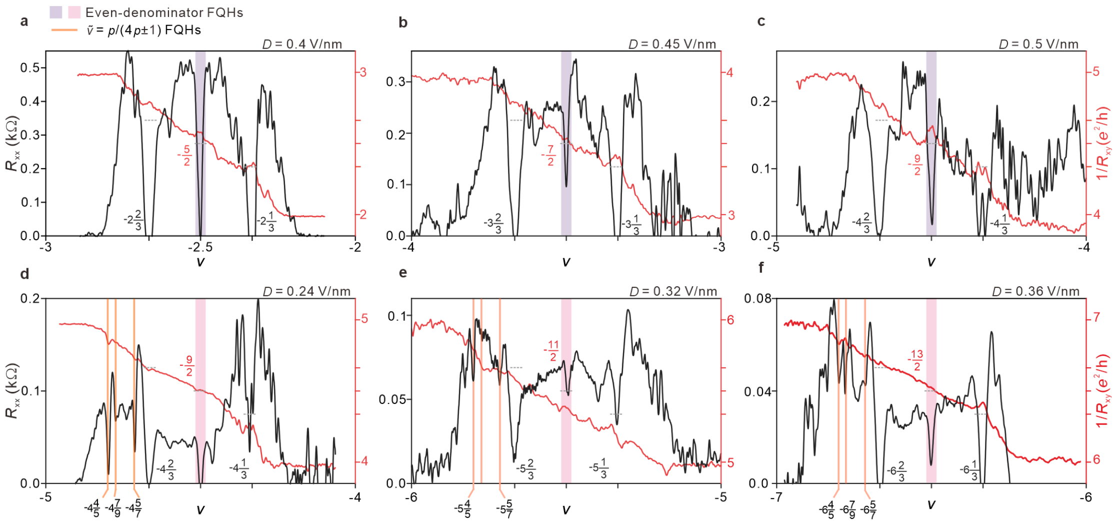

**Supplementary Figure 6: Even-denominator FQH states measured at *B* = -18 T.**

**a-f** Linecuts of $R_{xx}$ and $1/R_{xy}$ within the filling ranges of -3⩽ $\nu$ ⩽ -2 (**a**), -4 ⩽ $\nu$ ⩽ -3 (**b**), -5 ⩽ $\nu$ ⩽ -4 (**c** and **d**), -6 ⩽ $\nu$ ⩽ -5 (**e**) and -7 ⩽ $\nu$ ⩽ -6 (**f**), respectively. In comparison with the data at +18 T (see Fig. 3), the signatures of the two sets of half-filled states remain robust and reproducible, including the accompanying states beyond the $CF_2$ hierarchy in the second (pink shading) group. Remarkably, the $R_{xx}$ minima of the first state in each group, namely at -5/2 ($D$ = 0.4 V/nm) and -9/2 ($D$ = 0.24 V/nm), approach zero resistance in this case.

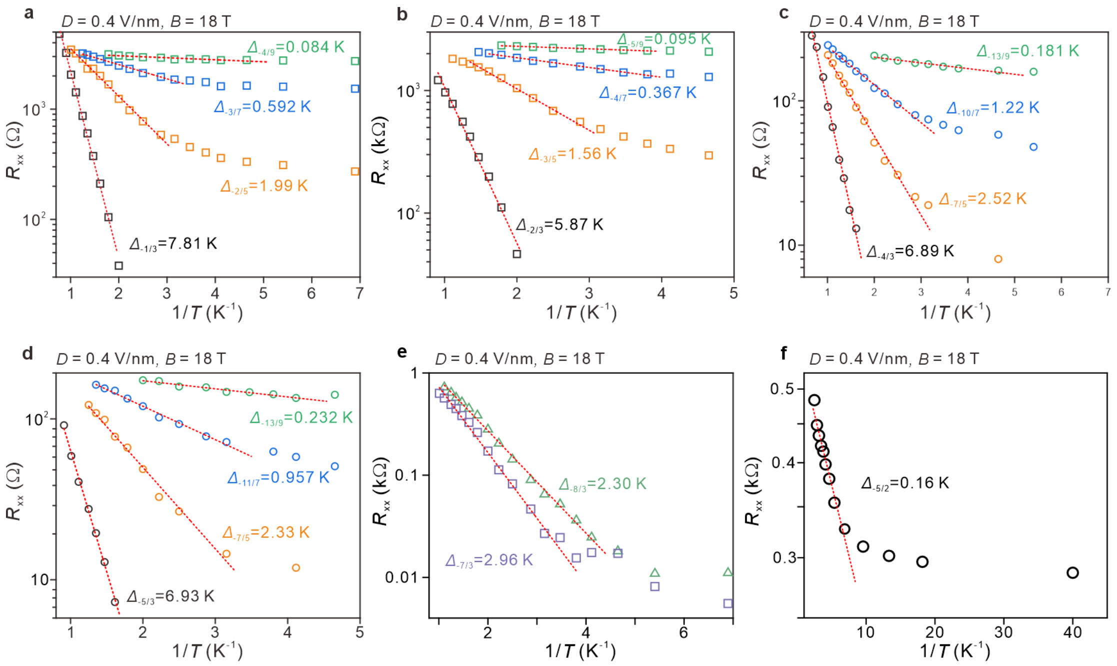


**Supplementary Figure 7: Thermal activation gaps in fractional quantum Hall states.**

**a-f** $R_{xx}$ versus $1/T$ plot of different fractional quantum Hall states when $D = 0.4$ V/nm and $B = 18$ T, the $R_{xx}$ axis is in the log scale, the labeled thermal activation gap is fitted using Arrhenius plot.

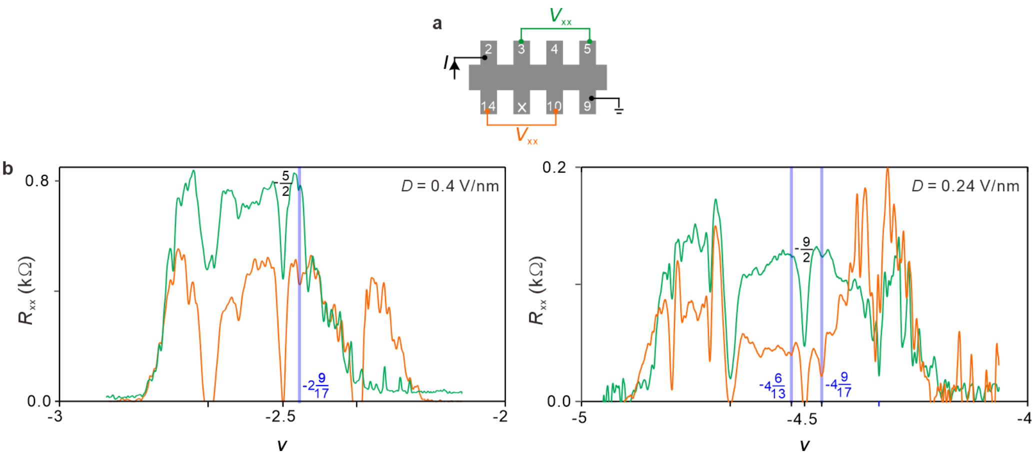


**Supplementary Figure 8: Particle-hole asymmetry at $\nu$ = -5/2 and -9/2.**

**a** Different measurement configurations on two edges. **b** $R_{xx}$ versus $\nu$ plot in the vicinity of $\nu$ = -5/2 and -9/2, respectively. Green and orange curves correspond to the two configurations in **a**. Weak but reproducible $R_{xx}$ minima are observed at $\tilde{\nu}$ = -6/13 and/or -9/17, marked by blue lines, which are the first daughter state of anti-Pfaffian state. Here, $\tilde{\nu}$ denotes $\nu + 2$ or $\nu + 4$.

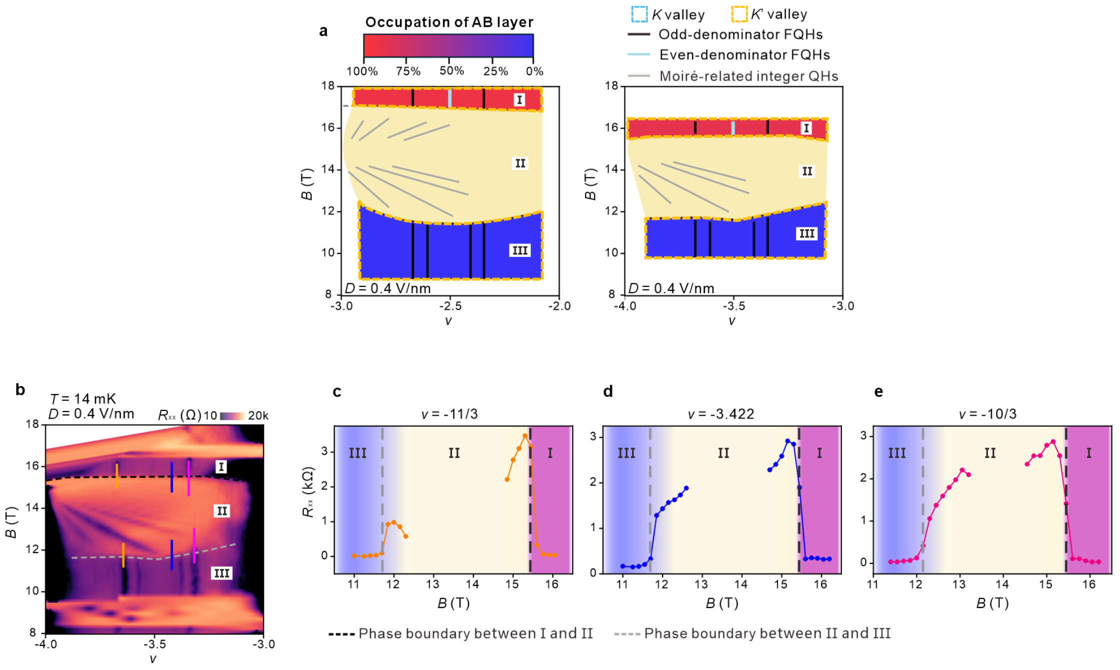


**Supplementary Figure 9: *B* dependent phase transitions around *ν* = -7/2.**

**a** Schematic phase diagram around $\nu = -5/2$ and $-7/2$. Around $\nu = -5/2$, AB layer occupation in I (III) is 92.75% (0.93%). Around $\nu = -7/2$, AB layer occupation in I (III) is 91.48% (0.93%). **b** $R_{xx}$-$\nu$-$B$ color plots around $\nu = -7/2$. The black and grey dashed lines are used to mark the phase boundary from odd-denominator FQHs regions to Landau level crossing regions, and the Landau level crossing regions to even-denominator FQHs regions, respectively. **c-e** are different linecuts of $R_{xx}$ along the orange, blue and pink lines in **b**. The blue, yellow and pink-filled regions represent the LL exhibiting odd-denominator FQH (III), Hofstadter minibands (II) and LL exhibiting even-denominator FQH (I), respectively.

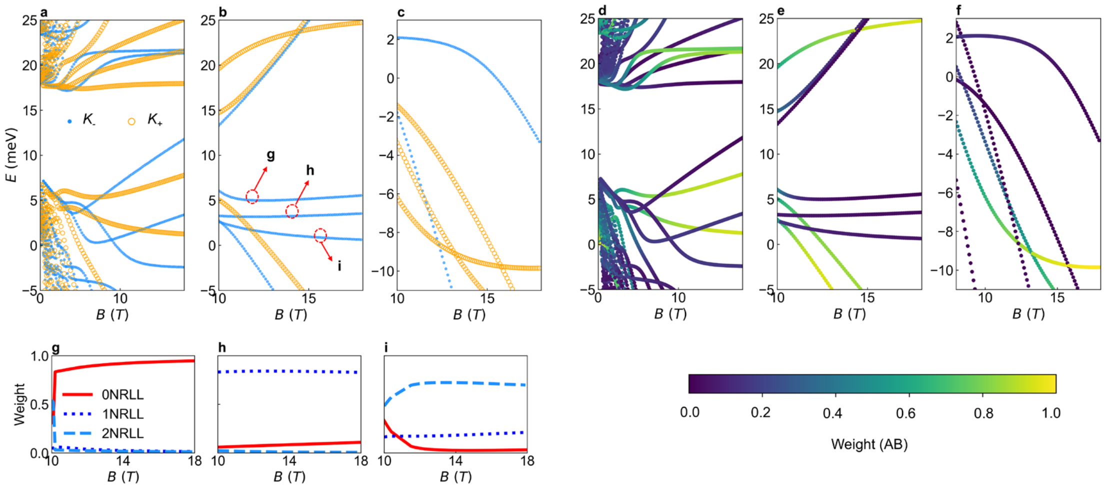


**Supplementary Figure 10: Band structure of the ABCBC-stacked pentalayer graphene**.

**a** LL diagram at $U = 0$, Blue dots denote Landau levels in the $K_-$ valley, while orange circles indicate those in the $K_+$ valley. **b** LL diagram at $U = -25$ meV. **c** LL diagram at $U = 18$ meV. **d** Contribution from the AB-stacked component to each LL at $U = 0$. **e** Contribution from the AB-stacked component to each LL at $U = -25$ meV. **f** Contribution from the AB-stacked component to each LL at $U = 18$ meV. **g**, **h**, **i** show the weights of the 0NRLL, 1NRLL, and 2NRLL components, respectively, for the LLs marked in red in panel **b**.

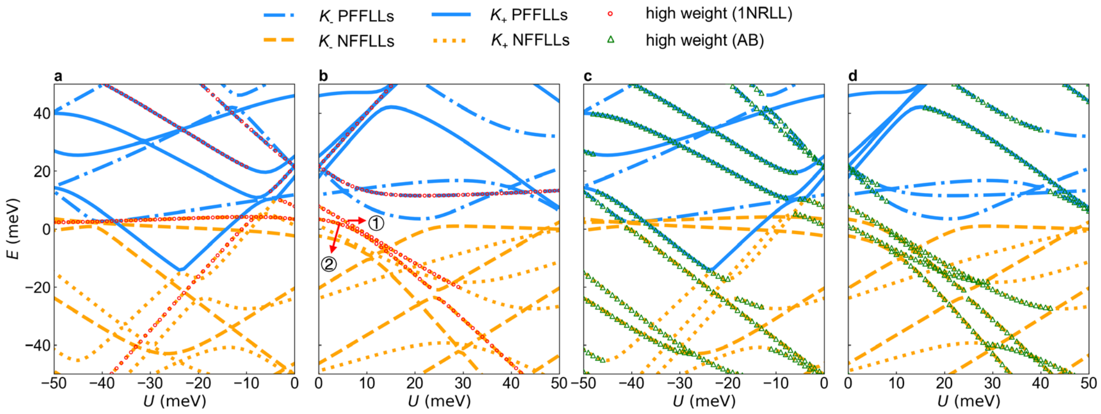


**Supplementary Figure 11: Energy bands of the ABCBC-stacked pentalayer graphene**.

The blue dashed lines denote positive filling factor's Landau level's (PFFLLs) in the $K_-$ valley, while the blue solid lines represent those in the $K_+$ valley. The orange dashed lines indicate negative filling factor's Landau level's (NFFLLs) in the $K_-$ valley, the orange dotted lines correspond to those in the $K_+$ valley. Landau levels marked with red circles possess a 1NRLL weight exceeding 70%, and the green hollow circles indicate states with more than 50% contribution from the bilayer AB-stacked configuration. **a** $U < 0$. **b** $U > 0$. **c** $U < 0$. **d** $U > 0$. Here, $U > 0$ ($U < 0$) corresponds to $D > 0$ ($D < 0$) in the experiment.

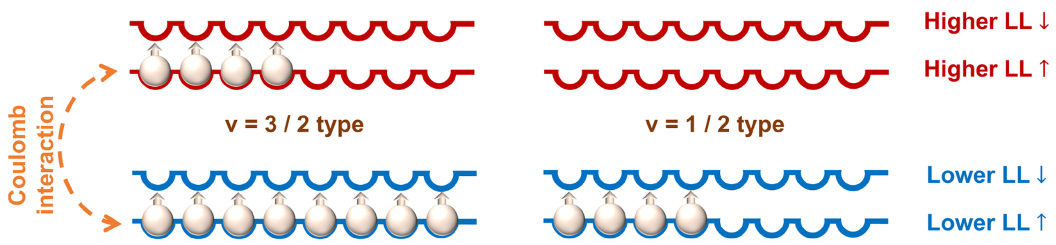


**Supplementary Figure 12: Schematic illustration of $3/2$-type and $1/2$-type fillings**.

Blue and red lines represent Landau levels (LLs), blue and red hollow circles denote unoccupied orbitals, and the sphere denotes an electron, with the arrow indicating its spin.

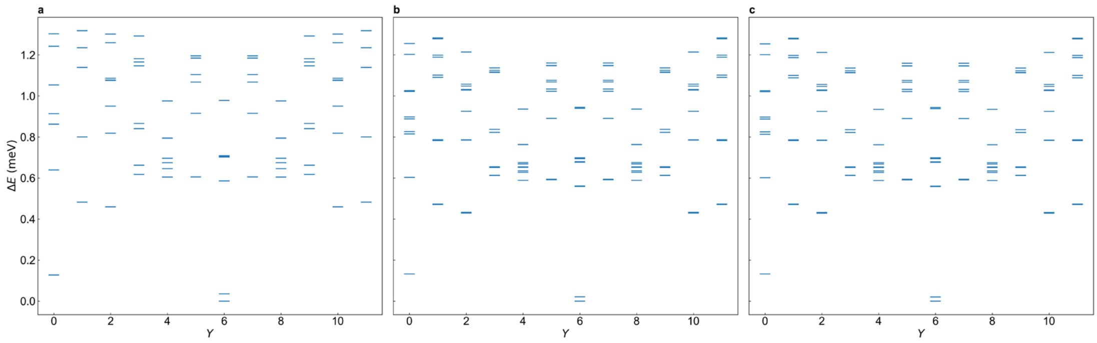


**Supplementary Figure 13: Low-energy of the spectra**.

$\nu = -5/2$ and $U = 6$ meV. $M_c$ denotes the number of electrons that is allowed to escape from the LL to the other LL and $\Delta E$ is defined as the energy relative to the ground state. **a** $M_c = 0$. **b** $M_c = 1$. **c** $M_c = 2$.

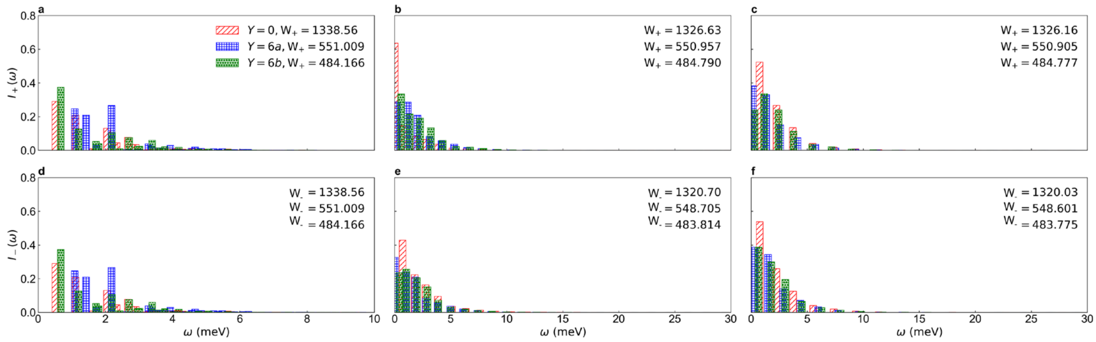


**Supplementary Figure 14: Chiral graviton spectral function**.

$\nu = -5/2$ and $U = 6$ meV. **a** $M_c = 0$. **b** $M_c = 1$. **c** $M_c = 2$. **d** $M_c = 0$. **e** $M_c = 1$. **f** $M_c = 2$.

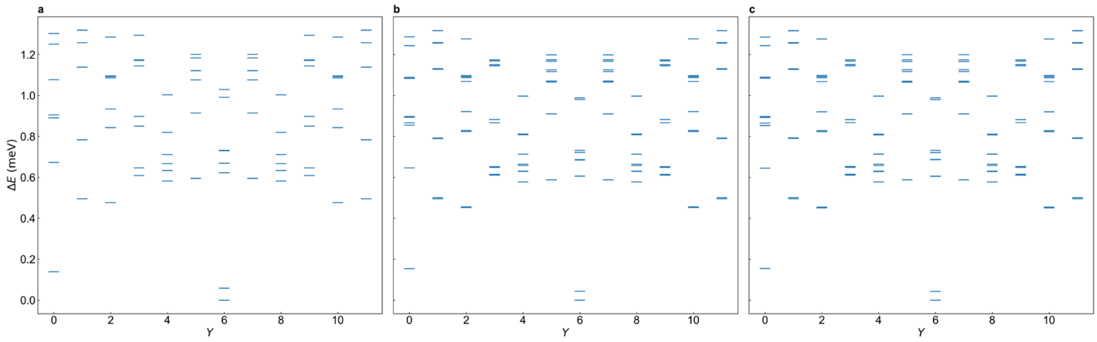


**Supplementary Figure 15: Low-energy of the spectra**.

$\nu = -7/2$ and $U = 19$ meV. **a** $M_c = 0$. **b** $M_c = 1$. **c** $M_c = 2$.

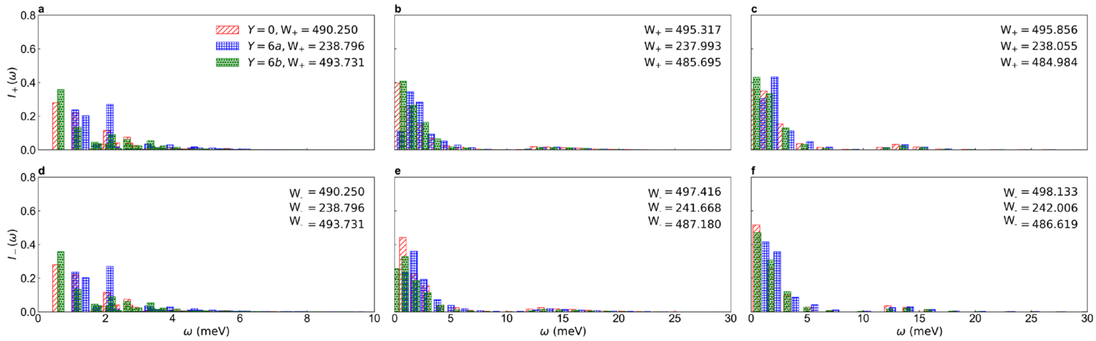


**Supplementary Figure 16: Chiral graviton spectral function**.

$\nu = -7/2$ and $U = 19$ meV. **a** $M_c = 0$. **b** $M_c = 1$. **c** $M_c = 2$. **d** $M_c = 0$. **e** $M_c = 1$. **f** $M_c = 2$.

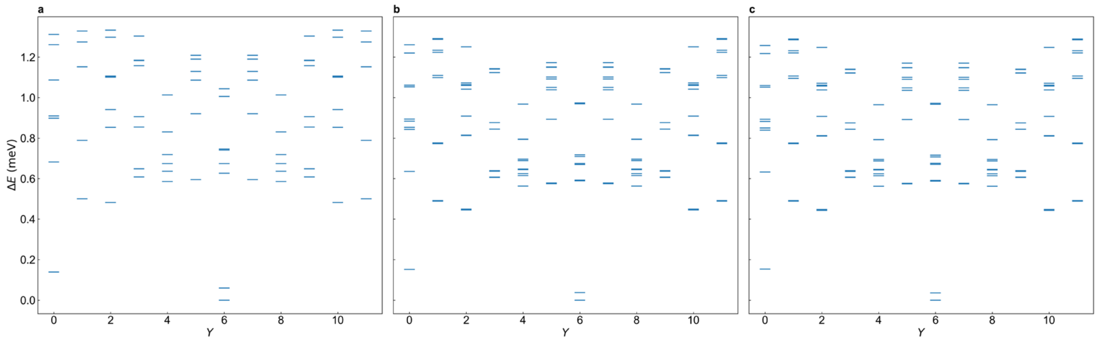


**Supplementary Figure 17: Low-energy of the spectra**.

$\nu = -9/2$ and $U = 18$ meV. **a** $M_c = 0$. **b** $M_c = 1$. **c** $M_c = 2$.

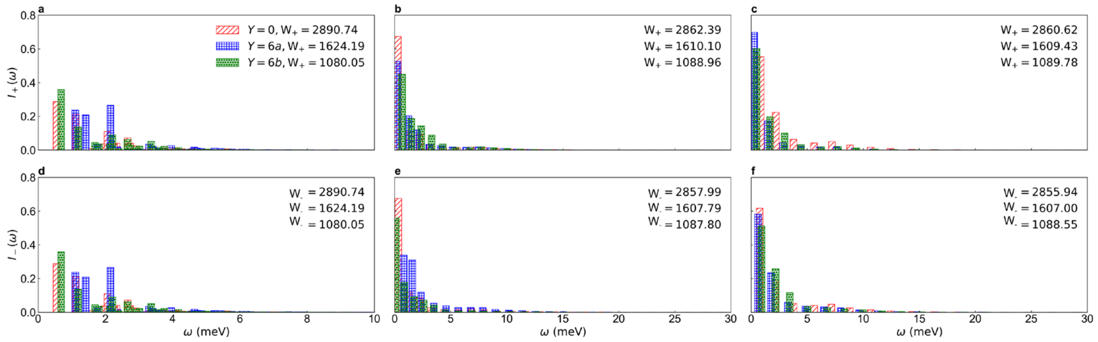


**Supplementary Figure 18: Chiral graviton spectral function**.

$\nu = -9/2$ and $U = 18$ meV. **a** $M_c = 0$. **b** $M_c = 1$. **c** $M_c = 2$. **d** $M_c = 0$. **e** $M_c = 1$. **f** $M_c = 2$.

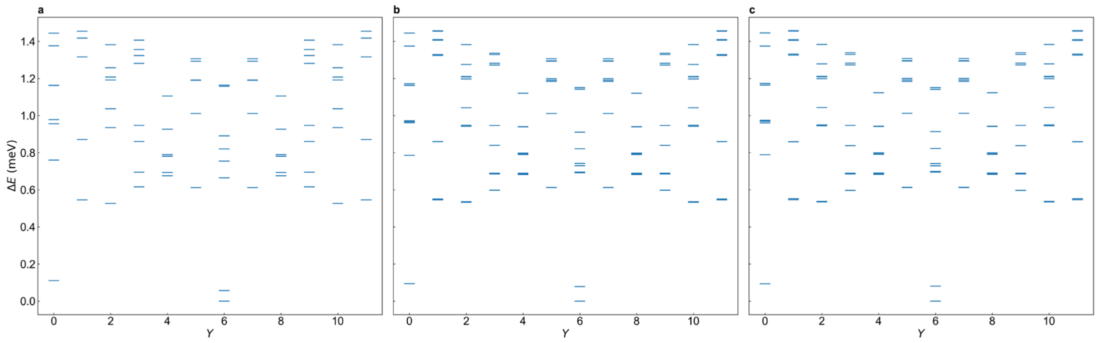

**Supplementary Figure 19: Low-energy of the spectra**.

$\nu = -11/2$ and $U = 18$ meV. **a** $M_c = 0$. **b** $M_c = 1$. **c** $M_c = 2$.

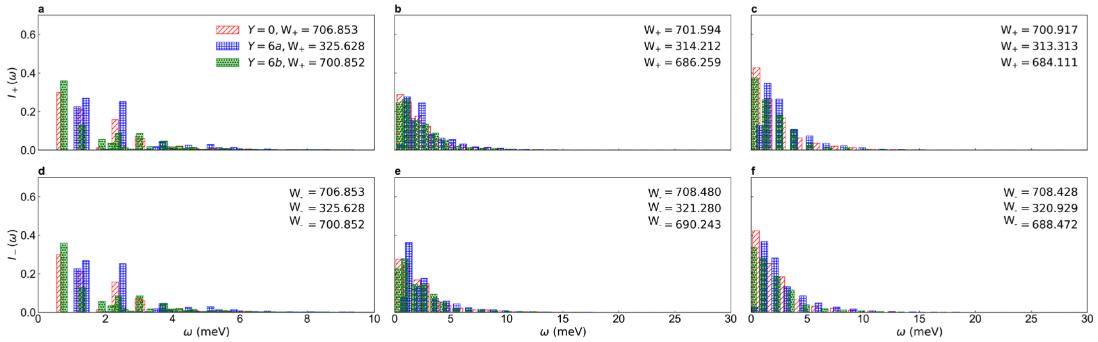


**Supplementary Figure 20: Chiral graviton spectral function**.

$\nu = -11/2$ and $U = 18$ meV. **a** $M_c = 0$. **b** $M_c = 1$. **c** $M_c = 2$. **d** $M_c = 0$. **e** $M_c = 1$. **f** $M_c = 2$.

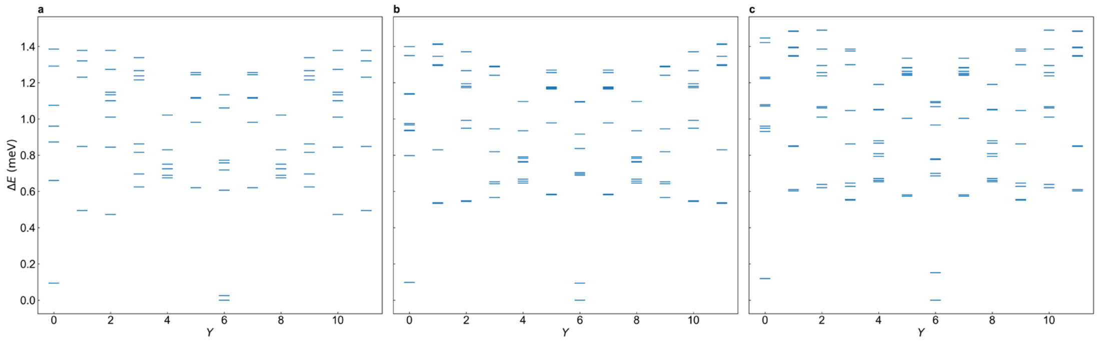


**Supplementary Figure 21: Low-energy of the spectra**.

$\nu = -13/2$ and $U = 16$ meV. **a** $M_c = 0$. **b** $M_c = 1$. **c** $M_c = 2$.

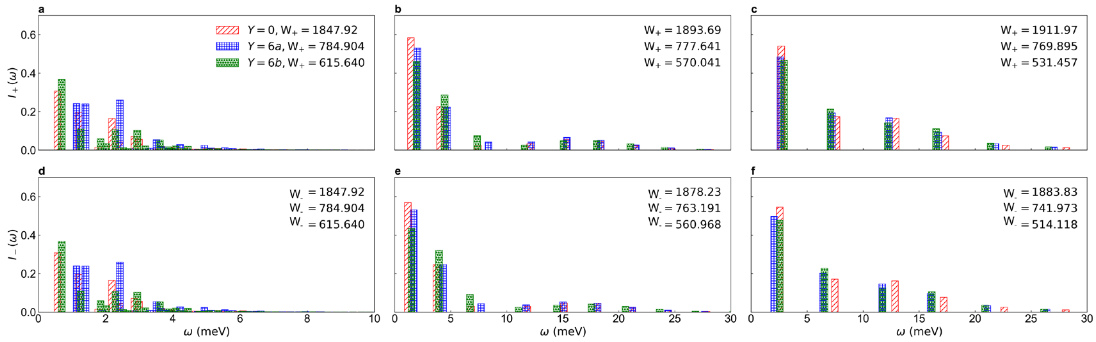


**Supplementary Figure 22: Chiral graviton spectral function.**

$\nu = -13/2$ and $U = 16$ meV. **a** $M_c = 0$. **b** $M_c = 1$. **c** $M_c = 2$. **d** $M_c = 0$. **e** $M_c = 1$. **f** $M_c = 2$.

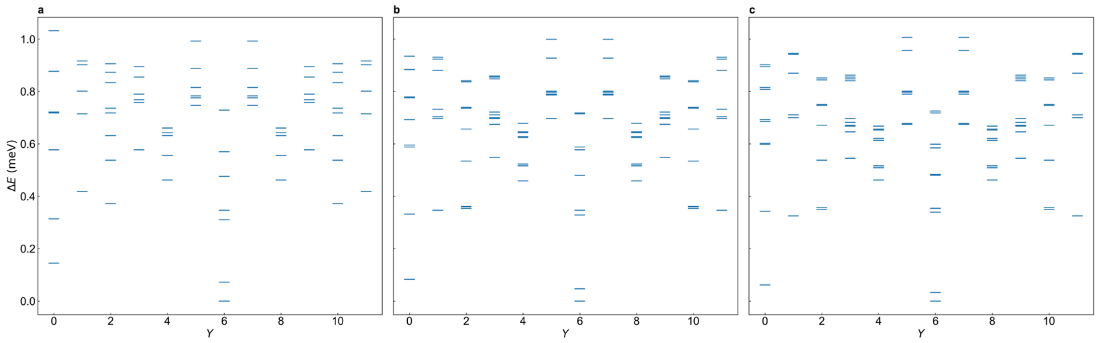

**Supplementary Figure 23: Low-energy of the spectra**.

$\nu = -9/2$ and $U = 5$ meV. **a** $M_c = 0$. **b** $M_c = 1$. **c** $M_c = 2$.

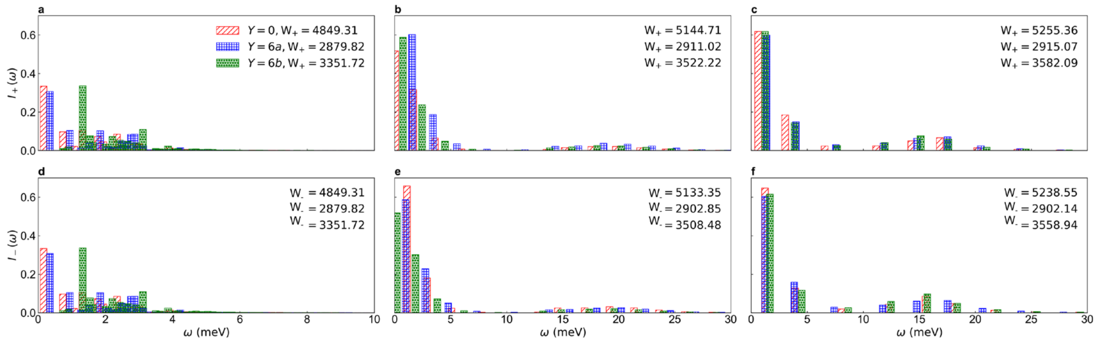

**Supplementary Figure 24: Chiral graviton spectral function.**

$\nu = -9/2$ and $U = 5$ meV. **a** $M_c = 0$. **b** $M_c = 1$. **c** $M_c = 2$. **d** $M_c = 0$. **e** $M_c = 1$. **f** $M_c = 2$.

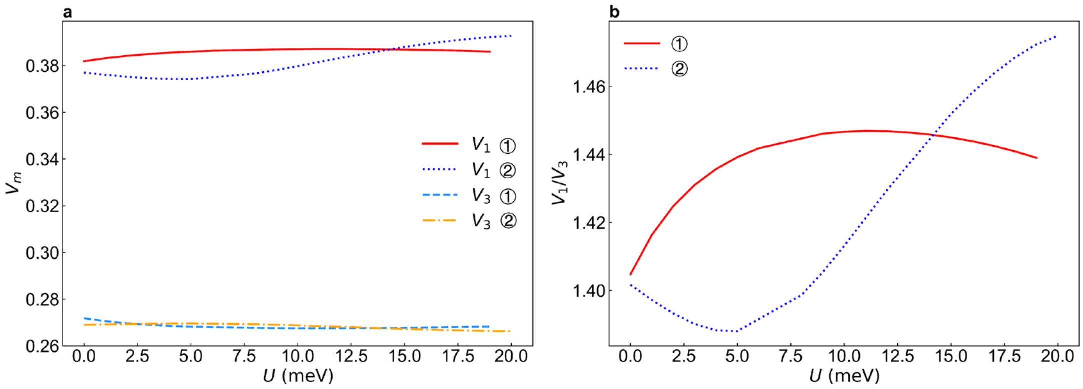


**Supplementary Figure 25: Pseudopotentials calculation.**

**a** Pseudopotentials of the two LL orbitals (labels ① and ② adopted from Supplementary Fig. 11b). **b** Ratio of $V_1/V_3$ for the two LL orbitals.

**Supplementary References**


1. Chakraborty, T. & Apalkov, V. Aspects of the fractional quantum Hall effect in graphene, in Physics of Graphene, edited by H. Aoki and M. S. Dresselhaus (*Springer International Publishing, Cham*, 2014) pp. 251–300.
2. Serbyn, M. & Abanin, D. A. New Dirac points and multiple Landau level crossings in biased trilayer graphene, *Phys. Rev. B* **87**, 115422 (2013).
3. Chen, Y. et al. Tunable even- and odd-denominator fractional quantum Hall states in trilayer graphene, *Nat. Comm.* **15**, 6236 (2024).
4. Hu, Z. X. et al. Realizing universal edge properties in graphene fractional quantum Hall liquids, *Phys. Rev. Lett.* **107**, 236806 (2011).
5. Zhu, W. et al. Fractional quantum Hall bilayers at half filling: tunneling-driven non-Abelian phase, *Phys. Rev. B* **94**, 245147 (2016).
6. An, J. et al. Fractional quantum Hall coexistence phases in higher Landau levels of graphene, *Phys. Rev. B* **111**, 045110 (2025).
7. Levin, M. & Halperin, B. I. & Rosenow, B. Particle-hole symmetry and the Pfaffian state, *Phys. Rev. Lett.* **99**, 236806 (2007).
8. Zucker, P. T. & Feldman, D. E. Stabilization of the particle-hole Pfaffian order by Landau-level mixing and impurities that break particle-hole symmetry, *Phys. Rev. Lett.* **117**, 096802 (2016).
9. Rezayi, E. H. & Pakrouski, K. & Haldane, F. D. M. Stability of the particle-hole Pfaffian state and the 5/2 fractional quantum Hall effect, *Phys. Rev. B* **104**, L081407 (2021).
10. Liou, S. F. et al. Chiral gravitons in fractional quantum Hall liquids, *Phys. Rev. Lett.* **123**, 146801 (2019).
11. Gagliano, E. R. & Balseiro, C. A. Dynamical properties of quantum many-body systems at zero temperature, *Phys. Rev. Lett.* **59**, 2999 (1987).
12. Willett, R. et al. Observation of an even-denominator quantum number in the fractional quantum hall effect, *Phys. Rev. Lett.* **59**, 1776–1779 (1987).
13. Moore, G. & Read, N. Nonabelions in the fractional quantum Hall effect, *Nuclear Physics B* **360**, 362-396 (1991).
14. Wang, C. et al. Competing many-body phases at small fillings in ultrahigh-quality GaAs two-dimensional hole systems: role of Landau level mixing, *Phys. Rev. B* **111**, 085429 (2025).

15. Wu, Y. H. & Shi, T. & Jain, J. K. Non-Abelian parton fractional quantum Hall effect in multilayer graphene, *Nano Letters* **17**, 4643 (2017).

16. Halperin, B. I. Theory of the quantized Hall conductance, *Helv. Phys. Acta* **56**, 75 (1983).
