# Supplementary material for: Cascade of even-denominator fractional quantum Hall states in mixed-stacked multilayer graphene
Source: Nat Commun. 2026 May 13;17:6380. doi: 10.1038/s41467-026-73155-4 (PMC13376915; doi:10.1038/s41467-026-73155-4)
Supplement: Supplementary file 2 — Transparent Peer Review file [file 41467_2026_73155_MOESM2_ESM.pdf]

nature portfolio

Peer Review File

Cascade of Even-Denominator Fractional Quantum Hall States in Mixed-Stacked Multilayer Graphene

Corresponding Author: Professor Guorui Chen

This file contains all reviewer reports in order by version, followed by all author rebuttals in order by version.

Attachments originally included by the reviewers as part of their assessment can be found at the end of this file.

Version 0:

Reviewer comments:

Reviewer #1

(Remarks to the Author)
The manuscript entitled “Cascade of Even-Denominator Fractional Quantum Hall States in Mixed- Stacked Multilayer Graphene” reports even-denominator states in the zeroth Landau level of a mixed-stacked pentalayer graphene – previously unexplored system. The measurements are carried out at 14mK temperature and 18T magnetic field. Data are clear and neatly presented. The use of mixed-stacked pentalayer graphene creates a hybrid Landau level (LL) structure by intertwining quadratic (from bernal stacking) and cubic (from ABC stacking) band dispersions. This gives rise to highly degenerate (20 fold) zero energy LL in spin, valley and N=0,1 (from bernal part) and N=0,1,2(from ABC part) orbital degeneracy. The transverse displacement field (D) and charge carrier density that are controlled by top and bottom gates, allow changing LL characters at either at fixed filling or fixed D. Using this tunability, authors report even-denominator states at -5/2, -7/2, -9/2, including high-filling such as -11/2 and -13/2 that are rare or absent in zeroth LL of monolayer or bilayer graphene, and thus expands the known landscape of FQH phenomena. Authors have performed numerical calculations and shown that the observed even-denominator states belongs to non-Abelian Moore-Read type ground states.

The work establishes mixed-stacked pentalayer graphene as another tunable system for realizing even-denominator states. Even-denominator states with higher gaps have been previously realized in other simpler graphene-based systems such as bernal stacked bilayer graphene and trilayer graphene also. Directly probing these even-denominator states to test their exchange statistics or topological order would be of great interest to the broader condensed matter community. However, the current manuscript does not clearly articulate its broader impact.

Comments:

1. Authors should include cautionary statement on non-Abelian nature of the observed even-denominator states since they have not been directly probed via methods such as thermal conductance or interference experiments.

2. It will be extremely important and useful if authors can provide a diagram of LL crossings as a function of D, for ZLL of this system. It might be important to know which N=1 orbital (bernal bilayer or ABC trilayer part) is responsible for even-denominator states.

3. For the ground states to be non-Abelian, the wavefunctions should be single component in both spin and valley quantum numbers. Have authors performed in-plane magnetic field and D-dependent measurements on the observed even-denominator states?

4. It seems the data in Fig. 1d is patched. Was it taken during different cooldowns?

5. In Fig. 1d, are there additional crossings at $\nu=-13/2$ (in pink shaded area) around $D\approx0.37$ V/nm?

6. What do the grey shaded areas in Fig. 1d represent at high D between $-1<\nu<0$ and $-2<\nu<-1$?

7. Did authors measure between fillings -8 to -10, as the ZLL spans fillings from -10 to +10? Have authors measured on

electron-side? It would be interesting to see if the LL crossings are symmetric across charge neutrality.

8. What do the grey lines in the schematic of Fig. 1d represent? They are not mentioned in the figure caption.

9. In Fig.3, except panel d, the Rxy plateau value for nu= - i - 1/3 (i being an integer) fillings is much lower than the expected Rxy, however Rxx is fully developed. Can authors comment on this?

10. Do authors understand why Jain states of four-flux attached composite fermions appears along with the appearance of -9/2,-11/2, and -13/2 (pink shaded areas in schematic of Fig.1d) while they are absent with -5/2 and -7/2 (purple shaded areas)? Is it related to the LL orbital index?

11. In the sample without moiré, Extended data Fig.5, the even-denominator states are barely visible. Is this due to poor bulk quality of the sample or moiré pattern has a role in stabilizing the even-denominator states in the sample with moiré? Authors should should investigate it more by measuring more samples without moiré.

12. In line 170, there seems to be a typo: the word “red” should be replaced with “pink”.

13. In line 164, there seems to be typo: the word “-2/9” should be replaced by “-9/2”.

Reviewer #2

(Remarks to the Author)
The paper deals with a multilayer graphene stack that exhibits some FQHE features, which have been observed experimentally. This work is a sequel to a previous paper by this group of authors, already published in Nature Communications (Nat. Commun. 15, 6236 (2024) [24]) focused on similar observations in a 3-layer graphene stack (now extended to a 5-layer stack). Both papers are similar, and the new one repeats the same experimental procedures applied earlier to multilayer graphene heterostructures.

Experimentally, both papers are of a very high quality, and the obtained data are presented with sufficient care. However, the theoretical (explanatory) part of both papers is confusing -- in the current paper, to the same extent as in the previous publication by the same group [24]. These authors ignore that the "single quasiparticle CF approach" to FQHE in multilayer Hall systems does not work, as has already been clarified in the literature, especially in detail for bilayer graphene and double-layer GaAs heterostructures. Therefore, in multilayer Hall systems, the CF model cannot be applied to explain the physics behind the odd FQHE features observed in 3- and 5-layered Hall systems.

The CF theory is an auxiliary quasiparticle-type model for the topological effect of multiloop cyclotron braid orbits responsible for FQHE. The CF model only partially aligns with the deeper, current understanding of FQHE -- CF model works exclusively in the lowest LL of GaAs and monolayer graphene (except for the so-called enigmatic states and even-denominator fractions) and fails completely in higher LLs and in multilayer Hall systems. The latter is the case of the experimental study presented in this submission.

This limitation of the CF model usage is completely ignored in the submission. Its authors suggest some artificial fluxes added to electrons, mixing them with “chiral gravitons”, “pairing of CFs”, “quasiparticle-type phase transitions” and other rather irrelevant concepts, in an attempt to find an interpretation (which is, however, impossible within the CF framework).

The interesting observation of the lifting of some FQHE features by the application of a vertical electric field (referred to as "tunability") is especially falsely interpreted in terms of “symmetry breaking” or crossing single-particle bands. This is a misinterpretation, likely partly induced by the experimental paper by Maher et al. [21], which was also originally incorrectly interpreted. In fact, the lifting (tuning) of the FQHE hierarchy by the application of vertical voltage can be understood via a simple topological effect: the blockade of interlayer hopping of carriers against the electrical field. This prevents the division of the loops of multiloop cyclotron braid orbits among the layers (thus reducing the hierarchy of a multilayer system to that of a monolayer system), as detailed in the quantum topological approach -- cf. doi:10.1038/s41598-017-09166-5, Supplementary Information. Note that the reasons for the even denominator filling fractions in the FQHE hierarchy are at least threefold: the pairing of carriers (mentioned in the submission), specific topological commensurability invariants even in the LLL (e.g., doi.org/10.1088/1361-648X/ad7b92), and the division of loops of a multiloop braid orbit among layers in multilayer Hall systems -- all three of which can compete energetically.

Therefore, the theoretical side of the submission must be revised because, in its current form, the paper is clearly incorrect and cannot be published. The same concern applies to the introductory and concluding paragraphs, in which similarly confusing interpretations are repeated. On the formal side of the paper, please clarify what is meant by the equal contribution of the three first authors with respect to the other authors (who are not marked).

Reviewer #3

(Remarks to the Author)

Version 1:

Reviewer comments:

Reviewer #1

(Remarks to the Author)
The authors addressed all of my questions in detail. They did not perform in-plane measurements, which is understandable given their instrumentation.

One final question concerns Fig. R1. It appears that the even-denominator states originate primarily from the AB region. In GaAs and bilayer graphene studies, even-denominator formation is typically observed mainly in orbital 1 rather than orbital 0. Is the situation similar here?

Reviewer #3

(Remarks to the Author)
Thank you to the authors for addressing the questions and revising the manuscript. I have no further comments. The responses clarify the points raised, and the manuscript is now clear and relevant to the community.

Version 2:

Reviewer comments:

Reviewer #1

(Remarks to the Author)
Thank you to the authors for clarifying the final point I had raised. I now find the manuscript clear and have no objections to its publication.

Open Access This Peer Review File is licensed under a Creative Commons Attribution 4.0 International License, which permits use, sharing, adaptation, distribution and reproduction in any medium or format, as long as you give appropriate credit to the original author(s) and the source, provide a link to the Creative Commons license, and indicate if changes were made.
In cases where reviewers are anonymous, credit should be given to 'Anonymous Referee' and the source.
The images or other third party material in this Peer Review File are included in the article’s Creative Commons license, unless indicated otherwise in a credit line to the material. If material is not included in the article’s Creative Commons license and your intended use is not permitted by statutory regulation or exceeds the permitted use, you will need to obtain permission directly from the copyright holder.
To view a copy of this license, visit https://creativecommons.org/licenses/by/4.0/

Reviewer #1 (Remarks to the Author):

The manuscript entitled "Cascade of Even-Denominator Fractional Quantum Hall States in Mixed- Stacked Multilayer Graphene" reports even-denominator states in the zeroth Landau level of a mixed-stacked pentalayer graphene – previously unexplored system. The measurements are carried out at 14mK temperature and 18T magnetic field. Data are clear and neatly presented. The use of mixed-stacked pentalayer graphene creates a hybrid Landau level (LL) structure by intertwining quadratic (from bernal stacking) and cubic (from ABC stacking) band dispersions. This gives rise to highly degenerate (20 fold) zero energy LL in spin, valley and N=0,1 (from bernal part) and N=0,1,2(from ABC part) orbital degeneracy. The transverse displacement field (D) and charge carrier density that are controlled by top and bottom gates, allow changing LL characters at either at fixed filling or fixed D. Using this tunability, authors report even-denominator states at -5/2, -7/2, -9/2, including high-filling such as -11/2 and -13/2 that are rare or absent in zeroth LL of monolayer or bilayer graphene, and thus expands the known landscape of FQH phenomena. Authors have performed numerical calculations and shown that the observed even-denominator states belongs to non-Abelian Moore-Read type ground states.

The work establishes mixed-stacked pentalayer graphene as another tunable system for realizing even-denominator states. Even-denominator states with higher gaps have been previously realized in other simpler graphene-based systems such as bernal stacked bilayer graphene and trilayer graphene also. Directly probing these even-denominator states to test their exchange statistics or topological order would be of great interest to the broader condensed matter community. However, the current manuscript does not clearly articulate its broader impact.

Reply: We thank the referee for valuing the scientific merits of our work as "expand the known landscape of FQH phenomena." We fully agree that directly probing the exchange statistics or topological order of the observed even-denominator states—such as through thermal Hall conductance or interferometry—would significantly strengthen the broader impact of this work. However, as these measurements are beyond the scope of the present study, we have revised the manuscript to adopt a more cautious tone and avoid overstating implications that are not experimentally demonstrated.

we have revised the manuscript to highlight the broad interest of current work lying in its tunable landau level crossings, hybridization and interactions between different bands and layers under electric or magnetic fields, showing more freedoms compared with mono- or bilayer graphene to govern the stability and character of fractional quantum Hall states.

Comments:

1. Authors should include cautionary statement on non-Abelian nature of the observed even-denominator states since they have not been directly probed via methods such as thermal conductance or interference experiments.

Reply: We thank the referee for this important suggestion. We fully agree that the non-Abelian nature of the observed even-denominator fractional quantum Hall (FQH) states cannot be conclusively established without direct experimental probes such as thermal conductance or interference measurements. In the revised manuscript, we have softened our wording and added explicit cautionary statements:

(1) In the Abstract, the opening sentence in line #28 now reads:
"The fractional quantum Hall effect (FQHE), particularly at half-filling of Landau levels, provides a unique window into topological phases that **may** host non-Abelian excitations."

(2) In the Abstract, the sentence describing our numerical analysis in line #42 now reads:
"Numerical calculations, revealing characteristic six-fold ground-state degeneracy and chiral graviton spectral analysis, **suggest** that the observed even-denominator FQH states **may belong** to non-Abelian Moore-Read type, although direct probes of their exchange statistics remain an important goal for future experiments."

(3) In the main text, the third sentence of the fourth paragraph (original line #99), we delete the words "Jain sequences" and "Moore-Read", and revised as (line #103):
"We demonstrate that the electric displacement field D can drive transitions between **odd-denominator** and **even-denominator states** by inducing Landau level crossings."

(4) In the main text, we added one sentence in line #236:
"We emphasize that these identifications are based on transport signatures; direct probes of the underlying quasiparticles are left for future work."

(5) In Fig. 3g, the caption has been revised to (line #518):
"Identification of the **predicted** Moore-Read type for all observed half-filled states based on the calculations (see SI), …"

These changes ensure that our discussion of possible non-Abelian order is presented as a theoretically motivated interpretation rather than a firm experimental claim.

2. It will be extremely important and useful if authors can provide a diagram of LL crossings as a function of D, for ZLL of this system. It might be important to know which N=1 orbital (bernal bilayer or ABC trilayer part) is responsible for even-denominator states.

Reply: We thank the referee for this very helpful suggestion. We fully agree that visualizing the Landau level (LL) evolution as a function of displacement field D is essential for clarifying the origin of the even-denominator fractional states. Following this advice, we now provide a more explicit LL phase diagram that tracks the LL character as a function of D and filling factors.

Specifically, we preformed single-particle LL calculations (presented in the Supplementary Information, Fig. S1&2) and extracted, for each LL, the average weight on the Bernal (AB) layer and rhombohedral (ABC) layer, as well as the valley index. The resulting schematic diagram is shown in Fig. R1 of this Reply:

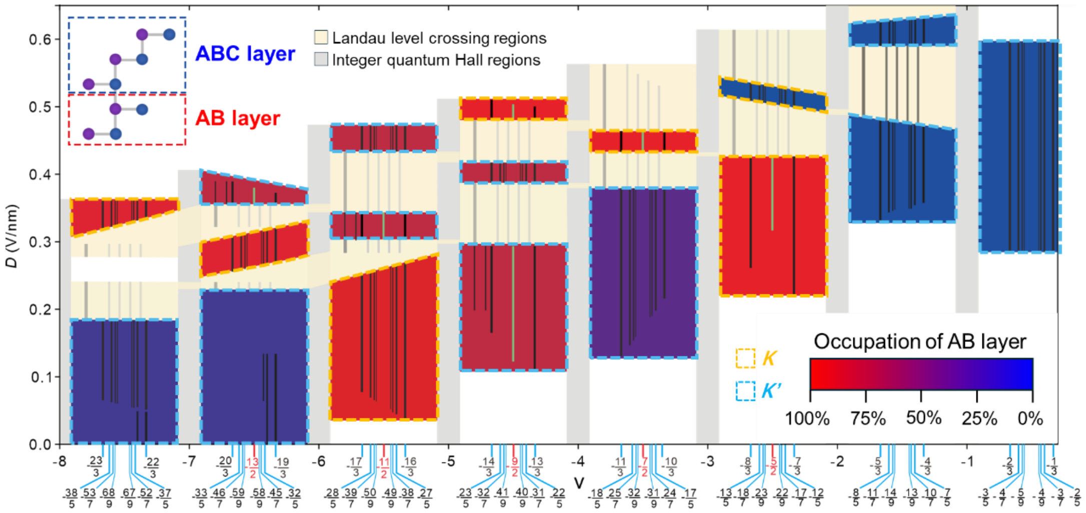

Fig. R1. Schematic phase diagram labeling the ground-state order for the same filling fraction range as shown in Fig.1 d of the main text. Regions enclosed by light-blue and dark-yellow dashed lines correspond to broken-symmetry states with valley index *K* and *K'*, respectively. The color filling encodes the layer occupation of the LLs: red denotes AB character, blue donates ABC character, and intermediate colors indicate mixed AB/ABC contributions. Even-denominator regions around filling factor -9/2 (higher *D*), -7/2, -5/2 exhibit ≈93.8% occupied with AB layer, and those around -13/2, -11/2, -9/2 (lower *D*) exhibit ≈75.1% AB-layer occupation.

This analysis reveals that the even-denominator states are predominantly contributed by the AB-layer-derived orbitals, with an average AB occupation exceeding 75% across all observed even-denominator FQH states. Moreover, we find that the two families of even-denominator states originate from different valleys: the −9/2, −7/2, and −5/2 states arise from the *K* valley, whereas the −13/2, −11/2, and −9/2 states are from the *K'* valley. This valley distinction naturally explains the different appearance and relative robustness of the two cascades of even-denominator FQH states in the experimental phase diagram.

In the revised main text, we have used this Fig. R1 to replace the original bottom panel of Fig. 1b.

3. For the ground states to be non-Abelian, the wavefunctions should be single component in both spin and valley quantum numbers. Have authors performed in-plane magnetic field and D-dependent measurements on the observed even-denominator states?

Reply: We thank the referee for raising this important point. We agree that non-Abelian

even-denominator states are expected to be single component, i.e., fully spin and valley polarized.

In principle, one diagnostic of full spin polarization is the response of the energy gap to an in-plane magnetic field. If the ground state is fully spin polarized, varying in-plane B should have little influence on the gap. Unfortunately, our current experimental setup only supports out-of-plane magnetic field application and does not include an in-situ sample rotator, so we are presently unable to perform systematic in-plane B field measurements. Considering our experimental setup is a top-loading wet dilution refrigerator with 18 Tesla superconductor magnet, sample space is quite limited, so it is challenging to add a rotator to the probe in this system. So we are unable to do in-plane B field measurement during preparation of this Reply.

We do, however, explore the dependence of the half-filled states on the D field, which simultaneously tunes the orbital character, valley polarization, layer polarization, and effective LL mixing in this multiband system. From our measured R_{xx}-n-D map, the observed even-denominator FQH states appear under finite displacement field, and they are strengthened by the increased D at certain ranges, e.g., -5/2 state shown in Fig. R2a and b. This may suggest the layer polarization nature of this FQH state. But it should be mentioned that D has complexed influence to orbital, valley, layer and LL mixing in this ABCBC graphene, so we cannot clarify further about the ground state.

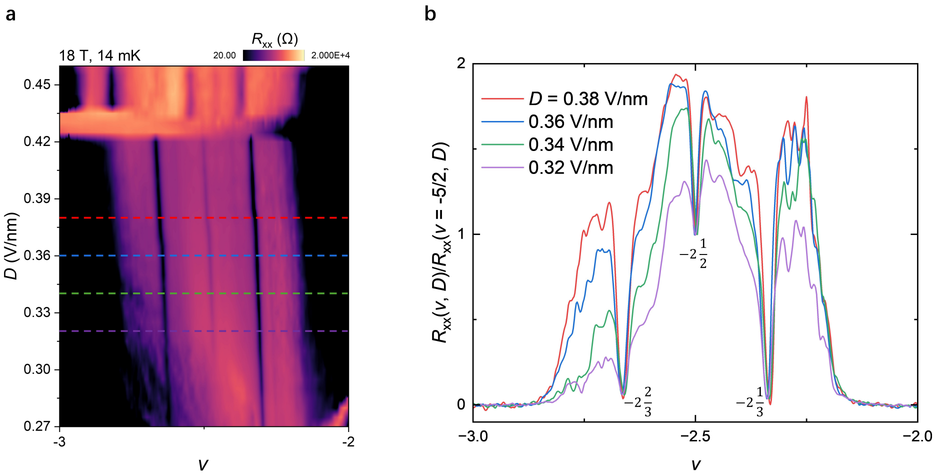

Fig. R2. The influence of D on the stability of even-denominator FQH. a, R_{xx} as a function of filling factor ν and displacement field D at T = 14 mK and B = 18 T. **b**, corresponding linecuts taken from the colored dashed lines in **a**, the resistance is normalized as $R_{xx}(\nu, D)/R_{xx}(\nu = -5/2, D)$. It is obvious that the dips at −2(1/3,1/2,2/3) are more pronounced with increased D.

The complexed role of D has actually been discussed even for a simpler system, e.g., bilayer graphene with a single band. The D dependence gap of even-denominator FQH in bilayer graphene shows not well understood behavior (ref: Li, J. I. A. et al., Science 358, 648–652 (2017)): the gap decreases with increasing D, but the system is presumed to be fully layer-

polarized since the observed the gap to persist to large displacement fields. In our case of ABCBC pentalayer, *K* and *K'* is not directly coupled with top and bottom layer, which can be checked from Fig. 2d in Supplementary Information. Thus, the increased *D* may not favor layer nor valley polarization.

We fully agree that a direct probe of spin polarization via in-plane field would be highly valuable, and we plan to implement such measurements in future work once our cryogenic setup is upgraded to include a sample rotator.

4. It seems the data in Fig. 1d is patched. Was it taken during different cooldowns?

Reply: We thank the referee for the careful inspection of Fig. 1d. For technical reasons, we took the data at different *n*-*D* windows and assembled them together as Fig. 1d. Importantly, all datasets used to construct Fig. 1d were acquired during the same cooldown, under identical experimental conditions. The apparent patch boundaries reflect only the stitching of measurement windows and do not correspond to any physical discontinuity in the device behavior.

5. In Fig. 1d, are there additional crossings at ν=−13/2 (in pink shaded area) around D≈0.37 V/nm?

Reply: We thank the referee for this insightful question. To address it, we examined the region around ν =−13/2 in a zoomed-in R_{xx}–*n*–*D* color plot in Fig. R3. We can find that a sudden increase in resistance appears above the white dashed line. This feature could be consistent with an additional LL crossing; however, the data quality in this region is noticeably degraded, likely due to increased contact resistance and local disorder at large *D*. Because the nature of this feature cannot be reliably determined from the present dataset, we chose not to include it in the schematic phase diagram of Fig. 1d. The revised schematic therefore shows only those crossings and phases that are clearly resolved.

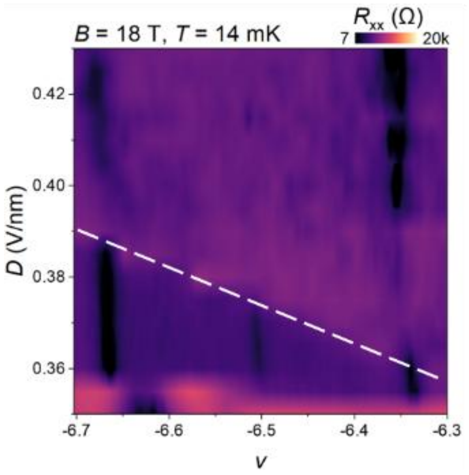

Fig. R3. Zoomed-in R_{xx}-ν-D color plot around ν = -13/2.

6. What do the grey shaded areas in Fig. 1d represent at high D between $-1<\nu<0$ and $-2<\nu<-1$?

Reply: We thank the referee for the careful reading of Fig. 1d. The grey-shaded regions indicate areas where the longitudinal resistance R_{xx} exceeds 20 kΩ (the upper limit of the color scale), corresponding to high-resistance or insulating regimes.

7. Did authors measure between fillings -8 to -10, as the ZLL spans fillings from -10 to +10? Have authors measured on electron-side? It would be interesting to see if the LL crossings are symmetric across charge neutrality.

Reply: We thank the referee for this constructive question. In the device, reliable measurements could be obtained up to filling factors of approximately $\nu = -8$ on the hole side; beyond this range, gate leakage and reduced carrier mobility under high displacement field limited access to well-developed quantum Hall features higher fillings.

We also preformed measurements on electron side. However, despite using the silicon back gate to reduce contact resistance and suppress p-n junctions, the signal quality remained limited: many regions are highly resistive and fractional states are generally unresolved. Only a few odd-denominator FQH states can be distinguished, as shown in Fig R4. Because the data on the electron side are sparse and do not reveal additional even-denominator states or qualitatively new features, we didn't include them in the main text.

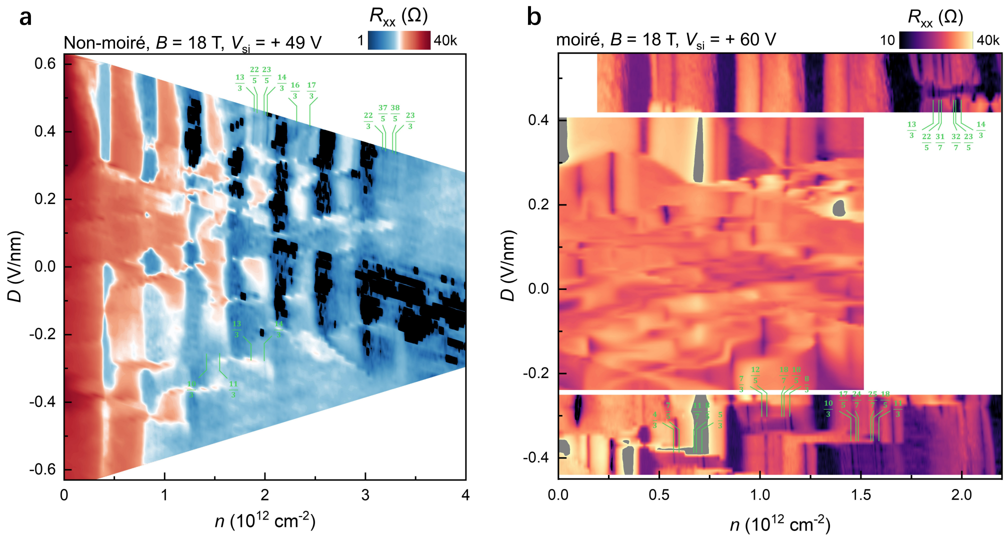

Fig. R4. Transport on electron side. a and **b**, R_{xx}-n-D color plot at B = 18 T, T = 14 mk for the non-moiré and moiré devices, respectively. Silicon back gates were biased at +49 V (non-moiré) and +60 V (moiré) to reduce the large contact resistance arising from p–n

junctions. The signal quality on the electron side is limited, and fractional quantum Hall states are generally unresolved in most regions for both devices. Only several odd-denominator FQH states are discernible, highlighted by green lines.

Regarding the symmetry of LL crossings across charge neutrality, the LL spectrum in ABCBC pentalayer is intrinsically electron-hole asymmetric and further modified by broken inversion symmetry. The electron – hole asymmetry already exists in single-particle band structures at zero magnetic field (Fig. R5a-d). This can be understand that the ABCBC graphene has non-centrosymmetric lattice and should show different behavior at $\pm|\Delta|$ (the calculation in Fig. R5c-d also shows the asymmetry). In the magnetic field, the asymmetry of the LL crossings is clearly observed in our transport data shown in Fig. R5e (B = 6 T), where LL crossings at electron and hole sides, marked as red and blue lines, respectively, are obviously different.

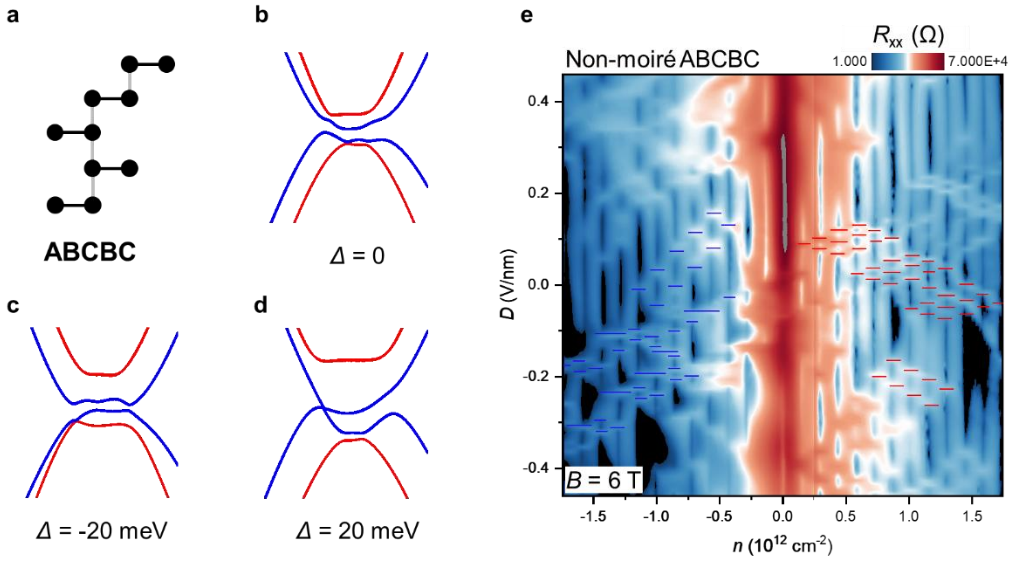

Fig. R5. Non-centrosymmetry of ABCBC. a, Lattice structure of ABCBC, which shows no mirror nor inversion symmetry, it has non-centrosymmetry. **b~d**, Band structure of ABCBC with interlayer potential difference Δ = 0, -20 meV, 20 meV, respectively. **e**, R_{xx}-n-D color plot when B = 6 T for non-moiré device. Some of the LL crossing regions are indicated with red and blue lines.

8. What do the grey lines in the schematic of Fig. 1d represent? They are not mentioned in the figure caption.

Reply: We thank the referee for this careful observation. The grey lines in Fig. 1d denote quantum Hall states that originate from the moiré superlattice, i.e., Hofstadter butterfly states. The same states are more clearly visible as green lines in the Landau fan diagram of Extended Data Fig. 4d (also reproduced in Fig. R6), where their Chern numbers are extracted from the Streda formula and labeled.

To avoid confusion, we have updated the Fig.1 caption to explicitly state:

"Grey lines in the schematic denote moiré-induced integer quantum Hall states (Hofstadter minibands), corresponding to the green lines labeled with Chern numbers in Extended Data Fig. 4d."

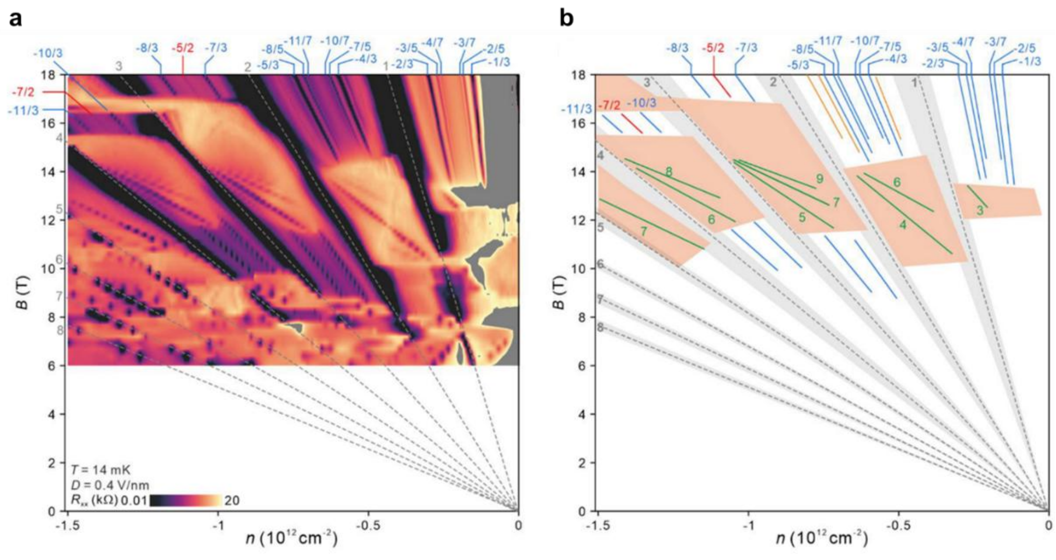

Fig. R6. Landau level fan diagram. a, R_{xx} - n - B color plot of ABCBC at D = 0.4 V/nm when T = 14 mK. **b**, Corresponding phase diagram concluded from **a**, the integer, even-denominator fractional and moiré-related quantum Hall states are indicated by grey, red and green lines. The odd-denominator fractional quantum Hall states with two and four flux quanta are indicated by blue and orange lines, respectively.

9. In Fig.3, except panel d, the Rxy plateau value for nu= - i - 1/3 (i being an integer) fillings is much lower than the expected Rxy, however Rxx is fully developed. Can authors comment on this?

Reply: We thank the referee for this insightful question. We agree that in Fig. 3 the Hall plateau values at ν = - i - 1/3 appear lower than the ideal quantized values, despite well-developed minima in R_{xx}. We attribute this to two experimental factors:

(1) Device nonuniformity. As illustrated in Fig. R7a, longitudinal voltages measured between different probe pairs (V_{xx}-1 and V_{xx}-2) show noticeably different quality (Fig. R7b, c), suggesting that different segments of the Hall bar host slightly different electronic phases. For example, the region between probes #1 and #3 may already be in the ν = -7/3 state, while part of the region between probes #3 and #4 remains in the ν = -2 state. The measured Hall voltage between probes #3 and #4 thus reflects a mixture of ν = -7/3 and ν = -2, leading to an apparent reduction of σ_{xy}.

The nonuniformity of our device may also induce backscattering of edge states. Ideally, edge channels are dissipationless only when the bulk is uniform and fully incompressible. However, due to the finite quality of our device, residual conducting domains can appear, leading to backscattering and further suppression of σ_{xy}.

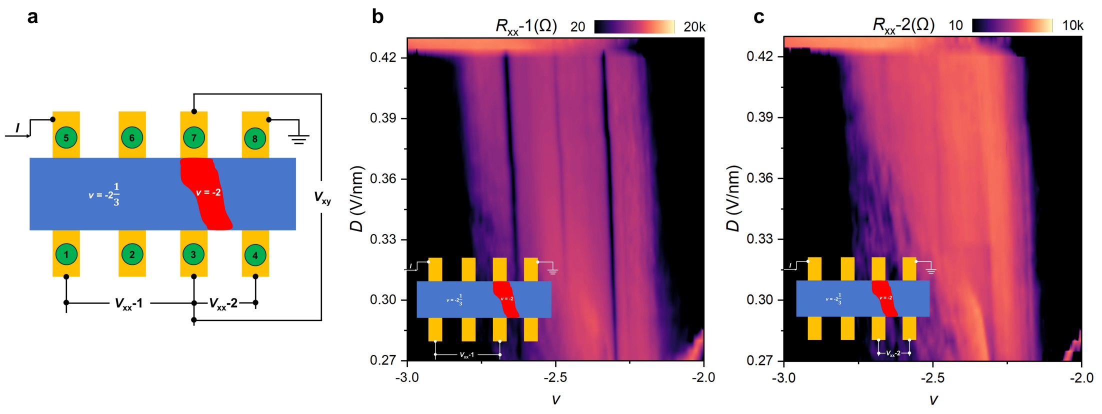

Fig. R7. Non-uniformity of ABCBC moiré device. **a**, A schematic of device geometry and measurement setup. **b-c**, Comparison of data quality between different measurement probes.

(2) Residual longitudinal admixture. Although our device is patterned into a Hall-bar geometry, slight misalignment between voltage probes can mix a small the longitudinal component into the transverse signal. To mitigate this, we antisymmetrized σ_{xy} with respect to $B = \pm$ 18 T (original data at $\pm$ 18 T are shown in Fig. 3 of the main text and Extended Data Fig. 6), as shown in Fig. R8a–f. After antisymmetrization, the σ_{xy} values for ν = -i-1/3 fillings become much closer to the expected quantized values.

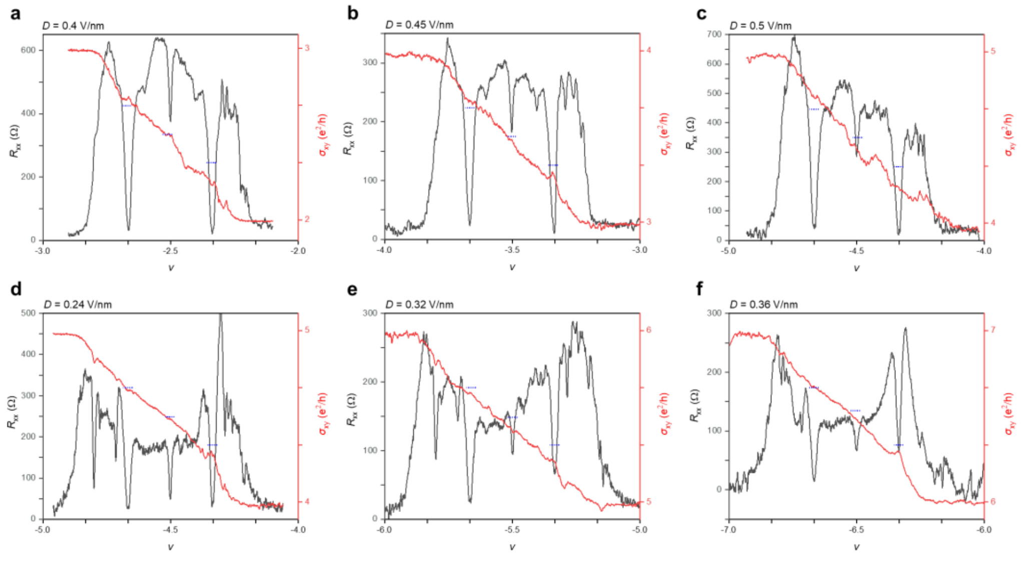

Fig. R8. Antisymmetrized σ_{xy} at $|B|$ = 18 T for different FQH states.

10. Do authors understand why Jain states of four-flux attached composite fermions appears along with the appearance of -9/2,-11/2, and -13/2 (pink shaded areas in schematic of Fig.1d)

while they are absent with -5/2 and -7/2 (purple shaded areas)? Is it related to the LL orbital index?

Reply: We thank the referee for this insightful question. The referee is right that the appearance of the CF_4 states at filling factors −9/2, −11/2, and −13/2, and their absence at −5/2 and −7/2, is closely related to the LL orbital character. This correlation is directly manifested in the pseudopotential coefficients of the corresponding LLs, as illustrated below.

Compared with the non-relativistic LLs in GaAs, the pseudopotentials in 5-layer graphene take into account the hybridization of Landau level orbitals and can be expressed as

$$V_m = \sum_{i_1=1}^{10}\sum_{i_2=1}^{10}\sum_{j_1,j_2,j_3,j_4} \alpha^*_{i_1,j_1}\alpha_{i_1,j_3}\alpha^*_{i_2,j_2}\alpha_{i_2,j_4}\delta_{m'+j_1+j_2,m+j_3+j_4}$$

$$\cdot\int \frac{d^2q}{(2\pi)^2} V(\vec{q})e^{-q^2}F_{j_1,j_3}(\vec{q})F_{j_2,j_4}(-\vec{q})F_{m,m'}(\sqrt{2}\vec{q}).$$

Specifically, for CF_2 states such as $\nu = 1/3$, the relevant zero-energy state is primarily determined by the short-range pseudopotential V_1, whereas for CF_4 states such as $\nu = 1/5$, the corresponding zero-energy condition involves V_1 and V_3 .Therefore, CF_2 states are stabilized when V_1 is relatively strong, while CF_4 states require a more significant contribution from V_3.

By comparing the pseudopotentials of the two LL orbitals involved (as shown in Fig. R9), we find that the LL associated with larger absolute filling factors (labeled band ② in Fig. S2(c)) exhibits a smaller V_1 but a larger V_3 over a certain range, which naturally favors the emergence of CF_4. Meanwhile, strong LLM breaks particle–hole symmetry, leading to the appearance of CF_4 states only on one side of the filling sequence. Furthermore, strong LL mixing can drive the formation of Wigner crystal–like phases, which may suppress or destabilize the CF_4 states (C. Wang et al., Phys. Rev. B 111, 085429 (2025)).

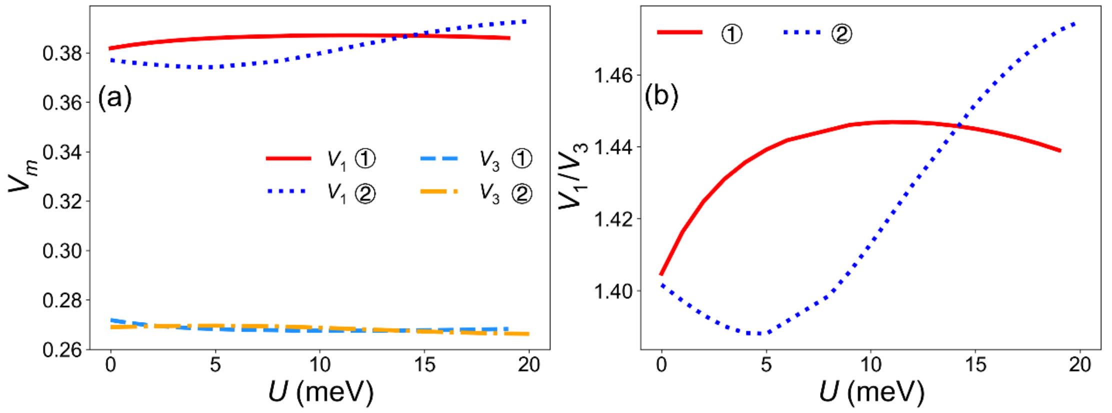

Fig. R9. Pseudopotentials calculation. a, Pseudopotentials of the two LL orbitals (labels ① and ② adopted from Fig. S2(c)). **b,** Ratio of V_1/V_3 for the two LL orbitals.

In the revised version, we have added corresponding discussions in the Supplementary Information to explain why the CF_4 states appear only at −9/2, −11/2, and −13/2.

The added discussions start from Supplementary line #195 to Supplementary line #206: “Another intriguing feature is that the fractional states −5/7, −9/5, −9/7, −12/7, −14/11, and −19/11 appear alongside the −9/2, −11/2, and −13/2 sequence, and only on one side of the sequence, while they are absent in the −5/2, −7/2 and -9/2 sequence. This behavior is closely related to the LL orbital character, which is directly reflected in the pseudopotential structure of the corresponding LLs. As shown in Fig. S16, by comparing the pseudopotentials of the two LL orbitals involved, we find that the LL associated with larger absolute filling factors (labeled band ② in Fig. S2(c)) exhibits a smaller V_1 but a larger V_3 over a certain range. This naturally favors the emergence of the 1/5 state and its corresponding hierarchy, because 1/5 state, compared to 1/3 state, is more sensitive to stronger V_3. Meanwhile, strong LL mixing breaks particle–hole symmetry, leading to the appearance of these states only on one side of the filling sequence. Furthermore, strong LL mixing can drive the formation of Wigner crystal–like phases, which may suppress or destabilize these states [14].”

Fig. R9 has been added as Fig. S16 in Supplementary line #298.

11. In the sample without moiré, Extended data Fig.5, the even-denominator states are barely visible. Is this due to poor bulk quality of the sample or moiré pattern has a role in stabilizing the even-denominator states in the sample with moiré? Authors should investigate it more by measuring more samples without moiré.

Reply: We thank the referee for raising this important point. As noted in the manuscript, we have characterized a second ABCBC-5LG device that does not show a detectable moiré superlattice (Extended Data Fig. 5). In that device the overall phase diagram is qualitatively consistent with the moiré device, but the even-denominator features are much weaker and, in many ranges of D and ν, not resolved. We attribute this difference to a combination of sample-to-sample variability and the subtle role played by the weak moiré potential in our device:

(1) Sample quality and disorder. Small differences in carrier mobility and disorder between devices strongly affect the visibility of fragile correlated states. The non-moiré device exhibits smaller mobility (~100,000cm^{-2}/V.s) than the moiré one (~160,000cm^{-2}/V.s), this difference can also be reflected in Landau fan diagram in Fig. R10a&b, in which, the quantum Hall states indicated by orange dashed lines develop from 0.75 T for non-moiré device but 0.14 T for moiré device, indicating much higher mobility for the moiré device. And the intrinsic doping for non-moiré device is 7.2×10^{10} cm^{-2} but for the moiré one it is 8×10^{9} cm^{-2}, this can be seen from the charge neutral points (CNP) indicated by green arrows in Fig. R10a&b. The disorder of each device can be captured from the Hall measurement shown in Fig. R10c&d, where the disordered regime occupies density range of $n \approx \pm 8.2 \times 10^{10}$ cm^{-2} and $n \approx \pm 4 \times 10^{10}$ cm^{-2} for non-moiré and moire device, respectively. Overall, the sample quality for moiré device is better than non-moiré device, so the fragile even-denominator FQH should be more visible in high-quality device.

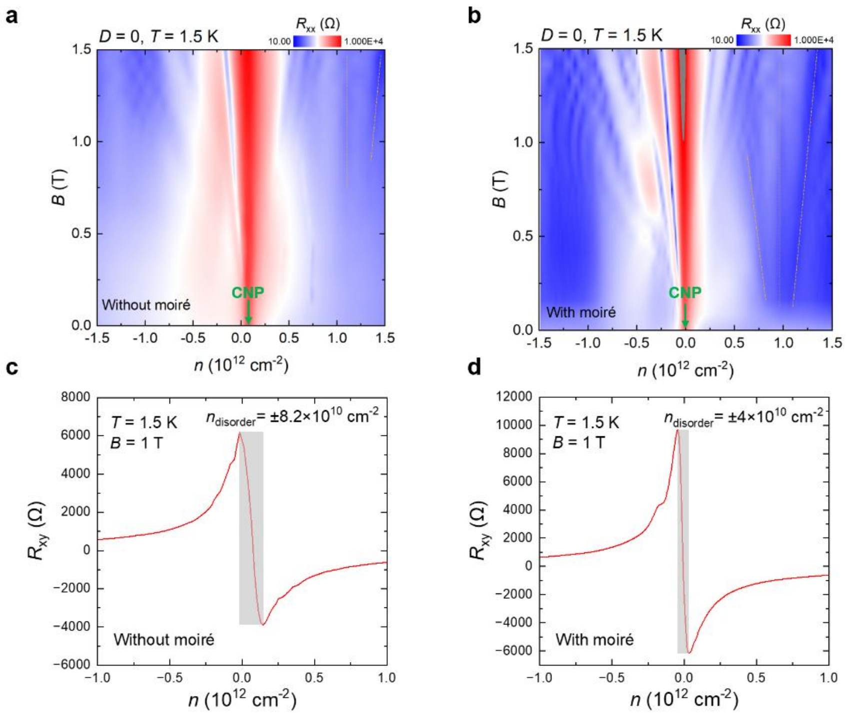

Fig. R10. Comparison of sample quality and disorder between two devices. a and **b**, Landau fan diagram for non-moiré and moiré device at **D** = 0. **c** and **d**, Hall resistance for non- moiré and moiré device measured at B = 1 T.

(2) Consistency between two devices. Although the non-moiré device exhibits relatively lower sample quality, its quantum Hall phase diagram is consistent with that of the moiré device. We compared the R_{xx}–n–D color plots and extracted the corresponding FQH phase diagrams for both devices (Fig. R11a&b). In the non-moiré sample, only the ν = −5/2 and -11/2 even-denominator FQH states are resolved within D = 0.15 – 0.38 V/nm and 0.33 – 0.37 V/nm, which is consistent with the moiré device where ν = −5/2 occurs in D = 0.3 – 0.4 V/nm, ν = -11/2 in D = 0.31 – 0.36 V/nm. The small difference in range likely arises from contact resistance or local disorder, which obscure certain regions of the map. Other even-denominator states (−7/2, −9/2, −13/2) are not directly resolved in the non-moiré device; however, this absence does not necessarily imply that such states are missing.

First, the reduced sample quality likely masks these fragile states. Second, indirect signatures suggest their presence: whenever an even-denominator state emerges at N+1/2, the adjacent odd-denominator FQH states following the Jain sequence (e.g., N + (2/5, 3/7, 4/9, 6/11, 5/9, 4/7, 3/5)) become strongly suppressed or vanish. This trend is evident in Fig. 2 a, b and Fig. 3a–f of the main text, where odd-denominator states N + (2/5, 3/7, 4/9, 6/11, 5/9, 4/7, 3/5) are pronounced in the absence of an even-denominator state at N+1/2, but are much weaker when an even-denominator state appears. Similar competition between even- and odd-denominator states has been reported in other systems (Phys. Rev. Lett. 59, 1776 (1987); Nature 549, 360–364 (2017); Nat. Nanotechnol. 15, 569–573 (2020); Nat. Commun. 15, 6236 (2024)).

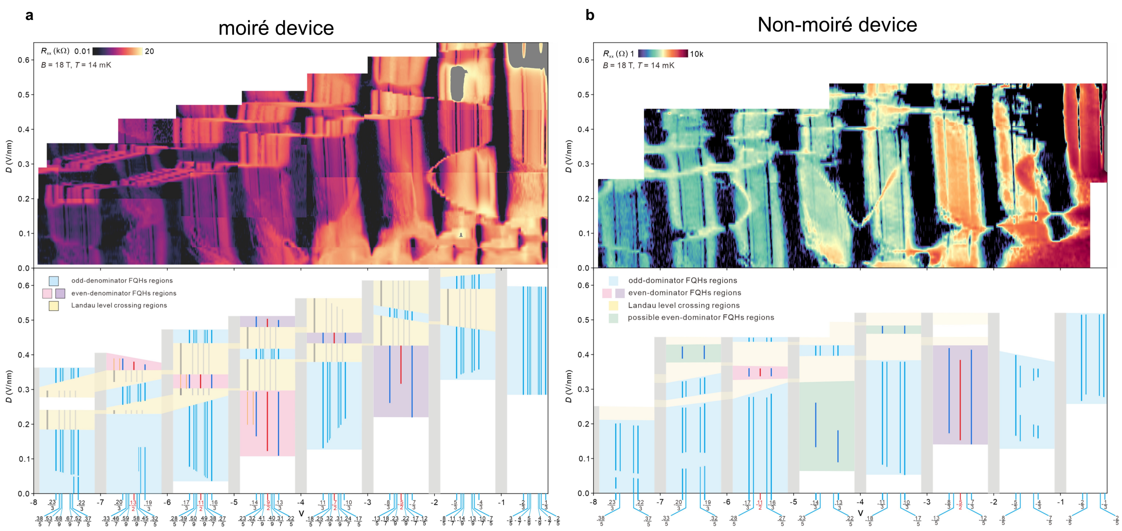

Fig. R11. Comparison of phase diagrams between two devices. a and **b**, R_{xx} as a function of filling factor ν and displacement field D at T = 14 mK and B = 18 T for moiré and non-moiré devices, respectively. Bottom panel: sketch map of each top panel.

In bottom panel of Fig. R11b, regions where N + (1/3, 2/5, 3/5, 2/3) states are well resolved are filled with light blue, while those showing only N + (1/3, 2/3) are filled with light green. The latter regions likely harbor even-denominator FQH states that are obscured due to the limited quality of the non-moiré sample. The overlap between these possible regions in non- moiré device and the even-denominator regions in the moiré device further supports their intrinsic origin.

(3) Moiré as a secondary stabilizing factor. In our device, the moiré potential is relatively weak (period ≈ 12.8 nm), and Hofstadter features are prominent only near LL crossings. We therefore do not regard the moiré as essential for the existence of even-denominator states. Rather, it can locally modify the density of states and effective band dispersion near crossings, sometimes enhancing the visibility of fragile gaps. The fact that the non-moiré device reproduces the same overall FQH hierarchy (albeit with weaker half-filling signatures) supports the conclusion that the cascade of even-denominator states is intrinsic to the ABCBC band structure.

(4) On additional non-moiré samples. We fully agree that measurements on more non-moiré devices would be valuable. However, fabricating high-quality ABCBC-5LG heterostructures is extremely challenging, with typical yields below 10%. Given that we already have one moiré and one non-moiré device showing consistent phase diagrams, we believe the essential physics is robustly captured by the current dataset.

To better compare the similarity between moiré and non-moiré device, we replace the original Extended data Fig. 5 by Fig. R11.

12. In line 170, there seems to be a typo: the word “red” should be replaced with “pink”.

Reply: We thank the referee for catching this typo. The color label has been corrected from “red” to “pink” in the revised manuscript (line #175).

13. In line 164, there seems to be typo: the word “-2/9” should be replaced by “-9/2”.

Reply: We thank the referee for pointing out this typo. The filling factor has been corrected from “−2/9” to “−9/2” in the revised manuscript (line #169).

Reviewer #2 (Remarks to the Author):

The paper deals with a multilayer graphene stack that exhibits some FQHE features, which have been observed experimentally. This work is a sequel to a previous paper by this group of authors, already published in Nature Communications (Nat. Commun. 15, 6236 (2024) [24]) focused on similar observations in a 3-layer graphene stack (now extended to a 5-layer stack). Both papers are similar, and the new one repeats the same experimental procedures applied earlier to multilayer graphene heterostructures.

Reply: We thank the reviewer for this comment. We would like to clarify that our group is independent from the authors of the previous work published in Nature Communications (Nat. Commun. 15, 6236 (2024)). Although both studies explore fractional quantum Hall features in multilayer graphene, the two works are entirely independent, involving different samples.

Scientifically, the systems investigated are fundamentally distinct. The earlier work focused on ABA-stacked trilayer graphene, whereas our study examines ABCBC-stacked five-layer graphene, whose band structure, Landau-level hybridization, and FQH state hierarchy are qualitatively different. In particular, the number and sequence of even-denominator states observed here differ from those reported previously. Furthermore, the five-layer configuration studied here has not been explored in prior transport experiments, making this work an independent and original contribution to the understanding of correlated fractional states in multilayer graphene.

Experimentally, both papers are of a very high quality, and the obtained data are presented with sufficient care. However, the theoretical (explanatory) part of both papers is confusing -- in the current paper, to the same extent as in the previous publication by the same group [24]. These authors ignore that the "single quasiparticle CF approach" to FQHE in multilayer Hall systems does not work, as has already been clarified in the literature, especially in detail for bilayer graphene and double-layer GaAs heterostructures. Therefore, in multilayer Hall systems, the CF model cannot be applied to explain the physics behind the odd FQHE features observed in 3- and 5-layered Hall systems.

Reply: We thank the reviewer for the positive assessment of the experimental quality and for raising this important point concerning the applicability of the CF theory to multilayer Hall systems. We fully agree that, in general, in multicomponent quantum Hall systems, the single-component CF picture is not directly applicable.

However, under the very high magnetic field used in our experiment (18 T), all IQH fillings are observed, implying a complete lifting of spin and valley degeneracies in the Landau levels. This implies that, for the even-denominator states which constitute the main focus of our study, the system is effectively single-component.

In this regime, the fractional states corresponding to CF2 exhibit linear gap scaling in quantitative agreement with the CF theory. For the fractional states that may correspond to CF4, we do not yet have sufficient experimental evidence to confirm that a single-component CF interpretation remains fully valid—nor is this the main focus of the present manuscript. Accordingly, in the revised manuscript, we have removed the discussion of CF_4 to avoid overreliance on the standard single-component CF model when describing the odd-denominator states.

Original sentence in line #128: “They are flanked by nearby odd-denominator states (blue and orange lines) belonging to Jain sequences with two or four flux quanta (CF2 and CF4).”
Revised sentence in line #131: “They are flanked by nearby odd-denominator states (black lines) that organize into Jain sequences with two flux quanta (CF2), together with further fractional features at nearby fillings that go beyond the simple CF2 hierarchy.”

Original sentence in line #146: “We also observe several states consistent with CF4 (four-flux) at higher fillings, including -5/7, -9/5, -9/7, -12/7, -14/11, and -19/11 (yellow region, following $\nu = p/(4p \pm 1)$), though these are weaker.”
Revised sentence in line #152: “We also observe several states at higher fillings, including -5/7, -9/5, -9/7, -12/7, -14/11, and -19/11 (yellow region), though these are weaker.”

Original sentence in line #444: “The Jain sequence of two-flux CFs (CF2), is evident in the blue region, while several four-flux CFs (CF4) states are also observed in the orange shading region.”
Revised sentence in line #501: “The Jain sequence of two-flux CFs (CF2), is evident in the blue region, while several states (possible four-flux CFs) lying beyond the primary CF2 hierarchy are also observed in the orange shading region.”

Original sentence in line #457: “Phenomenologically, a hallmark of the second group is the appearance of several CF_4 states (orange shading) accompanying each half-filled state on the left, in contrast to the first group where such states are absent.”
Revised sentence in line #515: “Phenomenologically, a hallmark of the second group is the appearance of several states beyond the CF2 sequence (orange shading) accompanying each half-filled state on the left, in contrast to the first group where such states are absent.”

Original sentence in line #519: “…including the accompanied CF4 state in the second (pink shading) group.”
Revised sentence in line #580: “including the accompanying states beyond the CF_2 hierarchy in the second (pink shading) group.”

The CF theory is an auxiliary quasiparticle-type model for the topological effect of multiloop cyclotron braid orbits responsible for FQHE. The CF model only partially aligns with the deeper, current understanding of FQHE -- CF model works exclusively in the lowest LL of GaAs and monolayer graphene (except for the so-called enigmatic states and even-denominator

fractions) and fails completely in higher LLs and in multilayer Hall systems. The latter is the case of the experimental study presented in this submission.

This limitation of the CF model usage is completely ignored in the submission. Its authors suggest some artificial fluxes added to electrons, mixing them with “chiral gravitons”, “pairing of CFs”, “quasiparticle-type phase transitions” and other rather irrelevant concepts, in an attempt to find an interpretation (which is, however, impossible within the CF framework).

Reply: We thank the reviewer’s comment. We fully agree that the single-component CF picture is not appropriate for describing multicomponent FQH states. As we mentioned before, the system studied here is effectively single-component. In ABCBC-stacked 5-layer graphene, the Landau levels corresponding to filling factors between −10 and 10 (which is the range explored in our study) are generally referred to as the zeroth LL, rather than higher LLs; their single-particle wavefunctions are complicated that consist of many non-relativistic Landau orbitals.

We also would like to emphasize that our key numerical findings—such as the sixfold ground-state degeneracy on the torus and the chiral graviton spectral function—are obtained independently of any CF-based assumptions. These results provide direct numerical and topological evidence for a non-Abelian Pfaffian–type state.

Regarding the even-denominator fractions, we agree with the reviewer that the conventional CF model cannot capture these states. Indeed, as established in the literature for GaAs and graphene systems (A. Sharma et al., Phys. Rev. B 109, 035306 (2024); C. Wang et al., Proc. Natl. Acad. Sci. U.S.A. 120 (52)(2023); A. A. Zibrov et al., Nature 549, 360–364 (2017); J. I. A. Li et al., Nature Physics 15, 898–903 (2019)), some of these even-denominator FQH states may be understood as paired CF states. Our understanding is that these studies represent the consensus of the physics community, with which we tend to agree. Our experimental results do not provide any evidence contradicting this theoretical framework. Of course, we acknowledge that several aspects of these states remain open and deserve further investigation in future studies.

The interesting observation of the lifting of some FQHE features by the application of a vertical electric field (referred to as "tunability") is especially falsely interpreted in terms of “symmetry breaking” or crossing single-particle bands. This is a misinterpretation, likely partly induced by the experimental paper by Maher et al. [21], which was also originally incorrectly interpreted. In fact, the lifting (tuning) of the FQHE hierarchy by the application of vertical voltage can be understood via a simple topological effect: the blockade of interlayer hopping of carriers against the electrical field. This prevents the division of the loops of multiloop cyclotron braid orbits among the layers (thus reducing the hierarchy of a multilayer system to that of a monolayer system), as detailed in the quantum topological approach -- cf. doi:10.1038/s41598-017-09166-5, Supplementary Information. Note that the reasons for the even denominator filling fractions in the FQHE hierarchy are at least threefold: the pairing of

carriers (mentioned in the submission), specific topological commensurability invariants even in the LLL (e.g., doi.org/10.1088/1361-648X/ad7b92), and the division of loops of a multiloop braid orbit among layers in multilayer Hall systems -- all three of which can compete energetically.

Reply: We thank the reviewer for this thoughtful comment and for sharing their interpretation based on the topological (braid group) perspective.
We acknowledge that our original wording—originally in line #36-39: "These even-denominator states … exhibit unprecedented displacement-field tunability driven by LL crossings in the hybridized multiband structure" — may lead to confusion. Our intention was not to imply a simple single-particle band-crossing mechanism. Rather, this statement was meant as a phenomenological description of the experimental tunability that originates from the hybridization between Landau levels of the ABC-trilayer and AB-bilayer graphene subbands. From the interaction perspective, this hybridization effectively modifies the intra-ZLL pseudopotentials, thereby influencing the relative stability of the even-denominator fractional states. In this sense, the "LL crossings" reflect the experimentally observed evolution of hybridized Landau level manifolds under displacement field, rather than literal single-particle LL crossings.

From the interaction perspective, this hybridization effectively modifies the intra-ZLL pseudopotentials, thereby influencing the relative stability of the even-denominator fractional states. Therefore, the statement does not refer to crossings of single-particle bands.

In the revised manuscript, we have rephrased the sentence to avoid this possible misunderstanding:

Starting from line #38: "… exhibit displacement-field tunability associated with the hybridized multiband structure in the ZLL."

As mentioned earlier, our numerical calculations—performed independently of the composite fermion picture—indicate that the observed even-denominator states correspond to a non-Abelian Moore–Read–type state, which falls into the first category mentioned by the reviewer (pairing of carriers), rather than the multicomponent scenario (the third category). Of course, we cannot completely rule out that experimentally some half-filled states may correspond to a Halperin (331)–type state, which will be an interesting direction for future investigation. The second category mentioned by the reviewer, namely the quarter-filled even-denominator states related to specific topological commensurability invariants, is beyond the scope of the present study.

Therefore, the theoretical side of the submission must be revised because, in its current form, the paper is clearly incorrect and cannot be published. The same concern applies to the introductory and concluding paragraphs, in which similarly confusing interpretations are repeated. On the formal side of the paper, please clarify what is meant by the equal

contribution of the three first authors with respect to the other authors (who are not marked).

Reply: We thank the reviewer for the careful evaluation of our work and for providing an alternative theoretical perspective on the FQHE. While we appreciate this viewpoint, our experimental findings are not in contradiction with the existing theoretical understanding. In the revised manuscript, we have corrected and clarified several statements that could lead to misunderstanding.

We respectfully disagree with the reviewer's assessment that our theoretical interpretation is "incorrect." First, the fractional states corresponding to CF_2 exhibit linear energy-gap scaling that is in quantitative agreement with the composite fermion theory. Second, our main numerical analysis of the even-denominator states is entirely independent of any CF theory. Third, the description of even-denominator states as paired CF states—supported by extensive prior studies in both GaAs and graphene systems—represents the prevailing consensus within the physics community, and our experimental findings do not contradict this understanding.

Regarding the authorship statement, the notation "equal contribution" refers to the three first authors who mainly contributed to the experimental and theoretical parts, as specified in the updated author contribution section of the revised manuscript.

We have added **Author contributions** from line #471:
"G.C. supervised the project. Y.S. and K.L. fabricated the devices and performed the transport measurements with the assistance of S.L., Z.G., J.G. B.T., G.L., L.L., Y.Z and Z.S. C.J and Z.H. did all the theoretical calculations in the supplementary with the assistance of D.Y. M.T., N.W., K.W. and T.T. grew the hBN single crystals. Y.S., K.L., C.J, Z.H. and G.C. analysed the data. Y.S., K.L., C.J, Z.H. and G.C. wrote the paper with input from all authors."

Reviewer #3 (Remarks to the Authors):

The authors report on the fractional quantum Hall phase diagram of pentalayer graphene. Pentalayer graphene has attracted significant interest recently, following the observation of fractional quantum anomalous Hall states and various superconducting states. The authors carefully isolated mixed-stack pentalayer graphene and provide the first experimental map of its quantum Hall phase diagram through conductance measurements.

This phase diagram is particularly interesting due to the appearance of numerous even-denominator fractional quantum Hall states, which are thought to host non-Abelian excitations useful for decoherence-free quantum computing. Their findings, compared with theoretical calculations, advance our understanding of quantum Hall physics in graphene-based devices. The study undoubtedly offers valuable insights for a broad audience, especially for those focused on graphene allotropes and quantum Hall physics. Consequently, I believe this study would be a fitting contribution to *Nature Communications*.

However, I recommend addressing a few points to sharpen the focus on the main findings and avoid possible misinterpretations of the data. The paper could have greater impact by further developing certain aspects, such as the characterization of the stacking order and the role of intertwined cubic and parabolic dispersions in the tunability of the quantum Hall phase diagram. I therefore ask the authors to answer the following questions and revise the manuscript accordingly, to make it clearer and more relevant to the community:

Reply: We thank the reviewer for this very positive feedback and for the enthusiastic recommendation of publication. Below we provide detailed answers to the specific questions.

Stacking order identification

The identification of ABCBC stacking is not entirely clear. In Extended Data Figure 1, the authors state: "The brightest area is tentatively identified as a metastable stacking (e.g., ABCBC), though the exact structure remains uncertain from this characterization alone."

However, this issue does not appear to be solved in the rest of the supplementary information, and it is unclear which measurement can unambiguously discriminate among metastable orders.

Reply: We thank the reviewer for this important comment. The unambiguous identification of the ABCBC stacking order has indeed been clarified in our separate manuscript entitled "Intrinsic layer polarization and multi-flatband transport in non-centrosymmetric mixed-stacked multilayer graphene" [arXiv:2505.12478], which studies the same device as that used in the present manuscript. We have confirmed that the stacking order of the devices understudy is ABCBC through electrical transport, NanoARPES and band-structure analysis.

In brief, the stacking order is firstly confirmed by the transport measurement, as summarized in Fig. R12. The R_{xx}-n-D map at B = 0 T of the same device with this article

exhibits pronounced asymmetry between $\pm|D|$, with metallic behavior at $+|D|$ and nonmonotonic insulating behavior at $-|D|$. Such asymmetry can only arise in non-centrosymmetric pentalayer polytypes. Among all six possible stackings (ABABA, ABCAB, ABACA, ABCBA, ABCAC, ABCBC), only ABCAC and ABCBC exhibit non-centrosymmetry. We further compared the experimentally extracted transport gap versus interlayer potential difference Δ with tight-binding calculations. The experimental gap reopens and increases at large negative D, matching the calculated behavior of ABCBC, while ABCAC predicts gap closure at $\Delta < -18$ meV, inconsistent with experiment. Thus, transport data directly confirm the ABCBC order.

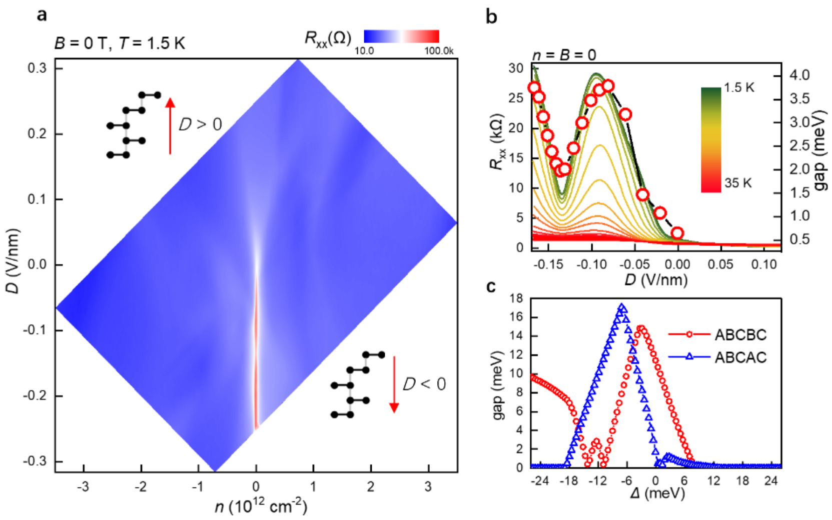

Fig. R12. Stacking identification of ABCBC through transport. a, Color plot of resistance R_{xx} as a function of carrier density n and displacement field D. The color bar is in the log scale. **b**, D-dependent R_{xx} for $n = 0$ at varied temperatures from 1.5 K to 35 K, the red hollow circles are the corresponding transport gap extracted from Arrhenius plot. **c**, Band gap with dependence of interlayer potential difference Δ according to tight-binding calculation. Data shown in **a** and **b** is the same device in the main text.

To further confirm the stacking order, we did NanoARPES measurement on another device named as M4, its measured dispersive image can be well fitted by the calculated band structure of ABCBC but not ABCAC (Fig. R13 a, b, c), this device was then covered with top hBN and top gate, and measured by transport. As shown in Fig. R13d, the measured R_{xx}-n-D color plot is similar with the device in our main text (Fig. R12a), in which, at $+|D|$ side, the resistance is relatively small, at $-|D|$ side, resistance increases first and then decreases, but finally it would re-increase.

As a conclusion, by combining the transport data, NanoARPES data, and calculated band structures, we conclude that the stacking order of the devices in this work is ABCBC.

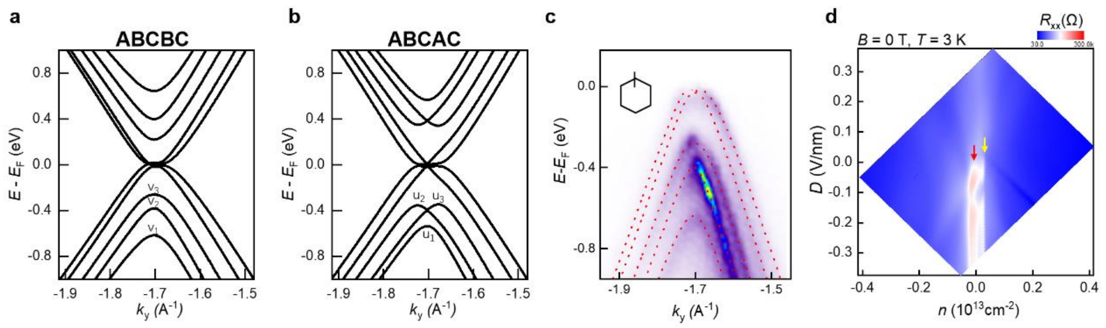

Fig. R13. NanoARPES measurement of device M4. a and **b**, Calculated single particle band structures of ABCBC and ABCAC, respectively. **c**, Electronic band structure attained from NanoARPES measurement along Γ-K direction (vertical line in the inset). The red dotted lines are the calculated band structure of ABCBC, it can well fit the dispersion image measured from NanoARPES. **d**, n-D color plots of longitudinal resistance for M4. At CNP, there shows nonmonotonic D dependence similarly with that in Fig. R12a. It should be mentioned that in the color plot there shows two peaks, marked by red and yellow arrows in **d**, it suggests there are two domains with different intrinsic doping along source and drain.

In the Methods section, it is stated that scanning near-field optical microscopy (SNOM) is used, but Extended Data Figure 1 shows near-field infrared, and Extended Data Figure 2 shows phonon-polariton assisted near-field scanning microscopy. Are these all considered part of the SNOM method?

Reply: We thank the referee for raising this question. Both Extended Data Fig. 1 and 2 are obtained using scattering-type near-field optical microscopy (s-SNOM). The difference lies only in the incident light wavelength used for different device configurations:

1. For uncovered graphene on SiO_2, we used λ = 10.6 μm (1110 cm^{-1}, CO_2 laser), where the near-field signal is directly generated on the graphene surface.

2. After encapsulation by hBN, the excitation wavelength was changed to λ = 6.3 μm (1587 cm^{-1}, quantum cascade laser), within the Reststrahlen band of hBN (746–819 cm^{-1} and 1370–1610 cm^{-1} illustrated in Fig. R14a). This enables phonon–polariton-assisted near-field imaging, allowing infrared light to couple through the hBN and interact with the underlying graphene layers (illustrated in Fig. R14b).

The 10.6 μm light cannot excite phonon–polaritons and is strongly screened by hBN, while the 6.3 μm excitation yields strong contrast, as shown in Fig. R14c–e. This approach follows our previously established methodology (see Nat. Nanotechnol. 19, 188 (2024)). Therefore, both figures are acquired using SNOM, with or without phonon–polariton assistance depending on the excitation wavelength.

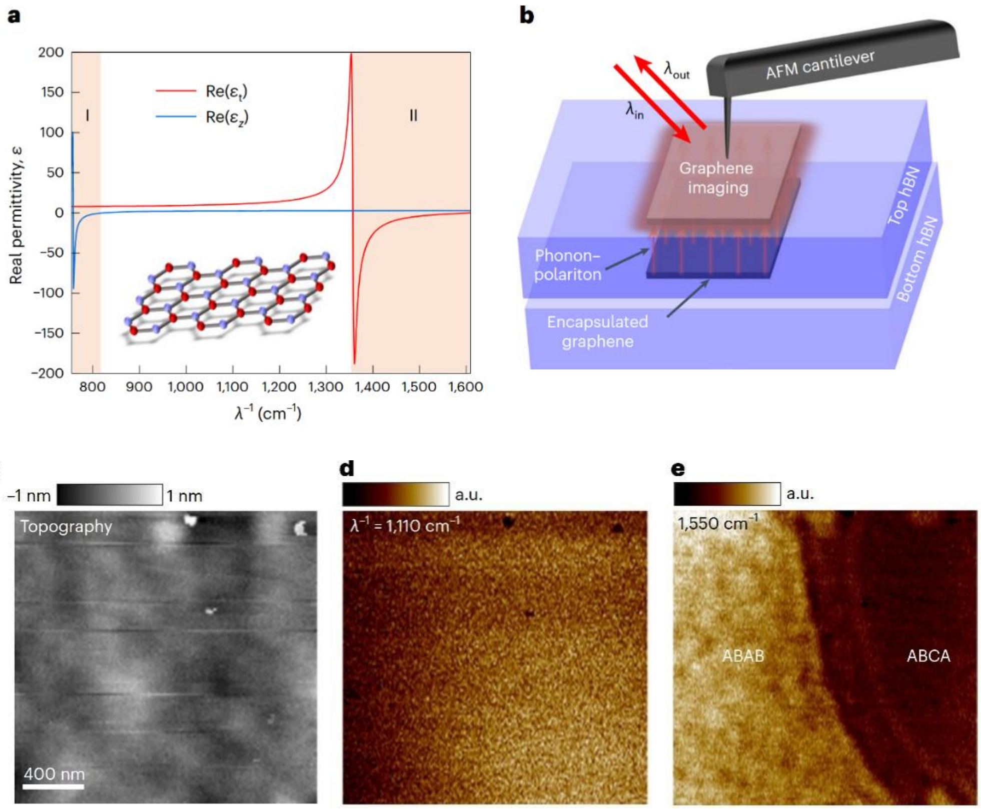

Fig. R14. Phonon–polariton-assisted near-field optical imaging of graphene stacking orders under hBN coverage. a, Derived real part of the permittivity tensor components of hBN. Type-I lower (ω = 746-819 cm^{-1}) and type-II upper Reststrahlen bands (ω = 1370-1610 cm^{-1}) are shaded. The inset shows a schematic of the hBN crystal structure. **b**, Illustration of the phonon-polariton-assisted near-field optical imaging measurement of graphene encapsulated by hBN. Infrared light (red arrows) at a certain wavenumber within the hBN Reststrahlen band is focused onto the apex of a metal-coated AFM tip. The induced phonon–polaritons (orange arrows) are reflected by graphene and propagate onto the top hBN surface, then they are scattered by the AFM tip and detected by an mercury cadmium telluride detector in the far field. **c – e**, Topography (**c**) and near-field images when incident wavenumbers (λ^{-1}) are 1,110 cm^{-1} (**d**) and 1,550 cm^{-1} (**e**). In **d**, there is no contrast, but there show obvious ABCA and ABAB domains in **e**, since 1110 cm^{-1} is beyond the Reststrahlen bands but 1550 cm^{-1} is within Reststrahlen bands.

Please label the scale bars in Extended Data Figures 1d and 2c so that we better understand what is measured exactly.

Reply: We thank the referee for pointing out this problem. We have added the scale bars accordingly (Fig. R15&16).

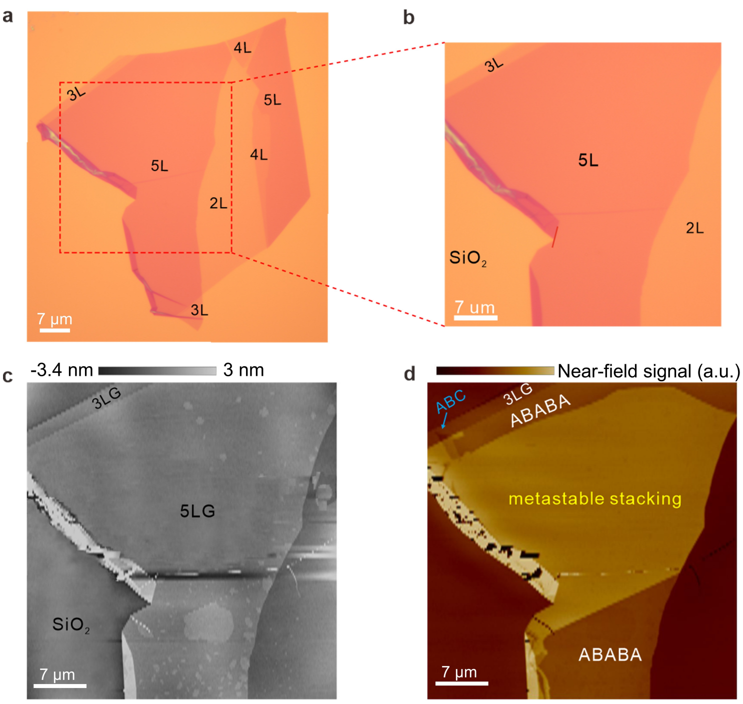

Fig. R15. Revised Extended Data Fig. 1. Scale bars are added to all the figures.

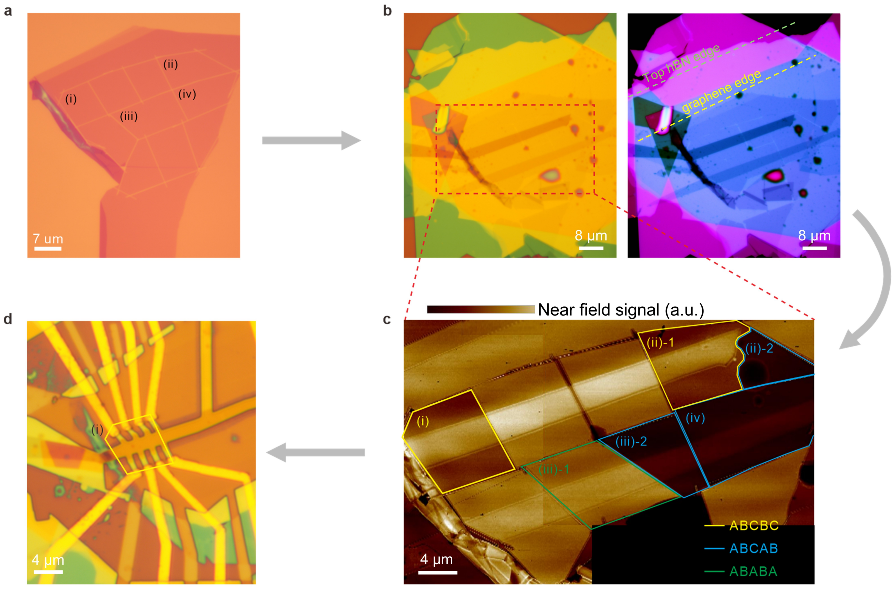

Fig. R16. Revised Extended Data Fig. 2. Scale bars are added to all the figures.

Is the final identification of the stacking order based on transport measurements?

Reply: We thank the reviewer for further concern about stacking order identification. As answered above, the stacking is identified by both transport and NanoARPES measurement.

I recommend adding a section titled **"Identification of the stacking order"** to make these

points transparent and clear, since it is central to the paper.

Reply: We thank the reviewer for this important suggestion. We have added “Identification of the stacking order” in the Methods part, it is also shown below:

“Identification of the stacking order

The stacking order of the device was unambiguously determined to be ABCBC, consistent with our previous study (arXiv:2505.12478), based on the characteristic asymmetric displacement-field dependence of the transport gap and NanoARPES band dispersions that uniquely agree with the calculated electronic structure of the ABCBC stacking. The device investigated in the main text is the same one characterized in that study.”

Technical details

The use of the Si gate is mentioned to improve electrical contacts to the device. Please provide the value of the applied Si gate voltage in method, at least for the measurements in Main Text Figure 1. Please also comment on why the electron side (positive filling factor) is not studied at high field in the paper. If this is due to contact issues, please clarify.

Reply: We thank the reviewer for the careful reading of our manuscript. The applied Si gate voltage is –(35~45) V when we did measurement at hole side. We didn’t discuss about the electron side since the measured signal is not clear just due to the contact although we set Si gate to +(49~60) V, most regions are not very clear due to bad contact and some regions are obsured by resistive states. We only observe some odd-denominator FQH states shown in Fig. R17a and b.

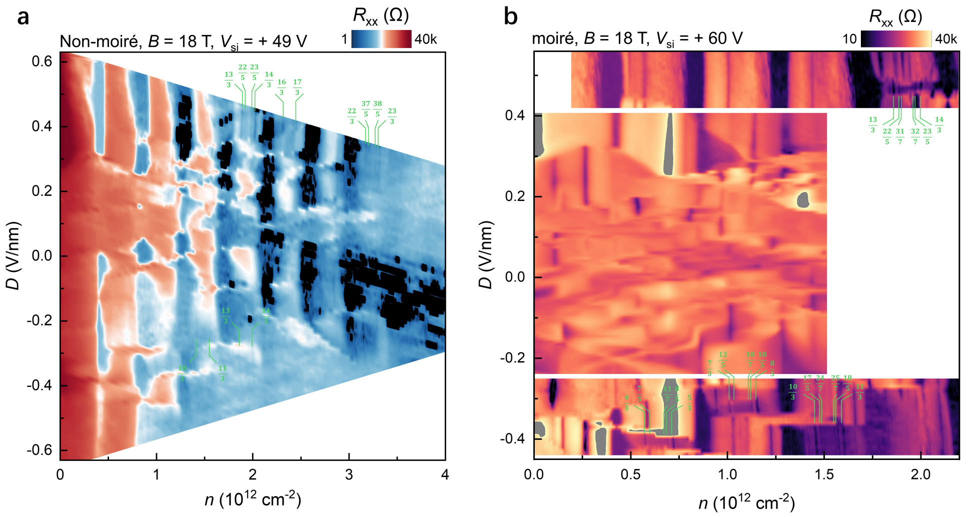

Fig. R17. Fractional quantum Hall states on electron side. a and **b**, R_{xx}-n-D color plot at B = 18 T, T = 14 mK for the non-moiré and moiré devices, respectively. Silicon back gates were biased at +49 V (non-moiré) and +60 V (moiré) to reduce the large contact resistance arising from p–n junctions. The signal quality on the electron side is limited, and fractional quantum Hall states are generally unresolved in most regions for both devices. Only several odd-denominator FQH states are discernible, highlighted by green lines.

Since graphite gate screening is crucial for understanding the effective Coulomb interaction and because it affects the fractional quantum Hall energy gaps, please provide the hBN thickness used in this device.

Reply: We thank the reviewer for the suggestion. The thickness of the top and bottom is about 47 nm and 42 nm, respectively. In the supplementary information, when we consider the interaction between different bands, we actually take these parameters into account (supplementary: "At this field, the magnetic length is l_B=6.05 nm, d=14.91l_B, and the energy scale…", d is defined as the distance between the top and bottom graphite gates). And we have added the thickness in the main text.

We have added one sentence in line #400: "The thickness of top and bottom hBN is 47 nm and 42 nm, respectively, for the device in the main text."

Abstract statement

The sentence "However, experimental platforms simultaneously offering large energy gaps, delicate tunability, and robust non-Abelian signatures remain scarce" is misleading, as the paper does not directly address these issues. Pentalayer graphene should simply be presented as a novel platform to study even-denominator fractional quantum Hall states.

Reply: We appreciate the referee's insightful comment. We agree that our work does not directly address large energy gaps or non-Abelian signatures, and we have therefore revised the sentence to more accurately describe the motivation and scope of this study. Our focus is on the tunability and multiband character of mixed-stacked multilayer graphene as a new platform for exploring even-denominator FQH states. The revised sentence now reads:

Line #30: "However, experimental platforms simultaneously offering delicate tunability and multiband conditions conducive to even-denominator fractional quantum Hall states remain scarce."

Intertwined quadratic and cubic band dispersions

The band structure is emphasized twice:

"…a previously unexplored system characterized by intertwined quadratic and cubic band dispersions."

"…its band structure combines cubic and parabolic dispersions near the Fermi level with a small intrinsic gap (Fig. 1c, left), and undergoes a relative energy shift between the cubic and parabolic bands under external perpendicular displacement field D (Fig. 1c, right)."

It would be helpful to better explain the consequences of this feature for quantum Hall physics. Does it provide a tunable parameter, absent for example in bilayer graphene.

Reply: We thank the referee for this insightful comment. The intertwined parabolic and cubic dispersions are indeed a key feature of mixed-stacked pentalayer graphene, and they play a

central role in determining its quantum Hall behavior.

(1) Tunable hybridization driven by displacement field. As shown in Fig. R18i-iii, the electronic structure of the ABCBC stacking can be viewed as a hybridization between a parabolic band originating from the Bernal (AB) layers and a cubic band from the rhombohedral (ABC) layers. At low energy, these two sub-bands strongly hybridize, so that neither remains purely parabolic nor purely cubic. The application of a perpendicular displacement field D shifts the relative energy between the two sub-bands and continuously tunes their hybridization strength. This field-driven intertwinement provides an additional tunable degree of freedom that is absent in conventional bilayer graphene. This tunability directly affects the quantum Hall phase diagram. The Landau levels (LLs) near charge neutrality consist of mixed contributions from both the AB-like and ABC-like components. As illustrated in Fig. R19, the average occupation of the AB (parabolic) and ABC (cubic) layers within each LL varies systematically with D, indicating that the degree of hybridization is electrically controllable. The interaction between electrons residing in these intertwined bands is therefore tunable by D, which in turn modifies the effective Landau level mixing and can determine which type of Moore–Read–like even-denominator state is energetically favored.

(2) Interaction effects between intertwined bands. The electron-electron interactions between two intertwined bands are tunable driven by displacement field. Unlike pure Bernal bilayer graphene, which hosts a single low-energy band, the ABCBC structure retains two energetically close bands whose mutual interactions cannot be neglected (Fig. R18iii). The displacement field modifies these interband interactions by shifting and intertwining the two bands, thereby tuning the correlated ground states. Similar displacement-field–driven control of interband coupling has been observed in other moiré and multilayer graphene systems—for example, in twisted trilayer graphene where tuning between a flat band and a Dirac band enhances superconductivity (Nature 590, 249–255 (2021)).

(3) Landau level mixing (LLM). The intertwined multiband structure gives rise to a series of Landau level mixing, which breaks particle–hole symmetry and determines whether the Pfaffian or anti-Pfaffian phase is favored at half filling (Sodemann & MacDonald, PRB 87, 245425 (2013); Rezayi, PRL 119, 026801 (2017)). Such LLM-induced effective three-body interactions are known to stabilize non-Abelian states (Peterson et al., PRL 113, 086401 (2014)), and in our system, the strong multiband hybridization likely amplifies these effects.

In conclusion, compared with pure Bernal bilayer graphene, the intertwined band structure offers new freedoms, i.e., the tunable inter-band (layer) hybridization, interactions, LLM driven by displacement field. And we rewrote one sentence in the main text to highlight consequences of intertwined band structure.

The original sentence in line #89: "…with distinct orbital components, enabling LL crossings under electric or magnetic fields[18,37]."

Rewritten sentence in line #92: "···with distinct orbital components, enabling tunable LL crossings, hybridizations and interactions between different bands and layers under electric or magnetic fields[22,31,44]."

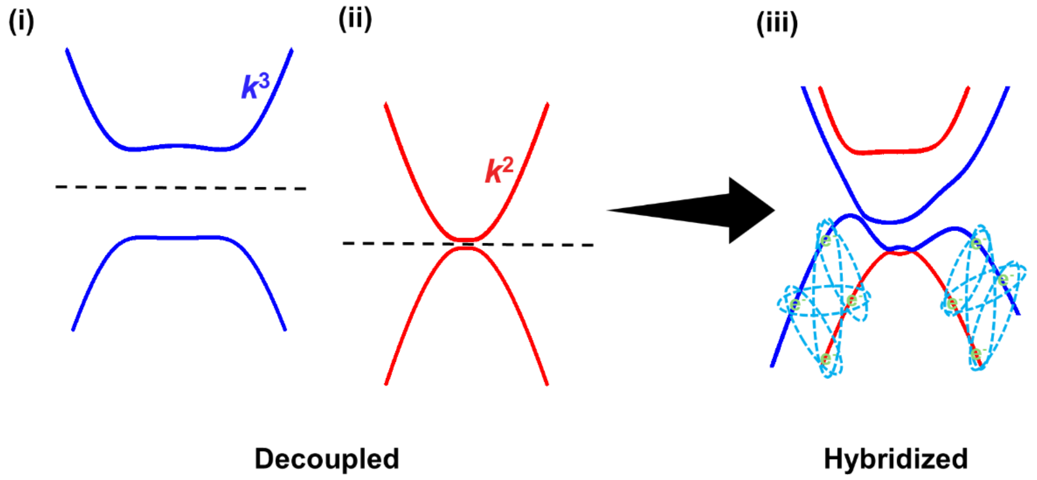

Fig. R18. Illustration of ABABC band structure under *D* < 0. The band of ABCBC is composed by a cubic band **(i)** and a parabolic band **(ii)**, the hybridization between them modifies the low energy band structure **(iii)**. The light blue dashed circles represent inter-band interactions. The electron-electron interactions between them are important to decide the correlated ground states.

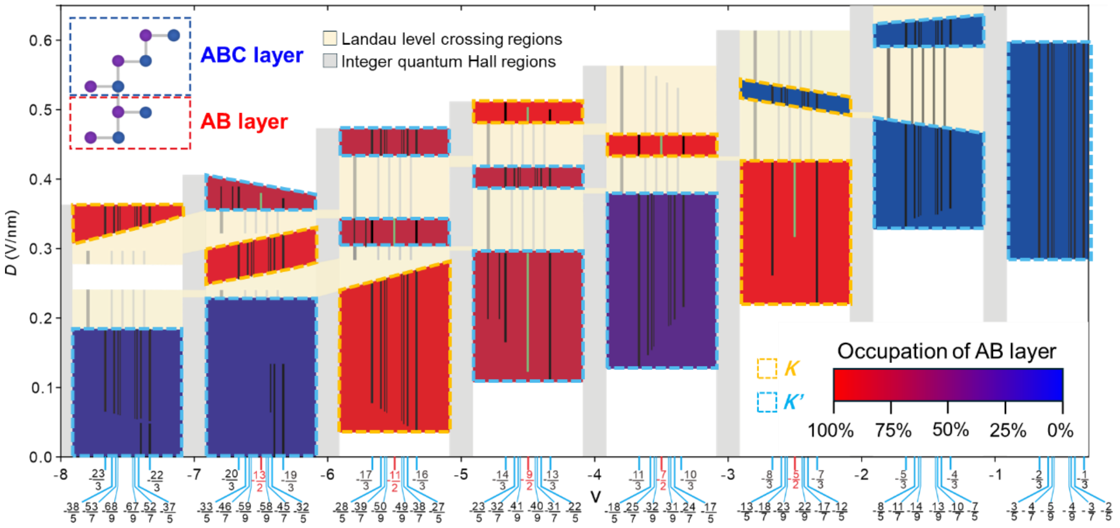

Fig. R19. Schematic phase diagram labeling the ground-state order for the same filling fraction range as shown in Fig.1 d of the main text. The light blue- and dark yellow-dashed lines enclosed areas are occupied by broken-symmetry states with valley index *K* and *K'*, respectively, whereas the color-filled regions denote the occupation of AB layer. If the Landau level is 100% occupied by AB layer, the region is filled by red, if 0% occupied, it is filled by blue. Even-denominator regions around filling factor -9/2 (higher *D*), -7/2, -5/2 exhibit ≈93.8%

occupied with AB layer, whereas those around -13/2, -11/2, -9/2 (lower *D*) exhibit ≈75.1% AB-layer occupation.

In Supplementary Fig. S1, some bands are pushed far in energy and do not contribute to the low-energy quantum Hall phase. Could the authors explicitly indicate which bands correspond to cubic or parabolic dispersion? Does these intertwined dispersions play a role in the high field quantum Hall phase diagram?

Reply: We thank the reviewer for this helpful suggestion. In the revised manuscript, we have updated Supplementary Fig. S1 (as shown in Fig. R20) to explicitly indicate which bands correspond to cubic or parabolic dispersion. As shown in the band spectra presented in Figs. S6, S8, S10, S12, and S14, the influence of adjacent higher-energy Landau levels on the even-denominator states is very weak. Therefore, the dominant physics primarily arises from the lowest-energy LL, while the higher-energy LLs play an even less significant role in determining the observed fractional quantum Hall features.

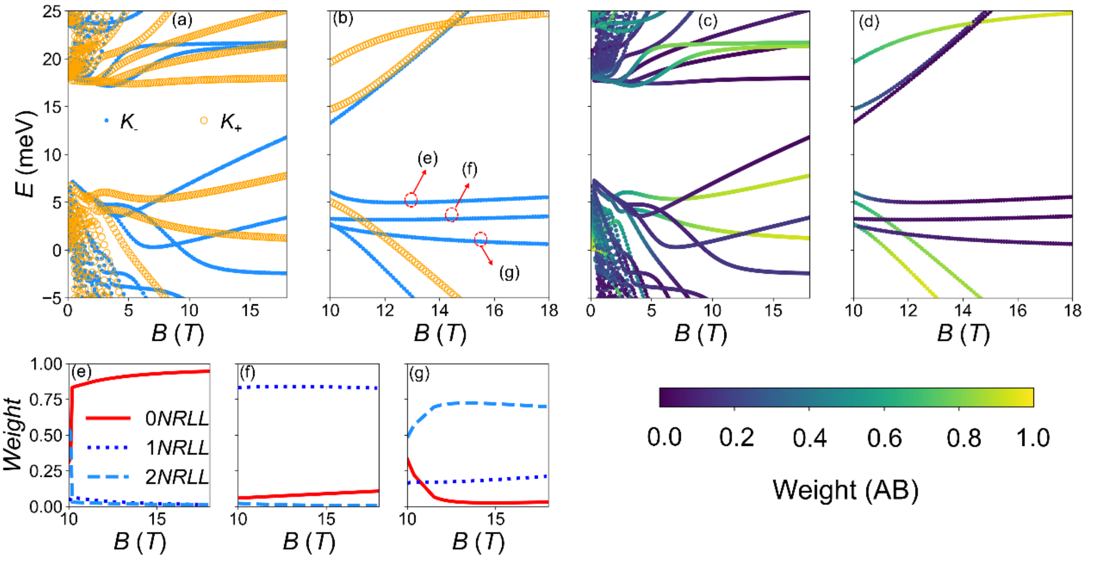

Fig. R20. Revised Fig. S1. **a**, LL diagram at U = 0, Blue dots denote Landau levels in the K_- valley, while orange circles indicate those in the K_+ valley. **b**, LL diagram at U = -25 meV. **c**, Contribution from the AB-stacked component to each LL at U = 0. **d**, Contribution from the AB-stacked component to each LL at U = -25 meV. **e**, **f**, **g** show the weights of the 0NRLL, 1NRLL, and 2NRLL components, respectively, for the LLs marked in red in panel **b**.

Landau level mixing

The manuscript states: "Unlike simpler graphene systems, the multiband nature of ABCBC-5LG amplifies LL mixing effects, which are critical for stabilizing correlated states at fractional fillings."

Please elaborate. In what sense does LL mixing stabilize correlated states? LL mixing is often expected to increase screening of the Coulomb interaction, which generally reduces the energy gap of fractional quantum Hall states.

Reply: We thank the reviewer for this insightful comment. The term "stabilize" in our statement does not refer to an enhancement of the excitation gap. Rather, it refers to the selection of a particular topological ground state among several competing candidates due to LLM.

As discussed in the manuscript, LLM breaks particle–hole symmetry, which can energetically favor either the Pfaffian or anti-Pfaffian phase at half filling, depending on the strength and nature of the mixing. From the perturbation calculation (I. Sodemann and A.H.MacDonald, Phys. Rev. B 87, 245425(2013) and E. H. Rezayi, Phys. Rev. Lett. 119, 026801 (2017)), the LLM could induce effect three-body interaction between electrons which is crucial to stablize the non-Abelian FQH state at half filling. In monolayer graphene, for example, moderate LLM in the N = 1 Landau level has been shown to stabilize the anti-Pfaffian phase (M. R. Peterson et al., Phys. Rev. Lett. 113, 086401 (2014)). In our system, the multiband nature of ABCAC-stacked 5-layer graphene enhances LLM effects, which in turn plays a crucial role in determining which Moore–Read–type state is realized.

In the revised manuscript, to avoid ambiguity, we have revised the sentence:

"… the multiband nature of ABCBC-5LG amplifies LL mixing effects, which governs the stability and character of fractional quantum Hall states."

Robustness and Unprecedented tunability claims

The claim of "unprecedented displacement-field tunability driven by LL crossings in the hybridized multiband structure" is underdeveloped. Previous multilayer graphene studies have already shown displacement-field tunability and transitions between even-denominator and Jain states. Please revise or develop this statement.

Reply: We thank for the careful reading of our manuscript. We have revised the sentence:

Original sentence: "…exhibit unprecedented displacement-field tunability driven by LL crossings in the hybridized multiband structure."
Now in line #38: "…exhibit displacement-field tunability associated with the hybridized multiband structure in the ZLL."

The manuscript states: "the highest half-filling even-denominator FQH states in ZLL reported so far, indicating the robustness afforded by the multilayer graphene platform." This argument is misleading: robustness should be characterized by the energy gap, which in this paper appears much smaller than in Bernal bilayer graphene even-denominator states. Please clarify why the highest filling factor should be seen as an advantage or remove the statement about robustness.

Reply: We thank the referee for this thoughtful comment. We agree that the energy gap is

the proper measure of robustness, and our intention was not to imply that the observed even-denominator states in mixed-stacked pentalayer graphene exhibit larger gaps than those in Bernal bilayer graphene. Rather, we intended to highlight that such even-denominator FQH states persist up to unusually high filling factors (e.g., $\nu = -11/2$ and $-13/2$) within the zeroth Landau level, demonstrating that the correlated states can survive in higher-index Landau levels of this multiband system—an aspect not previously observed.

To avoid any confusion, we have revised the sentence to remove the implication of enhanced robustness. The revised text now reads:

Line #185: "It is remarkable that we observe even-denominator FQH states up to $\nu = -11/2$ and $-13/2$—the highest half-filled states reported so far in the zeroth Landau level—highlighting the extended stability of such states across multiple intra-ZLLs in mixed-stacked multilayer graphene."

This modification removes the reference to "robustness" and clarifies that the significance lies in the extent of the observed sequence, rather than the magnitude of the energy gaps.

Comparison with similar graphene devices

While the article refers to an extensive body of literature on quantum Hall physics, many of the cited works concern earlier-generation graphene devices, which are not easily comparable to the state-of-the-art device quality studied here (i.e., hBN encapsulation and dual graphite gates). More recent studies focusing on fractional quantum Hall physics in graphene devices of similar quality to the one reported in this paper would be relevant. Phys. Rev. X **12**, 031019 (2022), Phys. Rev. Lett. **132**, 046603 (2024), *Nat Commun* **16**, 7255 (2025).

Reply: We thank the reviewer for the helpful suggestion. We have added these articles to references, which are numbered as Ref [28], [29], [30] in the main text.

CF effective mass

Screening and disorder can reduce the measured gap of fractional quantum Hall states (as shown again very recently *Nature* **644**, 646–651 2025), therefore I find that the comparison of the effective mass to GaAs CF state is not relevant : «comparable to values previously reported in GaAs» . A comparison of the effective mass to other dual gated hBN encapsulated graphene devices would be more relevant.

Reply: We thank the reviewer for this comment. We agree that comparing the effective mass to GaAs composite fermion states is not directly meaningful due to the influence of screening and disorder. We have therefore removed this comparison and instead discuss the effective mass in the context of other dual-gated, hBN-encapsulated graphene devices [Physical Review Letters 121, 226801 (2018)]. This highlights that our results are consistent with previously reported high-quality graphene measurements. The revised text now reads:

Line #160: "comparable to values previously reported in monolayer graphene[Ref] [Ref: H. Polshyn, et al., Physical Review Letters 121, 226801 (2018)]"

Phase transitions at half fillings

The manuscript states: "At half fillings, continuous quasiparticle phase transitions between paired FQH states, magnetic Bloch states, and composite Fermi liquids are clearly identified upon tuning external fields."

In fact, quasiparticle properties are not addressed experimentally. Figure 4 shows transitions, but the sharpness of these appear simply due to the presence or absence of an energy gap. This figure could be moved to the Supplementary Information. If it remains in the main text, the authors should better explain its intended message and revise the corresponding discussion

Taken has it is, expanding the discussion on the observed daughter states of the even-denominator states, mentionned at the end of the previous paragraph, would be more relevant than the discussion about phase transition, as this paper focus on the « cascade of

Reply: We thank the reviewer for this thoughtful comment. We agree that our original phrasing "quasiparticle phase transitions" may have been misleading. We have revised the wording in the Abstract to clarify that the observed features represent transitions between distinct quantum phases rather than direct measurements of quasiparticle properties.

We revised in line #40: "At half fillings, continuous quantum phase transitions between **even-denominator fractional quantum Hall states**, magnetic Bloch states, and composite Fermi liquids are clearly identified upon tuning external fields."

We added another sentence in line #236: "We emphasize that these identifications are based on transport signatures; direct probes of the underlying quasiparticles are left for future work."

We think the presence or absence of an energy gap is not the main factor to cause the sharpness. One reason is the resistance at ν = -7/2, -5/2, which already enters into incompressible state, is even bigger than that in CFL state, thus, the sharpness is not mainly caused by the drop after entering into the incompressible FQHs. Another reason is that we also did linecuts at other filling factors. In the vicinity of -5/2, we have three other linecuts shown in Fig. R21b-d, the black and grey dashed lines correspond to the phase boundary indicated in Fig. R21a by the same-colored dashed lines. It is obvious that resistance change at two sides of black lines is much bigger than those at two sides of grey lines. For example, at ν = -2.73, the resistance change from B = 11.55 T to 11.85 T is 456.3 Ω, but the change from 17.25 T to 16.95 T is 4083.4 Ω. It shows similar results around ν = -7/2 (Fig. R21e-h).

Such a distinct difference may be explained by the different charge distribution at different regions. From the calculated phase diagram shown in Fig. R22a, even- (labeled as I

in Fig. R22a) and odd- (labeled as II) denominator FQHs both occupy *K* valley. Even-denominator regions around -7/2 and -5/2 fill the landau levels with AB layer occupation both over 90%, while the odd-denominator regions fill the landau levels with AB layer occupation both less than 1%. Since the Hofstadter effect in the landau level crossing regions (labeled as III) is pronounced, it may suggest the carriers are in the moiré layer. Identified from the optical image of the heterostructure (Fig. R22c), since top hBN almost aligns with ABCBC, moiré layer is likely to be in the top layer. Accordingly, the charge transfer process is illustrated in Fig. R22c. From region I (III) to region II, the carriers are transferred from AB (ABC) layer to the topmost moiré layer. For the latter, the charge transfer process is relatively continuous since before and after the transfer the charge may only move one layer, but for the charge transfer from I to II, the charge should move from bottom layers to top layers, it may be a sudden process and give rise to the sharp boundary between I and II.

We agree to remove original Fig. 4b and c since we don't directly detect the quasiparticles while we want to reserve Fig. 4a and add Fig. R21b-d and Fig. R22c, in such a way: (1) We can gain more insight into the ground states of the even-denominator FQHs, which may be polarized into AB part. (2) We show continuous transition between even-denominator FQHs, Hofstadter states and odd-denominator FQHs, which provides key physical insight into how external tuning parameters govern the interplay between pairing, band structure, interaction and lattice effect—an essential aspect of our study. (3) Moiré effect is further explained as a weak perturbation for the even-denominator FQHs, only observable at Landau level crossing regions.

We have exchanged original Fig. 4 b&c into Fig. R21b-d and Fig. R22c, and added Extended Data Fig. 9 and discussion about them from line #238: "Interestingly, we observe a qualitative difference from the transport behaviors between these phase transitions. Figure 4b shows linecuts of Fig. 4a at ν = -2.73, -8/3, -2.4 along the magnetic field, where the transition from region III (LL exhibiting odd-denominator FQH, blue shaded) to region II (Hofstadter minibands, yellow shaded) appears relatively gradual, with a slow variation in R_{xx} across the transition. In contrast, the transition from region I (LL exhibiting even-denominator FQH, pink shaded) to region II is significantly sharp, as indicated by a steep change in R_{xx}. Transitions around ν = -7/2 also shows similar behavior (Extended Data Fig. 9b-e). The comparison suggests that the two transitions may be captured by distinct quantum phase transitions. The single-particle calculated results show that orbitals in I and III share the same valley, while I is occupied by carriers in AB layer but III in ABC layer. Region II is dominated by Hofsdtater minibands, suggesting carriers are in the topmost layer since the moiré lattice is formed between top hBN and graphene (Extended Data Fig. 2b). As illustrated in Fig. 4c, the different transition sharpness may reflect the distinct charge transfer process across the transitions. The underlying mechanism deserves further investigations."

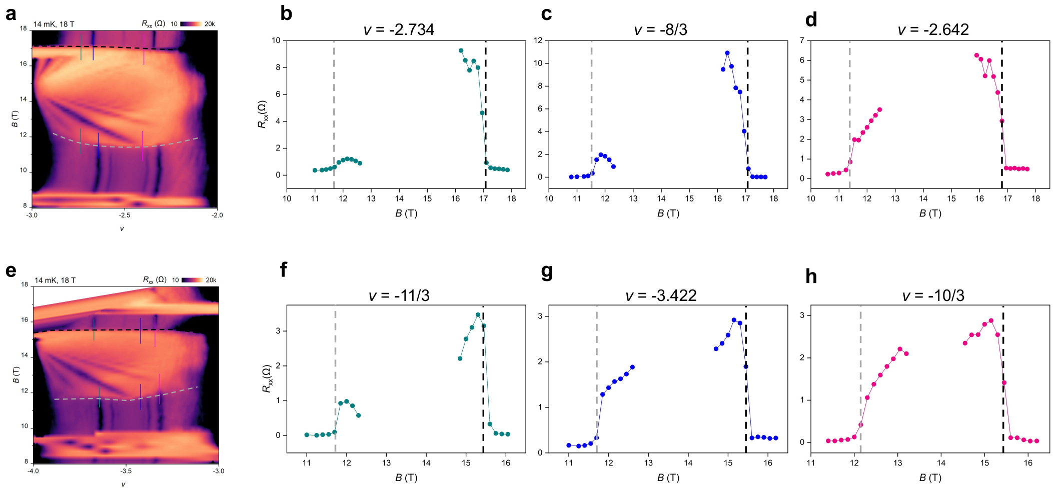

Fig. R21. Comparison of transitions at different fillings. a and **e,** R_{xx}-ν-B color plots around ν = -5/2 and -7/2. The black and grey dashed lines are used to mark the phase boundary from odd-denominator FQHs regions to Landau level crossing regions, and the Landau level crossing regions to even-denominator FQHs regions, respectively. **b-d and f-h** are different linecuts of R_{xx} along the green, blue and pink lines in **a** and **e**.

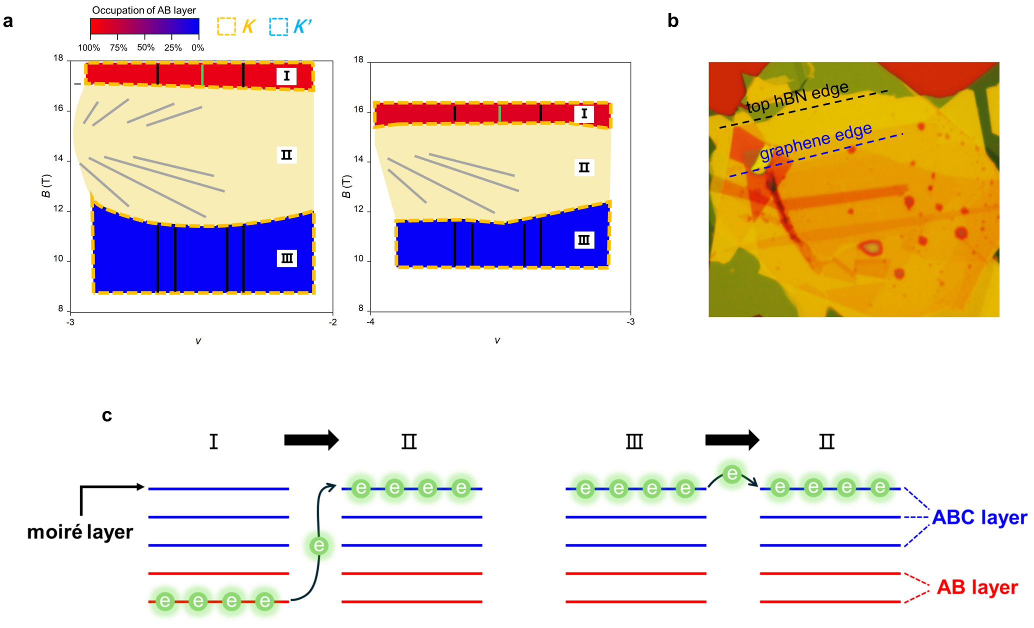

Fig. R22. ***B*** **dependent Phase transitions. a**, Schematic phase diagram around ν = -5/2 and -7/2. **b**, optical image of the device in the main text, the edges of graphene and top hBN almost align with each other. **c**, Charge distributions for different quantum hall phases and transition processes between them.

Following the referee's suggestion, we have also added the discussion of the daughter states in the third paragraph of "Two groups of even-denominator FQHs", emphasizing their

hierarchy and relationship to the parent states, which support the central theme of the "cascade of even-denominator states."

We wrote from line #203: "Theoretical analyses [Phys. Rev. B 79, 205301 (2009)] have proposed a hierarchy of daughter states arising from e/4 excitations of the anti-Pfaffian phase, with the simplest ones predicted at ν = 6/13 and 9/17. Shown in Extended Data Fig. 8, weak R_{xx} dips at ν = -2-9/17, -4-6/13, -4-9/17 are observed in the vicinity of -5/2 and -9/2, suggesting the possible existence of anti-Pfaffian ground state. We note the weak signals may be limited by the sample quality and contact issue. We caution that the identification of paired states based on their daughters is indirect and has not been independently confirmed by direct measurements, such as thermal conductance [Nature 559, 205 (2018)] or upstream noise at interfaces [Science 375, 193 (2022)]."

Reviewer #1 (Remarks to the Author):

The authors addressed all of my questions in detail. They did not perform in-plane measurements, which is understandable given their instrumentation.

Reply: We sincerely thank the reviewer for the careful evaluation of our manuscript and for the constructive questions throughout the review process. We appreciate the reviewer's understanding regarding the current experimental limitations.

One final question concerns Fig. R1. It appears that the even-denominator states originate primarily from the AB region. In GaAs and bilayer graphene studies, even-denominator formation is typically observed mainly in orbital 1 rather than orbital 0. Is the situation similar here?

Reply: We thank the reviewer for this insightful comment. Our observation is consistent with GaAs and bilayer graphene studies.

In conventional two-dimensional electron gas, such as GaAs, it is well established the wavefunction of its $N = 1$ Landau level (here we name it as nonrelativistic first Landau level (1NRLL)) can stabilize Moore–Read–type state (*Phys. Rev. Lett.* **59**, 1776 (1987); *Nuclear Physics B* ***360***, 362 (1991)). However, in bilayer graphene, its $N = 1$ LL has an unusual composition, comprising a mixture of the conventional Landau orbital 0 (0NRLL) and 1 (1NRLL) wave functions, but 1NRLL is dominant so that Moore–Read–type state can be stabilized in $N = 1$ (See Jia Li *et al* in *Science* **358**, 648–652 (2017)). Therefore, the key requirement is not simply the Landau level index, but rather the dominance of the 1NRLL orbital character in the wavefunction.

In our ABCBC system, the band structure cannot be strictly decomposed into independent ABC and AB stacking components since we have to consider the interlayer hoppings, especially in the presence of an applied external electric field. As a result, assigning a pure $N = 0$ or $N = 1$ character based solely on layer origin is not appropriate. However, in the supplementary, we calculate the occupation of 1NRLL in each Landau level, and we found the wavefunction of 1NRLL is actually dominant (>70%) in all our observed even-denominator FQHs (marked by ① and ② with red circles in Fig. 1c).

Therefore, these states appear predominantly in the AB layer and their orbital structure is in fact 1NRLL-dominated. In this sense, our observations are fully consistent with previous findings in GaAs and bilayer graphene systems.

To further clarify this point, we have modified the manuscript accordingly. In the previous version, we wrote in line #191: "The results reveal two distinct valence-band LLs with particularly large contributions from the $N = 1$ LL (see SM) ⋯", here, "$N = 1$" represents the $N = 1$ LL in two-dimensional gas, not that in ABCBC. To avoid confusion, we have revised the sentence in line #191 to: "The results reveal two distinct valence-band LLs with particularly large **occupation by the same wavefunction with $N = 1$ Landau level in conventional two-dimensional electron gas, which is well established to stabilize Moore-Read state**[3,8] (see SM) ⋯".

In addition, we have removed "where a single LL branch (orbital $N = 0$) is active" in line #148 to avoid misinterpretation. Our original intention was to indicate $N = 0$ (0NRLL in two-dimensional electron gas) is dominant in those fillings, which is consistent with the

observation of only odd-denominator states.

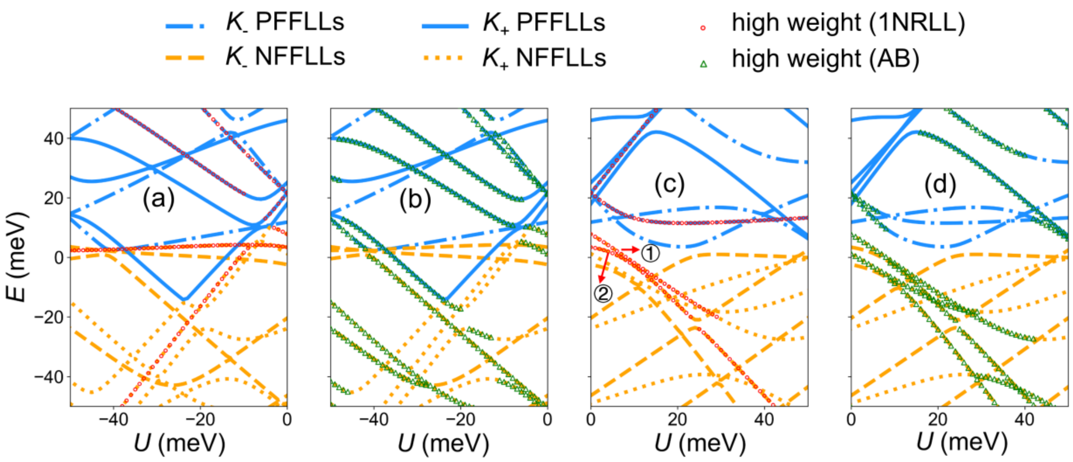

Fig. 1 | Energy bands of the ABCBC-stacked pentalayer graphene. The blue dashed lines denote positive filling factor's Landau level's (PFFLLs) in the K_- valley, while the blue solid lines represent those in the K_+ valley. The orange dashed lines indicate negative filling factor's Landau level's (NFFLLs) in the K_- valley, the orange dotted lines correspond to those in the K_+ valley. Landau levels marked with red circles possess a 1NRLL weight exceeding 70%, and the green hollow circles indicate states with more than 50% contribution from the bilayer AB-stacked configuration. (a) $U < 0$. (b) $U < 0$. (c) $U > 0$. (d) $U > 0$. Here, $U > 0$ ($U < 0$) corresponds to $D > 0$ ($D < 0$) in the experiment.

Reviewer #3 (Remarks to the Author):

Thank you to the authors for addressing the questions and revising the manuscript. I have no further comments. The responses clarify the points raised, and the manuscript is now clear and relevant to the community.

Reply: We sincerely thank the reviewer for the careful reading of our manuscript and for the constructive comments throughout the review process. We are grateful that the revisions and responses have clarified the raised concerns and improved the clarity of the manuscript.

Reviewer (Remarks to the Authors):

The authors report on the fractional quantum Hall phase diagram of pentalayer graphene. Pentalayer graphene has attracted significant interest recently, following the observation of fractional quantum anomalous Hall states and various superconducting states. The authors carefully isolated mixed-stack pentalayer graphene and provide the first experimental map of its quantum Hall phase diagram through conductance measurements.

This phase diagram is particularly interesting due to the appearance of numerous even-denominator fractional quantum Hall states, which are thought to host non-Abelian excitations useful for decoherence-free quantum computing. Their findings, compared with theoretical calculations, advance our understanding of quantum Hall physics in graphene-based devices. The study undoubtedly offers valuable insights for a broad audience, especially for those focused on graphene allotropes and quantum Hall physics. Consequently, I believe this study would be a fitting contribution to *Nature Communications*.

However, I recommend addressing a few points to sharpen the focus on the main findings and avoid possible misinterpretations of the data. The paper could have greater impact by further developing certain aspects, such as the characterization of the stacking order and the role of intertwined cubic and parabolic dispersions in the tunability of the quantum Hall phase diagram. I therefore ask the authors to answer the following questions and revise the manuscript accordingly, to make it clearer and more relevant to the community:

Stacking order identification

The identification of ABCBC stacking is not entirely clear. In Extended Data Figure 1, the authors state: “The brightest area is tentatively identified as a metastable stacking (e.g., ABCBC), though the exact structure remains uncertain from this characterization alone.”

However, this issue does not appear to be solved in the rest of the supplementary information, and it is unclear which measurement can unambiguously discriminate among metastable orders.

In the Methods section, it is stated that scanning near-field optical microscopy (SNOM) is used, but Extended Data Figure 1 shows near-field infrared, and Extended Data Figure 2 shows phonon-polariton assisted near-field scanning microscopy. Are these all considered part of the SNOM method?

Please label the scale bars in Extended Data Figures 1d and 2c so that we better understand what is measured exactly.

Is the final identification of the stacking order based on transport measurements?

I recommend adding a section titled **“Identification of the stacking order”** to make these points transparent and clear, since it is central to the paper.

Technical details

The use of the Si gate is mentioned to improve electrical contacts to the device. Please provide the value of the applied Si gate voltage in method, at least for the measurements in Main Text Figure 1. Please also comment on why the electron side (positive filling factor) is not studied at high field in the paper. If this is due to contact issues, please clarify.

Since graphite gate screening is crucial for understanding the effective Coulomb interaction and because it affects the fractional quantum Hall energy gaps, please provide the hBN thickness used in this device.

Abstract statement

The sentence "However, experimental platforms simultaneously offering large energy gaps, delicate tunability, and robust non-Abelian signatures remain scarce" is misleading, as the paper does not directly address these issues. Pentalayer graphene should simply be presented as a novel platform to study even-denominator fractional quantum Hall states.

Intertwined quadratic and cubic band dispersions

The band structure is emphasized twice:

"…a previously unexplored system characterized by intertwined quadratic and cubic band dispersions."
"…its band structure combines cubic and parabolic dispersions near the Fermi level with a small intrinsic gap (Fig. 1c, left), and undergoes a relative energy shift between the cubic and parabolic bands under external perpendicular displacement field D (Fig. 1c, right)."

It would be helpful to better explain the consequences of this feature for quantum Hall physics. Does it provide a tunable parameter, absent for example in bilayer graphene.

In Supplementary Fig. S1, some bands are pushed far in energy and do not contribute to the low-energy quantum Hall phase. Could the authors explicitly indicate which bands correspond to cubic or parabolic dispersion? Does these intertwined dispersions play a role in the high field quantum Hall phase diagram?

Landau level mixing

The manuscript states: "Unlike simpler graphene systems, the multiband nature of ABCBC-5LG amplifies LL mixing effects, which are critical for stabilizing correlated states at fractional fillings."

Please elaborate. In what sense does LL mixing stabilize correlated states? LL mixing is often expected to increase screening of the Coulomb interaction, which generally reduces the energy gap of fractional quantum Hall states.

Robustness and Unprecedented tunability claims

The claim of "unprecedented displacement-field tunability driven by LL crossings in the hybridized multiband structure" is underdeveloped. Previous multilayer graphene studies have already shown displacement-field tunability and transitions between even-denominator and Jain states. Please revise or develop this statement.

The manuscript states: "the highest half-filling even-denominator FQH states in ZLL reported so far, indicating the robustness afforded by the multilayer graphene platform." This argument is misleading: robustness should be characterized by the energy gap, which in this paper appears much smaller than in Bernal bilayer graphene even-denominator states. Please clarify why the highest filling factor should be seen as an advantage or remove the statement about robustness.

Comparison with similar graphene devices

While the article refers to an extensive body of literature on quantum Hall physics, many of the cited works concern earlier-generation graphene devices, which are not easily comparable to the state-of-the-art device quality studied here (i.e., hBN encapsulation and dual graphite gates). More recent studies focusing on fractional quantum Hall physics in graphene devices of similar quality to the one reported in this paper would be relevant. Phys. Rev. X **12**, 031019 (2022), Phys. Rev. Lett. **132**, 046603 (2024)*, Nat Commun* **16**, 7255 (2025).

CF effective mass

Screening and disorder can reduce the measured gap of fractional quantum Hall states (as shown again very recently *Nature* **644**, 646–651 2025), therefore I find that the comparison of the effective mass to GaAs CF state is not relevant : «comparable to values previously reported in GaAs » . A comparison of the effective mass to other dual gated hBN encapsulated graphene devices would be more relevant.

Phase transitions at half fillings

The manuscript states: "At half fillings, continuous quasiparticle phase transitions between paired FQH states, magnetic Bloch states, and composite Fermi liquids are clearly identified upon tuning external fields."

In fact, quasiparticle properties are not addressed experimentally. Figure 4 shows transitions, but the sharpness of these appear simply due to the presence or absence of an energy gap. This figure could be moved to the Supplementary Information. If it remains in the main text, the authors should better explain its intended message and revise the corresponding discussion.

Taken has it is, expanding the discussion on the observed daughter states of the even-denominator states, mentionned at the end of the previous paragraph, would be more relevant than the discussion about phase transition, as this paper focus on the « cascade of even-denominator states ».
